# Criminal justice interventions for preventing radicalisation, violent extremism and terrorism: An evidence and gap map

**DOI:** 10.1002/cl2.1366

**Published:** 2023-11-14

**Authors:** Michelle Sydes, Lorelei Hine, Angela Higginson, James McEwan, Laura Dugan, Lorraine Mazerolle

**Affiliations:** ^1^ Griffith Criminology Institute Griffith University Brisbane Australia; ^2^ School of Criminology and Criminal Justice Griffith University Brisbane Australia; ^3^ School of Social Science University of Queensland Brisbane Australia; ^4^ School of Justice, Faculty of Creative Industries, Education and Social Justice Queensland University of Technology Brisbane Australia; ^5^ Department of Sociology The Ohio State University Columbus Ohio USA

## Abstract

**Background:**

Criminal justice agencies are well positioned to help prevent the radicalisation of individuals and groups, stop those radicalised from engaging in violence, and reduce the likelihood of terrorist attacks. This Evidence and Gap Map (EGM) presents the existing evidence and gaps in the evaluation research.

**Objectives:**

To identify the existing evidence that considers the effectiveness of criminal justice interventions in preventing radicalisation, violent extremism and terrorism.

**Search Methods:**

We conducted a comprehensive search of the academic and grey literature to locate relevant studies for the EGM. Our search locations included the Global Policing Database (GPD), eight electronic platforms encompassing over 20 academic databases, five trial registries and over 30 government and non‐government websites. The systematic search was carried out between 8 June 2022 and 1 August 2022.

**Selection Criteria:**

We captured criminal justice interventions published between January 2002 and December 2021 that aimed to prevent radicalisation, violent extremism, and/or terrorism. Criminal justice agencies were broadly defined to include police, courts, and corrections (both custodial and community). Eligible populations included criminal justice practitioners, places, communities or family members, victims, or individuals/groups who are radicalised or at risk of becoming radicalised. Our map includes systematic reviews, randomised controlled trials, and strong quasi‐experimental studies. We placed no limits on study outcomes, language, or geographic location.

**Data Collection and Analysis:**

Our screening approach differed slightly for the different sources, but all documents were assessed in the systematic review software program DistillerSR on the same final eligibility criteria. Once included, we extracted information from studies using a standardised form that allowed us to collect key data for our EGM. Eligible systematic reviews were assessed for risk of bias using the AMSTAR 2 critical appraisal tool.

**Main Results:**

The systematic search identified 63,763 unique records. After screening, there were 70 studies eligible for the EGM (from 71 documents), of which two were systematic reviews (assessed as moderate quality), 16 were randomised controlled trials, and 52 were strong quasi‐experimental studies. The majority of studies (*n* = 58) reported on policing interventions. Limited evidence was found related to courts or corrections interventions. The impact of these interventions was measured by a wide variety of outcomes (*n* = 50). These measures were thematically grouped under nine broad categories including (1) terrorism, (2) extremism or radicalisation, (3) non‐terror related crime and recidivism, (4) citizen perceptions/intentions toward the criminal justice system and government, (5) psychosocial, (6) criminal justice practitioner behaviours/attitudes/beliefs, (7) racially targeted criminal justice practices, (8) investigation efficacy, and (9) organisational factors. The most commonly assessed outcomes included measures of terrorism, investigation efficacy, and organisational factors. Very limited research assessed intervention effectiveness against measures of extremism and/or radicalisation.

**Authors’ Conclusions:**

Conducting high‐quality evaluation research on rare and hidden problems presents a challenge for criminal justice research. The map reveals a number of significant gaps in studies evaluating criminal justice responses to terrorism and radicalisation. We conclude that future research should focus attention on studies that consolidate sound measurement of terrorism‐related outcomes to better capture the potential benefits and harms of counter‐terrorism programs, policies and practices which involve criminal justice agencies.

## 1 PLAIN LANGUAGE SUMMARY

### 1.1 The evidence for criminal justice interventions for preventing radicalisation, violent extremism and terrorism is unevenly distributed across criminal justice agencies, outcomes, and geographies

There is a relatively limited body of evidence related to criminal justice interventions for preventing radicalisation, violent extremism, and terrorism. Most of the evidence is focused on policing interventions and is situated in high‐income countries such as the USA.

### 1.2 What is this evidence and gap map (EGM) about?

Criminal justice agencies (police, courts and corrections) are well positioned to help prevent the radicalisation of individuals and groups, to stop those radicalised from engaging in violence, and to reduce the likelihood of terrorist attacks.

Decisionmakers need to know what the available evidence is and what remains unstudied. This EGM shows the existing evidence and gaps in the evaluation research, allowing stakeholders to identify future funding priorities for research and programme development.

**What is the aim of this EGM?**

The aim of this EGM is to show all the available evidence from systematic reviews and high‐quality impact evaluations of criminal justice interventions that seek to prevent radicalisation, violent extremism and terrorism.

### 1.3 What studies are included?

The EGM includes impact evaluations and systematic reviews assessing the effect of criminal justice interventions on preventing radicalisation, violent extremism and/or terrorism.

Included studies had to report an estimate of the quantitative impact of an intervention. The studies were broadly categorised as to whether the intervention involved police, courts, corrections (custodial and community‐based) and if they were multi‐agency, involving at least one criminal justice partner.

No limits were placed on outcomes. Outcomes were broadly categorised under nine thematic groups: terrorism; extremism/radicalisation; non‐terror related crime and recidivism; citizen perceptions/intentions toward the criminal justice system and government; psychosocial factors; criminal justice practitioner beliefs, attitudes and/or behaviours; racially targeted criminal justice practices; investigation efficacy and organisational factors.

The map includes 70 studies from 71 documents: two systematic reviews and 68 impact evaluations. The included impact evaluations are predominantly quasi‐experimental studies.

### 1.4 What are the main findings of this map?

The studies are unevenly distributed across criminal justice agencies. Policing is the most heavily populated area of the map, representing 58 of the 70 studies. There are relatively few studies that evaluate courts and prison interventions for preventing terrorism/radicalisation. No studies evaluate the impact of community corrections interventions.

The most common outcome measures are related to terrorism (*n* = 20), including terrorism incidents *(n* = 14), fatalities caused by terrorism (*n* = 4), citizen willingness to report terrorism (*n* = 2) and diffusion/displacement effects (*n* = 1).

Investigation efficacy (for example detection of guilt, detection of suspicious activities, and information sharing) and organisational factors (such as inter‐agency collaboration and uptake of Homeland Security initiatives) are also frequently evaluated. However, very few studies evaluate intervention impact using measures of radicalisation/extremism.

The majority of studies included on the EGM are situated in the USA or other high‐income countries.

### 1.5 What do the findings of the map mean?

This EGM aims to provide a comprehensive and systematic display of the existing evidence on criminal justice interventions which aim to prevent radicalisation, violent extremism and/or terrorism. By locating and visually presenting all available evidence, the EGM improves access to high‐quality impact evaluations.

We suggest future counter‐terrorism research pay attention to building researcher‐practitioner partnerships that focus on scientific quality, with a clear agenda to build a more robust evidence base.

### 1.6 How up‐to‐date is this EGM?

The search was carried out between June and August 2022. The authors searched for studies published up to December 2021.

## 2 BACKGROUND

### 2.1 The problem, condition or issue

Radicalisation, violent extremism and terrorism continue to represent an ongoing threat to global security, with 42 countries experiencing at least one death from terrorism in 2022 (Institute for Economics and Peace [IEP], 2023) While definitions vary (Borum, 2015; Desmarais et al., 2017; Horgan, 2008; Kruglanski, Bélanger, & Gunaratna, 2019; Sarma, 2017) violent radicalisation is broadly defined as the process by “which a person adopts extremist views and moves toward committing a violent act” (Hardy, 2018, p. 76; see also Irwin, 2015; Jensen, Atwell Seate, & James, 2020). Once radicalised, individuals and groups *may* go on to carry out terrorist attacks against innocent civilians, key infrastructure, symbolic targets or specific groups (Wilner & Dubouloz, 2010) or travel to conflict zones to engage in violence with extremist groups (Lindekilde, Bertelsen, & Stohl, 2016). Preventing violent radicalisation has thus become a key focus of counterterrorism agendas globally (Dudenhoefer, 2018; Elshimi, 2017; Heath‐Kelly, 2013).

The harms associated with violent radicalisation and terrorism are far‐reaching. In 2022, the Global Terrorism Index recorded 6,701 terrorism related deaths and 3,955 terror‐related attacks (IEP, 2023). The estimated economic impact of terrorism is similarly staggering. According to Bardwell and Iqbal (2020), the cost of terrorism to the world economy between 2000 and 2018 was US $855 billion. Beyond the human casualties and economic consequences, violent radicalisation and terrorism are linked to a broad range of psychological, social and political impacts. Studies find experiences of terrorism can have negative mental health implications particularly among first responders and victims who face high levels of trauma and depression in the aftermath (Razik, Ehring, & Emmelkamp, 2013; Salguero et al., 2011). Violent radicalisation and terrorism can also antagonise public attitudes toward immigration and groups perceived as a terrorist threat (Williamson & Murphy, 2020). Right‐wing political parties frequently manipulate fear of terrorism and violent radicalisation to garner support for hard‐line and discriminatory counterterrorism policies (Haner et al., 2019, 2021; Williamson, 2019). Such policies often result in the over‐surveillance of ‘suspect communities’ (Breen‐Smyth, 2014; Cherney & Murphy, 2016), further isolating groups at risk of radicalisation and in turn, providing motivation for retaliatory attacks (Fisher, Dugan, & Chenoweth, 2018; Jacobs & van Spanje, 2021).

Research suggests that individual pathways to radicalisation can be influenced by a broad range of factors including social networks, affiliation with certain political organisations, places of worship, familial relationships or exposure to radical individuals during incarceration. These predictors can also interact with personal factors such as psychological disorders or traumatic experiences, further heightening the risk of radicalisation (Jensen et al., 2020; Kruglanski et al., 2019). Effectively intervening to prevent and reduce the likelihood of violent radicalisation and terrorism is therefore often difficult due to the significant variation in the predictors of violent radicalisation amongst individuals and extremist groups (Desmarais et al., 2017; Wolfowicz, Litmanovitz, Weisburd, & Hasisi, 2019).

### 2.2 The intervention

Criminal justice agencies (i.e., police, courts and corrections) are well positioned to help prevent the radicalisation of individuals and groups, stop those radicalised from engaging in violence, and reduce the likelihood of terrorist attacks. Following September 11, police became more actively involved in countering terrorism—disrupting terrorist activity through intelligence gathering, covert investigations, information sharing and enforcement activities (Ortiz, Hendricks, & Sugie, 2007). As frontline practitioners, police are also instrumental in identifying, reducing, and preventing both radicalisation and violent extremism (Mazerolle et al., 2021). Police approaches for preventing radicalisation and violent extremism involve a range of different interventions and strategies. These include police facilitating training programs to improve officer recognition and responses to radicalisation or police delivering awareness programs to educate others on how to identify and report radicalised individuals (Carter, 2014; Davis et al., 2016). Some police agencies also work with communities to build resilience to protect against the influence of extremist messages and recruiters (Cherney & Hartley, 2017; Mazerolle et al., 2020; Schanzer, Kurzman, Toliver, & Miller, 2016). Such initiatives focus on bolstering community engagement, trust and connectedness while reducing feelings of social isolation (Mazerolle et al., 2020).

The courts and correctional agencies also play a critical role in preventing radicalisation, violent extremism and terrorism. Court officials may direct radicalised individuals or individuals who have engaged in violent extremism and/or terrorist activity to various support services or mandate participation in rehabilitation programs designed to counter violent extremism (Cherney, De Rooy, & Eggins, 2021). Prisons are widely believed to provide a prime opportunity for radicalisation to flourish, threatening the safety and security of both inmates and the community more broadly (van der Heide & Kearney, 2020). Training initiatives and awareness programs with corrective services staff may assist in the detection and prevention of both radicalisation and violent extremism (Vejvodová & Kolář, 2020). In an attempt to prevent the spread of radicalised ideologies behind bars, corrective services agencies often segregate radicalised individuals from the general prison population (Jones & Narag, 2018). Indeed, some jurisdictions operate specialised terrorist units to house extremist offenders (van der Heide & Kearney, 2020), with the aim to prevent radicalised inmates from influencing other prisoners and allow staff interacting with radicalised inmates to be upskilled in best practice approaches. Corrective services agencies may also design and deliver deradicalisation programs to radicalised inmates or inmates convicted of terror‐related offences (Cherney & Belton, 2021). Upon release, community corrections staff may provide radicalised offenders with the support necessary for successful reintegration back into the community (Cherney, 2021).

Recognising the complexities surrounding radicalisation and terrorism, many criminal justice agencies are increasingly taking an intersectoral and multi‐agency approach. Such approaches acknowledge the difficulties a single agency or organisation may face in trying to combat the problem in isolation (Mazerolle et al., 2021). These initiatives include promoting information sharing between agencies (Knight, 2009), community outreach activities (Lamb, 2013; Schanzer et al., 2016), intelligence gathering (Lewandowski, 2012) and training programs (Davis et al., 2016; Kerry, 2007). For example, the World Organisation for Resource Development Education (WORDE) program which was led by a non‐profit group of Muslim scholars and community leaders, partnered with several agencies including law enforcement. The program comprised three key components: (1) community education via town hall meetings; (2) the development of a referral network for high‐risk individuals; and (3) participation in community activities (Williams et al., 2016). By partnering with other agencies and community groups, the intervention aimed to tackle the problem of radicalisation in a multi‐faceted manner (Beutel & Weinberger, 2016; Mazerolle et al., 2021).

Criminal justice interventions to prevent radicalisation, violent extremism and terrorism thus cover a broad range of policies and programs (Hardy, 2020). Prevention efforts can be divided into primary prevention (interventions targeted at a population), secondary prevention (interventions targeted at high‐risk individuals) and tertiary prevention (interventions targeted at already radicalised individuals) (Harris‐Hogan, Barrelle & Zammit, 2016). Criminal justice interventions that respond to terrorism/radicalisation are thus delivered across a variety of settings with participants ranging from community members to convicted terrorists (Gielen, 2019). As such, there is no singular theoretical framework underpinning criminal justice interventions for preventing terrorism and radicalisation. Prevention efforts by criminal justice agencies vary considerably in their underlying mechanisms and intended outcomes (Hardy, 2020). For instance, primary intervention programs may focus on building community capacity/cohesion and feelings of inclusion (Mazerolle et al., 2020), secondary prevention programs may aim to reduce vulnerability or risk of radicalisation (Jones & Narag, 2018) and tertiary prevention programs may seek to deradicalise convicted extremists (Cherney & Belton, 2021).

### 2.3 Why it is important to develop the EGM

Extensive research suggests criminal justice agencies worldwide have implemented and evaluated a vast range of programs, policies and practices aimed at preventing radicalisation, violent extremism and terrorism (Mazerolle et al., 2021). However, in many cases, practitioners working in counterterrorism cannot easily access the evaluation evidence. This can lead to a significant disconnect between the existing evidence base and what is implemented in practice (Koehler & Fiebig, 2019). This project helps bridge this gap by creating an interactive EGM.

Built using data collected via systematic search methods, EGMs provide a visual display of the available research evidence related to a particular topic area (White et al., 2020). EGMs are typically presented as a matrix comprising intervention categories (rows) and outcomes (columns). Interactive EGMs may offer additional filters which allow users to view the information based on certain classifications (e.g., study location, document type, study quality) (White et al., 2020). By locating and visually presenting all available evidence, EGMs improve stakeholder access to impact evaluations. While EGMs are a relatively new method of evidence synthesis, they are increasingly used to map evidence related to crime and justice issues (see e.g. Eggins et al., 2021; Pundir et al., 2020).

There are currently no existing EGMs that focus on criminal justice responses to radicalisation, violent extremism, and/or terrorism. Several systematic reviews have synthesised the effectiveness literature. However, these reviews have either had a broad scope including criminal justice agencies amongst other agencies (Lum, Kennedy, & Sherley, 2006) or a more targeted scope focusing on just a particular branch of the criminal justice system (see e.g., Mazerolle et al., 2021 for multi‐agency responses to radicalisation with police as a partner). Other reviews have focused on a particular type of program. For example, Mazerolle et al. (2020) reviewed police programs that seek to increase community connectedness for reducing violent extremism behaviour, attitudes and beliefs. Our systematic search will include all types of criminal justice interventions that aim to prevent radicalisation, violent extremism and/or terrorism. It is expected that this EGM will help promote the translation of evidence into practice by providing practitioners and policymakers with easy access to evaluation evidence.

This EGM will be used to demonstrate gaps in the evaluation research. This will allow key stakeholders to identify future funding priorities for research and development. Specifically, if the EGM indicates a lack of research related to a particular intervention/outcome, this may provide grounds to justify future primary studies. Additionally, if the EGM illustrates several studies in one domain, this may lead to further syntheses such as meta‐analysis.

## 3 OBJECTIVES

This EGM aims to provide a comprehensive and systematic display of the existing evidence on criminal justice interventions which aim to prevent radicalisation, violent extremism, and terrorism. It will map the extant evaluation evidence (systematic reviews, randomised experiments and strong quasi‐experimental designs) across the policing, courts, and corrections arms of the criminal justice system including multi‐agency responses. The key objectives of this EGM are as follows:

1. To identify the existing evidence that considers the effectiveness of criminal justice interventions in preventing radicalisation, violent extremism and terrorism.
2. To identify existing gaps in the evidence where new primary research could be undertaken and where future synthesis could be conducted.

## 4 METHODS

### 4.1 Evidence and gap map: Definition and purpose

EGMs provide an interactive and graphical representation of the available evidence concerning a particular topic area (Snilsveit et al., 2016). EGMs are usually presented as a matrix with interventions displayed in rows and outcomes presented in columns (White et al., 2020). Like systematic reviews, EGMs utilise rigorous systematic search methods (White et al., 2020). However, EGMs are usually broader in scope than systematic reviews. EGMs are often viewed as ‘public goods’ as they improve accessibility to high quality evidence for policymakers, practitioners and the wider public (Snilsveit et al., 2016). Our EGM highlights both the density and paucity of existing research by including both primary studies and systematic reviews related to criminal justice interventions for preventing radicalisation, violent extremism, and terrorism and radicalisation.

### 4.2 Framework development and scope

The framework used in the EGM is both theoretically and empirically informed. The initial framework to design inclusion and exclusion criteria and search strategies was based on an a priori schema, described below, and was informed by the practical experiences and academic knowledge of the research team. Building on this framework, we conducted a thematic cluster analysis of the included studies to develop an empirical classification schema for the final EGM categories and domains.

### 4.3 Stakeholder engagement

Initial stakeholder consultation took place with the Department of Homeland Security in July 2021 to determine the outcome categories of interest and EGM scope. We also invited stakeholders from various government agencies across the United States, United Kingdom, Australia, New Zealand, Canada and Sweden to join a virtual Advisory Group, as well as researchers from Spain and Israel. Given project time constraints, stakeholder feedback was challenging. A link to the EGM was disseminated with a request for comment on the functionality, thematic coding and included studies. We additionally leveraged professional networks to request feedback from both academic and non‐academic experts in the radicalisation/terrorism space.

### 4.4 Dimensions

Our EGM is presented as a matrix of interventions (rows) and outcomes (columns). The number of primary studies is shown by the size of the bubble. The dimensions of the map were developed following a thematic analysis of the eligible studies. We first categorised interventions by criminal justice branch (i.e., police, courts, custodial corrections, community corrections and multi‐agency) and then we thematically grouped similar interventions (e.g., community policing initiative, interrogation techniques, early alert systems). Similar outcomes were also thematically grouped (e.g., radicalisation indicators, investigation efficacy).

#### 4.4.1 Types of study design

Systematic reviews and randomised controlled trials (RCTs) are considered the gold standard for ascertaining intervention effectiveness. Eligible comparison conditions for inclusion in the EGM include no treatment, placebo, ‘business as usual’, waitlist control, or an alternative treatment. While other designs are less causally robust, they may be appropriate to include due to the difficulties associated with conducting RCTs in criminal justice settings, particularly when targeting radicalisation, violent extremism, and/or terrorism. The strong quasi‐experimental designs eligible for this EGM include:

- Matched control group designs with or without preintervention baseline measures (propensity or statistically matched)
- Matched control group designs without preintervention baseline measures where the control group has face validity
- Unmatched control group designs with pre–post intervention measures that allow for difference‐in‐difference analysis
- Unmatched control group designs without pre–post intervention measures that allow for difference‐in‐difference analysis
- Regression discontinuity designs
- Cross‐over designs
- Designs using multivariate controls (e.g., multiple regression)
- Short interrupted time series designs with control group (<25 preintervention and 25 postintervention observations [Glass, 1997])
- Long interrupted time series designs with or without a control (≥25 preintervention and postintervention observations [Glass, 1997]).

In line with previous reviews (Mazerolle et al., 2020, 2021) that recognise the significant shift in counterterrorism policies post 9/11 (Gaibulloev & Sandler, 2019), only studies that were published from or report on impact evaluations conducted between January 2002 and December 2021 were included in the EGM. Qualitative research was not included as we were exclusively interested in mapping high quality impact evaluations. There were no limitations on publication status.

#### 4.4.2 Types of intervention/problem

Our EGM captures criminal justice interventions that aim to prevent radicalisation, violent extremism, and/or terrorism.

A *criminal justice intervention* was defined as a strategy, technique, approach, activity, campaign, training, directive, funding, or organisational change that involved at least one criminal justice agency. Criminal justice agencies include police, courts and corrections (both custodial and community based). Interventions that involve partnerships between at least one criminal justice agency and at least one external agency/organisation were also included (Eggins et al., 2021; Mazerolle et al., 2018). Examples of criminal justice interventions may include:

- Criminal justice system initiation, development, or leadership of the intervention
- Criminal justice system staff or populations as recipients of the intervention
- Criminal justice system practices as the focus or target of the intervention
- Criminal justice system delivers or implements the intervention.

Intelligence agencies (e.g., the CIA, ASIO, MI5), security agencies (e.g., DHS), foreign affairs (e.g., US Department of State) and the military (with the exception of military police) were not considered criminal justice agencies. Interventions that involved these agencies were only eligible if they included a partnership with police, courts or corrections.

Similarly, the passing of legislation was not considered a criminal justice intervention, unless the study focused on the *implementation* of legislation by criminal justice agencies. For example, studies that examined the effect of the decision to charge a suspect under a terrorism offence or under a general criminal offence after the passing of anti‐terrorist legislation were eligible, as the charging decision was made by a criminal justice agency. Alternatively, studies that looked at the number of terrorist attacks before and after the passing of anti‐terrorist legislation were not eligible, as the passing of laws is not an intervention available to criminal justice agencies.

While definitions of terrorism, violent extremism and radicalisation vary (Sinai, 2008), we define the concepts broadly below:

- *Radicalisation* was defined as the process of an individual or group adopting extreme political, social, or religious ideals (Hardy, 2018; Jensen et al., 2020). Analogous concepts are *disengagement* and/or *deradicalisation*, which are often encompassed within conceptualisations of violent extremism. *Disengagement* refers to reducing or ceasing physical involvement in violent or radical activities, while *deradicalisation* is defined as the psychological shift in attitudes or beliefs (Windisch, Simi, Ligon, & McNeel, 2016).
- *Violent extremism* was defined as ‘advocating, engaging in, preparing, or otherwise supporting ideologically motivated or justified violence to further social, economic, and political objectives’ (US Agency for International Development, 2016, p. 2).
- *Terrorism* was initially defined as “the unlawful use of force or violence against persons or property to intimidate or coerce a government, the civilian population or any segment thereof, in furtherance of political or social objectives” (Shanahan, 2016, p. 108). However, our pilot screening for the EGM discovered studies addressing bio‐terrorism (see Green, LeDuc, Cohen, & Franz, 2019; Laufs & Waseem 2020) and cyber‐terrorism (see Harkin & Whelan, 2022). These were two categories of counterterrorism interventions we did not anticipate coding for in the protocol (see Sydes et al., 2022). As such, we made an early decision to alter the protocol coding scheme to include interventions that targeted bio‐ and cyber‐terrorism. We defined bio‐terrorism as ‘…the use of biological agents to inflict disease and/or death on humans, animals or plants’ (Green et al., 2019; Klietmann & Ruoff, 2001, p. 364). We described cyber terrorism as ‘…the premeditated attack or threat thereof by non‐state actors with the intent to use cyberspace to cause real‐world consequences in order to induce fear or coerce civilian, government, or non‐government targets in pursuit of social or ideological objectives’ (Plotnek & Slay, 2021, p. 8). Our final definition of terrorism encompassed both our initial definition as well as those for bio‐ and cyber‐terrorism.

We anticipated that criminal justice interventions for preventing radicalisation, violent extremism and/or terrorism could potentially include the following:

- Police training programs to improve officer recognition and responses to radicalisation, violent extremism and terrorism (Davis et al., 2021)
- Police working with communities to build resilience to protect against the influence of extremist messages and recruiters (Cherney & Hartley, 2017; Mazerolle et al., 2020; Schanzer et al., 2016)
- Court officials mandating participation in rehabilitation programs designed to counter violent extremism (Cherney et al., 2021)
- Corrections agencies installing terrorist wings within prisons to separate radicalised prisoners or prisoners convicted of terrorist acts from the general prison population (van der Heide & Kearney, 2020)
- Corrections agencies delivering specialised and individualised rehabilitation programs designed to challenge radicalised views (Cherney & Belton, 2021)
- Training initiatives and awareness programs with corrective services staff focused on the detection and prevention of both radicalisation and violent extremism (Vejvodová & Kolář, 2020)
- Re‐entry programs focused on building pro‐social networks and access to support services to assist with prisoner release (Cherney, 2021)
- Multi‐agency initiatives that promote information sharing and intelligence gathering between agencies (Knight, 2009; Lewandowski, 2012)
- Multi‐agency initiatives such as community outreach activities (Lamb, 2013; Schanzer et al., 2016)
- Multi‐agency initiatives focused on delivering training to various stakeholders on how to identify and report radicalised individuals (Carter, 2014; Davis et al., 2016; Kerry, 2007).

If a study reported on multiple interventions whereby only a subset met the eligibility criteria, only the eligible interventions were included in the map.

#### 4.4.3 Types of population

The following populations were eligible for this EGM:

- Criminal justice practitioners
- Victims
- Communities
- Individuals or groups who have been identified as at risk of becoming radicalised or engaging in violent extremism and/or terrorist activity
- Radicalised individuals or groups (including pre‐criminal justice involved radicalised individuals)
- Individuals or groups who have engaged in violent extremism and/or terrorist activity
- Family members of radicalised individuals or individuals who have engaged in violent extremism and/or terrorist activity
- Micro places (e.g., street corners, buildings, police beats, street segments)
- Macro places (neighbourhoods or larger geographies)
- Agencies or organisations.

#### 4.4.4 Types of outcome measures

No restrictions were placed on the types of outcomes used to evaluate criminal justice responses for preventing radicalisation, violent extremism and/or terrorism. Unintended or adverse outcomes were included.

##### Types of location/situation

We placed no limits on the geographical region reported in the study. No restrictions were placed on the language a document was written in. Similar to other reviews (e.g., Davis et al., 2021; Mazerolle et al., 2020), titles/abstracts written in a language other than English were translated using Google Translate to determine potential eligibility for the EGM. Full‐text documents in a language other than English were translated using Google Translate to ascertain eligibility. For studies we could not determine eligibility through this approach, the authors were contacted directly where possible.

##### Types of settings

No restrictions were placed on the settings used in eligible studies.

#### 4.4.5 Search methods and sources

##### Global policing database

The policing literature search was extracted from the Global Policing Database (GPD). The University of Queensland is home to the GPD (see http://www.gpd.uq.edu.au), a web‐based and searchable database designed to capture all published and unpublished experimental and quasi‐experimental evaluations of policing interventions conducted since 1950. The GPD has no restrictions on the type of policing technique, type of outcome measure or the language of the research. The GPD is compiled using systematic search and screening techniques, which are reported in Higginson et al. (2015) and summarised in Supporting Information: Appendices 1 and 2. Broadly, the GPD search protocol includes an extensive range of search locations to ensure that both published and unpublished research is captured across criminology and allied disciplines.

To capture studies for this review, we used terms related to radicalisation, violent extremism and terrorism to search the GPD corpus of full‐text documents that have been screened as reporting on a quantitative impact evaluation of a policing intervention. Specifically, we used the following terms to search the title and abstract fields of the corpus of documents published between 1 January 2002 and 31 December 2021. This search was completed on 1st August 2022 (the latest date of the search across all databases).

extremis* OR ‘far#left*’ OR ‘far#right*’ OR ‘foreign#fight*’ OR ‘freedom#fight*’ OR guerrilla OR ‘homeland#security’ OR ‘ideological violence*’ OR ‘ideologically#motivat*’ OR indoctrinat* OR ‘left#wing*’ OR ‘lone#wol*’ OR militant* OR ‘national#security’ OR ‘political violence*’ OR ‘politically#motivat*’ OR radicali* OR rebel* OR ‘religious violence*’ OR ‘religiously#motivat*’ OR ‘right#wing*’ OR ‘single#issue*’ OR supremacis* OR terror* OR vigilante* OR vigilantism OR deradicali* OR ‘de‐radicali*’ OR ‘counter‐terror*’ OR counterterror* OR ‘counter‐extremis*’ OR counterextremis* OR separatis* OR militia* OR jihad*

##### Other database searches

To capture interventions in the courts, corrections and multi‐agency spheres, we conducted searches of key academic sources in criminology and criminal justice (see Table 1). We searched these databases between 8 June and 29 July 2022 on three sets of terms across title, abstract, keyword, and/or subject/indexing fields with the aim to capture literature on (1) radicalisation/terrorism within the (2) criminal justice system that uses a term indicative of a (3) evaluation of an intervention. Terms within each set were combined with Boolean OR operators, and the three sets were then combined with Boolean AND operators (see the full Supporting Information: Appendix 3). This search approach was informed by Eggins et al. (2021) systematic review of criminal justice system responses to child sexual abuse material offending. We searched the following across the title, abstract, keyword, and/or subject/indexing fields (adapting the search to the database if fields were unavailable):

**Table 1:** Search plan.

Source	Approach	Website (if applicable)
*Academic databases*		
EBSCO Criminal Justice Abstracts	Terms: Full search string Fields: Title, abstract, keywords	
Global Policing Database	Terms: Terrorism terms only Fields: Title, abstract	
HeinOnline Criminal Justice & Criminology Foreign and International Law Resources Database Law Journal library United Nations Law Collection	Terms: Full search string Fields: Text, title, subjects	
Informit AGIS Plus Text Australian Criminology DB (CINCH)	Terms: Full search string with databases searched simultaneously Fields: Title, abstract, subjects	
National Criminal Justice Reference Service	Terms: We accessed a free website where we conducted basic keyword searches for terrorism terms. Results were web‐scraped. Fields: General search, title	
OVID PsycEXTRA PsycINFO	Terms: Full search string Fields: Title, abstract, keywords, subject	
ProQuest Criminal Justice Dissertations & These Global Digital National Security Archive PTSDPubs Social Science Database Sociological Abstracts (incl. Social Services Abstracts)	Terms: Full search string with databases searched simultaneously Fields: Title, abstract, keywords, subject	
Web of Science Social Science Citation Index Conference Proceedings Index (Social Sciences & Humanities) Emerging Sources Index SciELO Citation Index MEDLINE	Terms: Full search string searched Fields: Title, abstract, keywords, subject	
*Research repositories/websites*		
Australian Capital Territory Corrective Services	Hand searched for terrorism intervention literature	http://www.cs.act.gov.au/act_corrective_services/stats_and_publications
Campbell Collaboration—terrorism reviews	Hand searched for terrorism intervention reviews	https://onlinelibrary.wiley.com/journal/18911803
Centre for Advancing Correctional Excellence	Hand searched for terrorism intervention literature	https://www.gmuace.org/
Centre of Excellence Defence Against Terrorism	Hand searched for criminal justice intervention literature	https://www.tmmm.tsk.tr/research.html
Cochrane Library (including Cochrane Central Register of Controlled Trials [CENTRAL])	Terms: Full search string searched Fields: Title, abstract, keywords	https://www.cochranelibrary.com/search
Combating Terrorism Center	Hand searched for criminal justice intervention literature	https://ctc.usma.edu/ctc-sentinel/
Correctional Service Canada	Hand searched for terrorism intervention literature	http://www.csc-scc.gc.ca/research/005008-2006-eng.shtml
CrimeSolutions.Gov	Hand searched for terrorism intervention literature in the corrections subsets	https://crimesolutions.ojp.gov/
Department of Homeland Security	Hand searched for criminal justice intervention literature	https://www.dhs.gov/topics
Hedayah	Hand searched for criminal justice intervention literature	http://www.hedayahcenter.org/publications
International Association of Law Enforcement Intelligence Analysts	Hand searched for terrorism intervention literature	https://www.ialeia.org/resources_publications.php
International Centre for Counter‐Terrorism	Hand searched for criminal justice intervention literature	https://icct.nl/topic/criminal-justice-response/
Impact Europe	Hand searched for criminal justice intervention literature	http://impacteurope.eu/
International Centre for the Study of Radicalisation	Hand searched for criminal justice intervention literature	https://icsr.info/publications/
Global Centre on Cooperative Security	Hand searched for criminal justice intervention literature	https://www.globalcenter.org/publications/
Global Terrorism Research Centre (Monash University)	Hand searched for criminal justice intervention literature	https://www.monash.edu/arts/social-sciences/gtrec/publications
National Consortium for the Study of Terrorism and Responses to Terrorism (START)	Hand searched for criminal justice intervention literature	https://www.start.umd.edu/
National Institute of Corrections	Hand searched for terrorism intervention literature	https://nicic.gov/
Naval Post‐Graduate School	Hand searched for terrorism intervention literature	https://nps.edu/web/research/home
New South Wales Corrective Services	Hand searched for terrorism intervention literature	http://www.correctiveservices.justice.nsw.gov.au/Pages/CorrectiveServices/related-links/publications-and-policies/corrections-research-evaluation-and-statistics/Research_Publication.aspx http://www.correctiveservices.justice.nsw.gov.au/Pages/CorrectiveServices/related-links/publications-and-policies/corrections-research-evaluation-and-statistics/collaborative-reports.aspx
New Zealand Corrective Services	Hand searched for terrorism intervention literature	http://www.corrections.govt.nz/resources/research_and_statistics.html
Northern Territory Corrective Services	Hand searched for terrorism intervention literature	https://justice.nt.gov.au/attorney-general-and-justice/justice-publications
Prison Research Centre	Hand searched for terrorism intervention literature	https://www.prc.crim.cam.ac.uk/publications/articles
Public Safety Canada	Hand searched for criminal justice intervention literature	https://www.publicsafety.gc.ca/index-en.aspx
Queensland Corrective Services	Hand searched for terrorism intervention literature	https://corrections.qld.gov.au/documents/reviews-and-reports/
Radicalisation Awareness Network (RAN)	Hand searched for criminal justice intervention literature	https://ec.europa.eu/home-affairs/what-we-do/networks/radicalisation_awareness_network_en
Radicalisation Research	Hand searched for criminal justice intervention literature	https://www.radicalisationresearch.org/
RAND	Hand searched for terrorism intervention literature in the Better Policing Toolkit, the Courts and Corrections sections	https://www.rand.org/pubs/tools/TL261/better-policing-toolkit.html https://www.rand.org/jie/justice-policy/pubs/courts.html https://www.rand.org/jie/justice-policy/correctional-education.html https://www.rand.org/jie/justice-policy/projects/priority-criminal-justice-needs.html
Royal United Services Institute (RUSI)	Hand searched for criminal justice intervention literature	https://rusi.org/
South Australia Corrective Services	Hand searched for terrorism intervention literature	https://www.corrections.sa.gov.au/about/our-research
Tasmanian Corrective Services	Hand searched for terrorism intervention literature	https://www.justice.tas.gov.au/
Terrorism Research Centre	Hand searched for criminal justice intervention literature	http://www.terrorism.org/
Triangle Centre on Terrorism and Homeland Security	Hand searched for criminal justice intervention literature	https://sites.duke.edu/tcths/#
UK Government research	Hand searched for terrorism intervention literature. Policing literature was already captured in GPD for this site, so the search targeted the courts/corrections agencies	https://www.gov.uk/search/research-and-statistics
Urban Institute	Hand searched for terrorism intervention literature	https://www.urban.org/policy-centers/justice-policy-center/publications
Victorian Corrections, Prisons and Parole	Hand searched for terrorism intervention literature	https://www.corrections.vic.gov.au/publications-manuals-and-statistics
Western Australia Corrective Services	Hand searched for terrorism intervention literature	https://www.correctiveservices.wa.gov.au/about-us/statistics-publications/default.aspx
What Works Toolkit	Hand searched for terrorism intervention literature	http://whatworks.college.police.uk/toolkit/Pages/Toolkit.aspx
*Trial registries*		
AEA RCT Registry	Terms: Modified search due to database functionality, focused on terrorism search terms Fields: Title, abstract, keywords	https://www.socialscienceregistry.org/
NIH RePORTER (clinical studies)	Terms: Modified search due to database functionality, focused on terrorism search terms Fields: Title, abstract, keywords	https://reporter.nih.gov/
World Health Organization International Trial Registry	Terms: Full search string searched Fields: Title, abstract, keywords	https://trialsearch.who.int/
Trials Register of Promoting Health Interventions (TRoPHI)	Terms: Modified search due to database functionality, focused on terrorism search terms Fields: Title, abstract, keywords	https://eppi.ioe.ac.uk/webdatabases4/Intro.aspx?ID=12
*Journals*		
Journal of 9/11 Studies	Hand searched for criminal justice intervention literature	http://www.journalof911studies.com/J911S/articles/
Journal of Security, Intelligence and Resilience Education	Hand searched for criminal justice intervention literature	https://jsire.org/about/
Journal of Terrorism Research	Hand searched for criminal justice intervention literature	https://cvir.st-andrews.ac.uk/articles/search/
Jstor journals: Counter Terrorist Trends & Analysis (CTTA) International Journal of Peace Studies Prism	Terms: Targeted search within journal in Jstor. Searched on courts/corrections and evaluation terms Fields: Title, abstract, keywords	
The Journal of International Security Affairs	Hand searched for criminal justice intervention literature	https://security-affairs.com/about/

(1) extremis* OR ‘far#left*’ OR ‘far#right*’ OR ‘foreign#fight*’ OR ‘freedom#fight*’ OR guerrilla OR ‘homeland#security’ OR ‘ideological violence*’ OR ‘ideologically#motivat*’ OR indoctrinat* OR ‘left#wing*’ OR ‘lone#wol*’ OR militant* OR ‘national#security’ OR ‘political violence*’ OR ‘politically#motivat*’ OR radicali* OR rebel* OR ‘religious violence*’ OR ‘religiously#motivat*’ OR ‘right#wing*’ OR ‘single#issue*’ OR supremacis* OR terror* OR vigilante* OR vigilantism OR deradicali* OR ‘de‐radicali*’ OR ‘counter‐terror*’ OR counterterror* OR “counter‐extremis*’ OR counterextremis* OR separatis* OR militia* OR jihad*

AND

(2) accused OR acquit* OR adjourn* OR adjudicat* OR *admiss* OR affida* OR appeal* OR appellate OR apprehend* OR arbitrat* OR arraign* OR *arrest* OR attorney* OR authorit* OR bail* OR barrister* OR breach* OR ‘case#manage*’ OR caution* OR charge* OR clerk* OR confinement* OR convict* OR coroner* OR correction* OR court* OR crime* OR criminal* OR ‘cross#examin*’ OR custod* OR defendant* OR defense OR defence OR detain* OR detention* OR deter* OR divert* OR diversion* OR enforc* OR execut* OR felon* OR forensic* OR gaol* OR guilt* OR ‘high#security’ OR ‘halfway#house’ OR *imprison* OR incarcerat* OR indict* OR infract* OR infring* OR injunct* OR inquest* OR innocen* OR inmate* OR juris* OR jail* OR judge* OR judic* OR juror* OR juries OR jury OR justice OR law* OR legal* OR legislat* OR litigat* OR ‘low#security’ OR magistrate* OR mandat* OR mitigat* OR marshal* OR misdem* OR ‘medium#security*’ OR offend* OR offence* OR officer* OR official* OR ordinance OR parole* OR pardon* OR penal* OR plea* OR precedent* OR prevent* OR prison* OR probat* OR prohibit* OR prosecut* OR punish*OR recividis* OR rehab* OR reintegrat* OR remand* OR reoffend* OR ‘re‐offend*’ OR ruling* OR sanction* OR sentenc* OR solicitor* OR statut* OR subpoena* OR supervis* OR surveil* OR suspect* OR testif* OR testimon* OR *trial* OR tribunal* OR verdict* OR victim*OR witness*

AND

(3) ‘comparison#condition*’ OR ‘comparison#group*’ OR ‘control#condition*’ OR ‘control#group*’ OR effective OR efficac* OR evaluat* OR experiment* OR intervent* OR ‘matched#group*’ OR program* OR ‘quasi#experiment*’ OR random* OR RCT OR treatment* OR trial*

#### 4.4.6 Searching other resources

In addition to the database searches, we used the following search strategies:

- Searching government and non‐government websites related to radicalisation, violent extremism and terrorism research for research related to courts and corrections (see Table 1)
- Searching government and non‐government websites related to courts and corrections for research related to radicalisation, violent extremism and terrorism interventions (see Table 1)
- Reference harvesting eligible documents
- Forward citation searching eligible documents using Paperfetcher (Pallath & Zhang, 2022)
- Contacting identified experts in the field to identify any potentially eligible studies that are not yet published.

These searches were conducted between 6 June and 13 June 2022. See the full search record for these sources in Supporting Information: Appendix 4.

### 4.5 Analysis and presentation

#### 4.5.1 Filters for presentation

The results of our online interactive EGM are presented as a matrix of rows (comprising two layers: (1) intervention agency; (2) intervention type) and columns (comrpising two layers: (1) broad and (2) specific outcomes). We additionally employed the following filters:

- Research design: RCT, strong quasi‐experimental, systematic review/meta‐analysis
- Document type: published, unpublished
- Study location: country, region
- Country income group: low income, middle income, high income
- Implementation setting: prisons/correctional facilities, courts, community, school, workplace, places of worship, home, other
- The focus of intervention: preventing radicalisation, preventing terrorism, preventing both
- Prevention category: primary, secondary, tertiary
- Age of targets: youth, adults, both
- Target population:
  ○ Criminal justice practitioners
  ○ Victims
  ○ Communities
  ○ Individuals or groups who have been identified as at risk of becoming radicalised or engaging in violent extremism and/or terrorist activity
  ○ Radicalised individuals or groups (including pre‐criminal justice involved radicalised individuals)
  ○ Individuals or groups who have engaged in violent extremism and/or terrorist activity
  ○ Family members of radicalised individuals or individuals who have engaged in violent extremism and/or terrorist activity
  ○ Micro places (e.g., street corners, buildings, police beats, street segments)
  ○ Macro places (neighbourhoods or larger geographies).

#### 4.5.2 Dependency

The unit of analysis is the primary study, not the publication or document that reports on the study. We are conscious of the risk of using documents as the unit of analysis, as a visual tool such as an EGM can appear to indicate a preponderance of evidence on an intervention where there are multiple publications drawn from the same research study.

The two most common ways in which dependency can manifest in an EGM are as many:one dependency or as one:many dependency. First, there may be more than one document reporting on the same study (many: one dependency). The EGM identified one study that was reported in two different documents. In this situation, both documents that reported on the one study were linked, and the study is what appears on the map and in the report. The second form of dependency that we identified was that one document may report on more than one study (one: many dependency), and we proposed in our protocol that if a document reports on more than one study, we would link that document to both ‘parent’ studies. This second form of dependency was not identified in the EGM.

Systematic reviews provide a unique challenge in terms of the one:many dependency. A systematic review may contain multiple studies, that may report on multiple interventions and outcome combinations. We included systematic reviews in addition to their component studies, but as only two systematic reviews were identified, we deviated from the protocol and presented both systematic reviews and primary studies on the same map. However, it should be noted that there is limited overlap between the studies included in the systematic reviews and the primary studies on the map. This is due to both systematic reviews uncovering a very small number of eligible studies that meet our study criteria.

In the protocol we identified the further issue of the potential for double counting when a study might appear in multiple cells of the map, and stated that these instances would be flagged. However, as this was such a common occurrence in the literature, the flagging approach was not practical. Instead of focusing on the potential for a visual inflation of the evidence base, we instead focus on demonstrating *where* evidence is available, and that we caution users that one study can appear in multiple intervention/outcome cells. Therefore, the number of studies that appear on the map in total ‐ the sum of all studies in all visible cells ‐ will necessarily change depending on the granularity of the map view i.e. higher level views will show a smaller number of units than lower level views.

### 4.6 Data collection and analysis

#### 4.6.1 Screening and study selection

Our screening approach differed slightly for the different sources, but all documents were assessed on the same final eligibility criteria. Documents from the GPD had already been screened on their full‐text as reporting on a quantitative impact evaluation of a policing intervention. Therefore, we assessed the full‐text of these documents for their eligibility for the review. Documents from other sources had not been verified as quantitative impact evaluations, so these were first screened on their title and abstract to remove documents not related to criminal justice responses to preventing radicalisation/violent extremism/terrorism, and then the full text documents were screened on the same criteria as the GPD data.

For all stages described below, we developed standardised screening companions and inter‐rater reliability tests to ensure consistency in decision‐making across the research team. For title and abstract screening, each team member screened the same set of 30 records before commencing screening. Similarly, at the full‐text screening stages, before commencing screening, team members screened the same set of 30 documents. All team member decisions were compiled and compared to determine any divergence in decisions. Feedback on screening decisions was provided to the research team members, with further training provided when necessary. We double‐screened 5% of each screener's excluded documents to identify any potential false‐negative decisions. In instances where a screener's decisions were deemed unreliable, their exclusion screenings were reassigned. Where disagreements about screening decisions occurred, these were mediated by a third team member.

All documents were screened in DistillerSR—a web based, reference management software that incorporates artificial intelligence (AI) to expedite the review process (Evidence Partners, 2022). DistillerSR has an inbuilt AI function that cross‐checks exclusion decisions. This helps to ensure consistency across reviewers and reduce human error. DistillerSR also draws on machine learning to reorder references based on their likelihood of inclusion, learning from the decisions made by human users (Evidence Partners, 2022). Using this function, we screened documents up until the point that DistillerSR indicated 95% of all potentially eligible records were included. At this point, we screened random samples of 30 additional records until no eligible records were identified. We used the AI function for Title and Abstract screening only. All documents that proceeded to full text screening stages were processed.

Following Campbell Collaboration guidelines (White et al., 2020), we screened systematic reviews on their PICOS rather than on their included studies. In this way, we retained ‘empty reviews’, and allowed the EGM user to see where synthesis had been attempted but was unsuccessful.

##### GPD sourced documents

First, we imported all documents reporting on a quantitative impact evaluation of a policing intervention captured by the systematic search into DistillerSR. Quantitative impact evaluations indexed in the GPD generally had a pre‐existing full‐text document attached, and therefore document retrieval was not required. We then screened the full‐text of the records according to the following exclusion criteria:

1. Document is not unique
2. Document is not about a criminal justice intervention for preventing radicalisation/violent extremism/terrorism
3. Document does not include an impact evaluation of a criminal justice intervention that aims to prevent radicalisation/violent extremism/terrorism.

While all effort was made to remove duplicate records within the GPD, exclusion criterion 1 was used to remove any that were missed during the initial data cleaning. Exclusion criterion 2 was used to remove records unrelated to radicalisation, extremism or terrorism. Exclusion criterion 3 was used to produce a corpus of studies that reported on quantitative impact evaluations of an eligible intervention, using an eligible research design. Exclusion criterion 3 deviated from the exclusion criteria outlined in the protocol (Sydes et al., 2022). The original exclusion criteria stated that the ‘document does not include an impact evaluation of a criminal justice intervention that aims to prevent or respond to radicalisation/violent extremism/terrorism.’ We removed the word ‘respond’ to ensure the included studies aligned with the overarching goals of the EGM.

As part of the GPD protocol, all attempts were made to locate full‐text documents via existing university resources, by ordering documents through the university library, or by directly contacting study authors. If the search within the GPD returned records (i.e., citations) for which we were unable to locate the full‐text and could not unequivocally exclude on the title and abstract as not relating to a criminal justice intervention for preventing to radicalisation/terrorism, the document was recorded as ‘Studies awaiting classification’.

##### Documents from other sources

Second, we imported all records identified by our electronic searches and other sources (grey literature, hand‐searching, etc.) into DistillerSR. This data was assessed on title and abstract on the following criteria:

1. Document is not unique
2. Ineligible document type
3. Document is not about a criminal justice intervention for preventing radicalisation/violent extremism/terrorism

Before screening, we made all effort to remove duplicates and ineligible document types (such as book reviews, blog posts etc). However, criteria 1 and 2 allowed us to remove any that were missed during data cleaning. Exclusion criterion 3 was used to remove titles and abstracts unrelated to terrorism, radicalisation or extremism (as above, we made a slight amendment to the wording of exclusion criterion 3, removing the word ‘respond’).

Records that successfully progress through the title and abstract screening stage were moved to a literature retrieval stage. For records without documents, we endeavoured to locate the full‐text document via existing university resources. In instances where we could not locate the record, we ordered the document through the university library or by directly contacting the study authors. Documents for which we could not locate the full‐text and could not unequivocally exclude on title and abstract are listed in ‘Studies awaiting classification’.

Once the literature was retrieved, all potentially eligible records were then progressed to full‐text eligibility screening. The full‐text of the records was assessed according to the following exclusion criteria:

1. Document is not unique
2. Ineligible document type
3. Document does not report on quantitative bivariate or multivariate data which may be indicative of an evaluation
4. Document is not about a criminal justice intervention for preventing radicalisation/violent extremism/terrorism
5. Document does not include an impact evaluation of a criminal justice intervention that aims to prevent radicalisation/violent extremism/terrorism

The addition of criterion 3 allowed us to refine the corpus of studies to those that do contain bivariate or multivariate data and may therefore be indicative of an impact evaluation. A similar approach was taken for the GPD (Higginson et al., 2015). Criteria 4 and 5 were used to remove records unrelated to terrorism, radicalisation or extremism and produce a corpus of studies that reported on quantitative impact evaluations of an eligible intervention, using an eligible research design.

#### 4.6.2 Data extraction and management

Once deemed eligible for the review, documents were coded and entered into EPPI‐reviewer. We used a standardised coding companion (see Supporting Information: Appendix 5) to code documents across the following domains:

1. Study Characteristics (study location, research design, etc.)
2. Intervention (setting, components, agencies, etc.)
3. Outcomes (conceptualisation, data source)

All coders coded the same four eligible studies to ensure consistency across team members. These results were compiled and compared to determine any divergence in coding, and feedback was provided before independent coding. To check for inter‐rater reliability, 5% of each person's coding was double‐coded by a second person. Sufficient information was included in the eligible documents, therefore, we did not contact study authors. Systematic reviews were coded based on their PICOS, rather than on their individual studies.

#### 4.6.3 Tools for assessing risk of bias/study quality of included studies

Each eligible systematic review included in the EGM was assessed for Risk of Bias using the AMSTAR 2 critical appraisal tool (Shea et al., 2017). Based on ratings across 16 items, the tool generated a rating of overall confidence in the systematic review's results: High (no more than one non‐critical weakness, but no critical flaws); Moderate (more than one non‐critical weakness, but no critical flaws); Low (one critical flaw, with or without non‐critical weaknesses); or Critically Low (more than one critical flaw, with or without non‐critical weaknesses). Two coders independently coded each systematic review using the AMSTAR 2 tool. Any discrepancies between the two coders were resolved by discussion. Due to the size of the EGM, we did not assess confidence in the included primary studies, however, our methodological thresholds excluded study designs with the highest inherent risk of bias.

#### 4.6.4 Methods for mapping

We used EPPI‐reviewer to create the EGM (Eppi‐Centre, 2019).

## 5 RESULTS

### 5.1 Description of studies

#### 5.1.1 Results of the search

**Figure 1:**
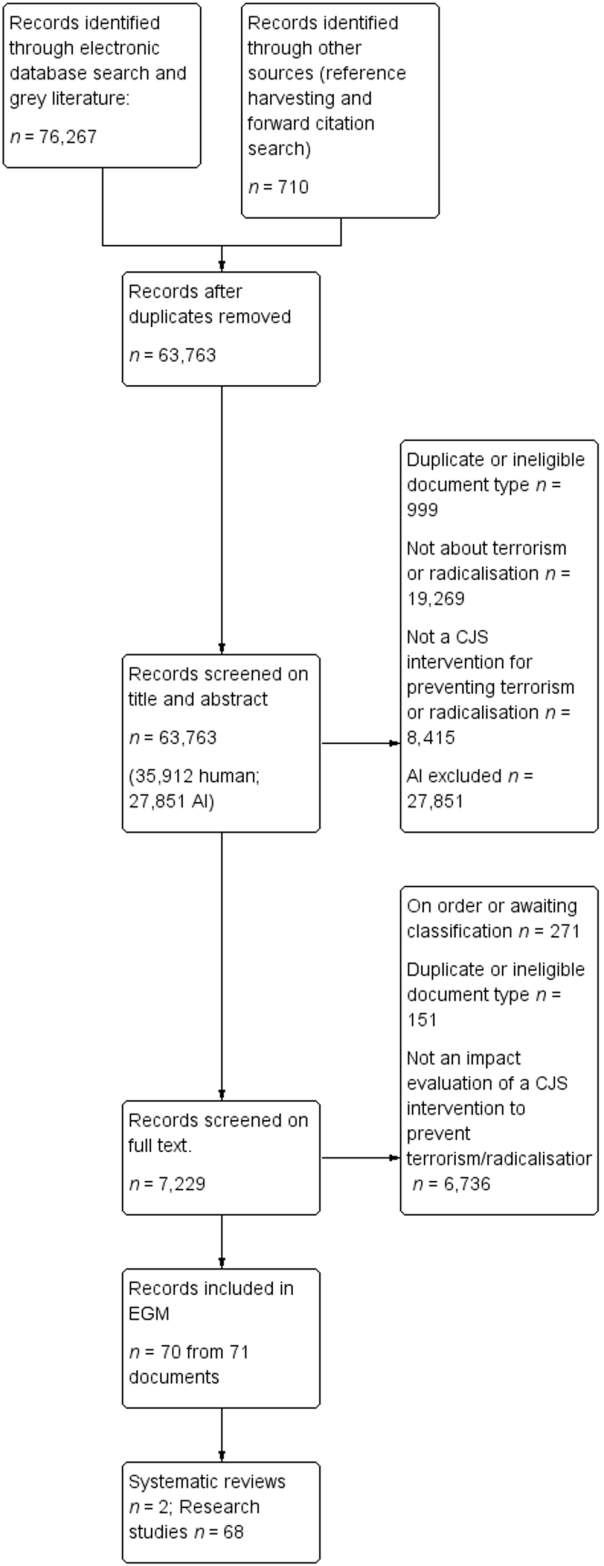
PRISMA flow diagram.

The results of the search and screening are outlined in Figure 1.

Our systematic search identified 76,977 records between January 2002 and December 2021 (GPD = 815; electronic database search = 54,567; grey literature = 20,885, hand search, forward citation and reference harvesting = 710). Before uploading records into DistillerSR, we carried out data cleaning in EndNote. As part of this process, we removed duplicates, ineligible document types and studies outside of the data range (*n* = 10,748 records). A total of 66,229 records were imported into DistillerSR for eligibility screening.

Once records were uploaded into DistillerSR, we utilised the duplicate detection function to identify any potential duplicate records. We first used the automated tool, quarantining records that were identified as a 95% match. We then proceeded to manually check the remaining records identified as potential duplicates. A total of 2466 duplicates were found through this process. *N* = 63,763 records were screened on title and abstract.

In all, 35,912 records were screened manually during the title and abstract screening (approximately 56.35% of records captured in our search). At this point, the AI prioritisation function tool indicated that over 95% of potentially relevant references were screened. We then randomly screened batches of 30 records until no potentially eligible studies were identified. All remaining records (*n* = 27,851) were excluded.

Of the 7,229 records eligible on title and abstract, 267 could not be located or are currently on order using university library resources. An additional four records could not be processed through Google Translate and thus a final eligibility decision could not be made (these studies are listed under studies awaiting classification). A total 151 duplicates and ineligible document types were identified at full‐text screening. A further 6,736 were excluded as they did not contain an impact evaluation of an eligible intervention. Most of these studies were excluded for not containing quantitative data. Of the full‐text documents reviewed, 70 were deemed eligible (two systematic reviews; 68 primary research studies from 71 documents).

#### 5.1.2 Excluded studies

Given the large volume of studies that proceeded to full‐text screening (*n* = 7,229), we are not able to provide an exhaustive list of all excluded studies that proceeded to full text screening or justify reasons for their exclusion. However, as stated above, the large majority did not include bivariate or multivariate data which was difficult to ascertain at the title and abstract stage. Instead, our excluded studies list includes *almost* eligible studies (*n* = 329). These were studies that were screened as having quantitative data and were focused on radicalisation/terrorism. These studies were excluded because they did not contain an eligible intervention (*n* = 124) or an eligible study design (*n* = 209). A notable exclusion from our included studies list is a systematic review carried out by Lum et al. (2006a). While this review meets the EGM eligibility criteria based on its PICOS, it was excluded as it did not contain any studies published within our date range. Please refer to the excluded studies list for further information.

### 5.2 Synthesis of included studies

#### 5.2.1 Intervention

**Figure 2:**
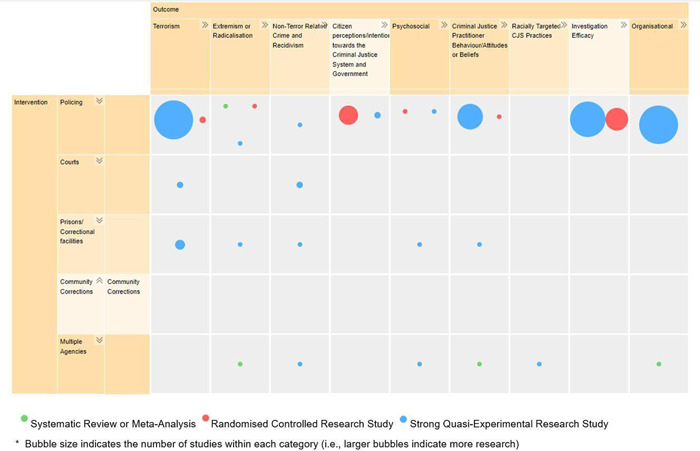
The evidence and gap map interface for Criminal justice interventions for preventing terrorism and radicalisation.

Interventions on the EGM are displayed on two levels, with users able to expand and collapse intervention and outcome categories when using the online interactive map. Interventions were first grouped by criminal justice branch (police, courts, custodial corrections, community corrections and multi‐agency involving at least one criminal justice partner, see Figure 2). Most studies were classified as straight policing interventions (*n* = 58) Further, while four studies reported on multi‐agency partnerships, three were police partnerships with the United States Department of Homeland Security. For the remaining studies, three were prison interventions and another three were courts interventions. One study was classified as containing both a police and courts intervention (White et al., 2014) and another was coded as reporting on both a police and corrections intervention (Asal, Rethemeyer, & Young, 2016). In these cases, a single study will appear across multiple cells in the map (see dependency for further explanation).

Our 70 eligible studies displayed on the map report on a broad range of criminal justice interventions. Through our thematic analysis, we identified 29 different policing interventions, three courts interventions, five custodial corrections interventions and four multi‐agency interventions with criminal justice partners (see Table 2). No eligible interventions were found for community‐based corrections. See Table 2 for a clear breakdown of the types of interventions included.

**Table 2:** Intervention categorisation.

Broad intervention category	Specific intervention category
Police (*n* = 29)	Arrests of suspected terrorists Call centres, tip lines and information centres Community connectedness Community engagement Community policing practices Counterterrorism officers Counterterrorism training for police Deradicalisation program Expanded police powers Homeland Security Grants or Funding Information or advice disseminated to the wider public Information sharing initiative Intelligence‐led policing Inter‐agency collaboration Interrogation and interviewing techniques Investigation guidelines Involvement with a Joint Task Force Monitoring or surveillance Police crackdowns Police millitarisation Police presence Preparedness for terrorism incidents Procedurally just practices Protection of individual targets Punitive/punishment/stick approaches Tactical or investigatory equipment Training for detecting suspicious activities or behaviours Use of lethal force Vulnerability assessments
Courts (*n* = 3)	Criminal charges filed Punitive/punishment approaches Terrorism specific charges
Custodial Corrections (*n* = 5)	Deradicalisation program Imprisonment Isolation of radicalised inmates Monitoring of radicalised inmates Training for corrections staff relating to managing extremist inmates
Community Corrections (*n* = 0)	*None captured*
Multi‐agency with at least one criminal justice partner (*n* = 4)	Community engagement Information sharing initiative Inter‐agency collaboration Warning or alert scales

*Note*: Italicisation indicates gaps in research.

#### 5.2.2 Outcomes

Users of the interactive EGM can view the map outcomes on either a broad or specific level. Specific outcome categories were developed based on the corpus of eligible studies (see Table 3). In total, our 70 eligible studies utilised 50 different outcomes to assess intervention effectiveness. To demonstrate areas where gaps exist, we included three additional outcome measures (injuries caused by terrorism, terrorism‐related recidivism and non‐terror‐related recidivism). These outcomes were not featured in our eligible studies but could reasonably be employed in evaluating the effectiveness of criminal justice interventions to prevent radicalisation/violent extremism/terrorism. These ‘empty’ outcomes are italicised in Table 3 and included on the EGM.

**Table 3:** Outcome categorisation.

Broad category	Specific category
Terrorism (*n* = 20)	1. Terrorism incident 2. *Recidivism*—*terrorism related offence* 3. Citizen willingness to report terrorism 4. Diffusion/displacement of terrorism 5. Fatalities due to terrorism 6. *Injuries resulting from terrorism*
Extremism or radicalisation (*n* = 5)	1. Risk assessment tools for high‐risk individuals 2. Deradicalisation 3. Perceptions, attitudes or opinions regarding the legitimacy of, or support for the plight of terrorists 4. Support for extremist movement or ideology 5. Engagement in extremist activity 6. Willingness to engage in violence 7. Extremist social networks
Non‐terror related crime and recidivism (*n* = 5)	1. Non terror related crime 2. *Non terror related recidivism* 3. Sentence length
Citizen perceptions/intentions toward the criminal justice system and government (n = 8)	1. Attitudes regarding the criminal justice system 2. Attitudes toward government or country 3. Perceptions of criminal justice personnel or activities 4. Perceptions of criminal justice legitimacy, distributive justice, procedural justice 5. Willingness to obey the law/cooperate with the police 6. Willingness to follow police advice 7. Disclosure of information pertaining to terrorism activities 8. Willingness to engage in protective and risky behaviours
Psychosocial (*n* = 4)	1. Fear of terrorism 2. Community/social connectedness 3. Feelings of wellbeing 4. Attitudes toward/perceptions of other groups/out group members 5. Consumer behaviour
Criminal justice practitioner behaviours/attitudes or beliefs (*n* = 11)	1. Knowledge or expertise regarding terrorism and/or radicalisation to violence 2. Adherence to intervention implementation 3. Community policing activities 4. Perceptions of intervention effectiveness 5. Perceptions of terrorism or radicalisation risk 6. Perceptions of organisational preparedness 7. Perceptions of organisational efficacy 8. Perceptions of organisational priorities
Racially targeted criminal justice practices (*n* = 1)	1. Arrests 2. Frisks 3. Police stops 4. Police use of force
Investigation efficacy (*n* = 18)	1. Detection of guilt or deception 2. Detection of suspicious activities or behaviour 3. Information gathering 4. Analytic and/or intelligence outputs
Organisational (*n* = 16)	1. Dissemination of resources, techniques, guidance, or approaches 2. Funding, grants, or financial support for operations 3. Information sharing 4. Inter‐agency collaboration 5. Staffing or employment of terrorism‐specific personnel 6. Training for personnel 7. Uptake or adoption of Homeland Security Initiatives 8. Organisational structure, hierarchy or complexity

*Note*: Italicisation indicates gaps in research. Note* this is a count of total outcomes measures, some studies measured multiple outcomes.

From the specific outcome groups, nine broad overarching themes emerged. These include: (1) terrorism; (2) extremism or radicalisation; (3) non‐terror related crime and recidivism; (4) citizen perceptions/intentions toward the criminal justice system and government; (5) psychosocial factors; (6) criminal justice practitioner behaviours/attitudes/beliefs; (7) racially targeted criminal justice practices; (8) investigation efficacy and; (9) organisational factors. Similar to the interventions, many studies report multiple outcome measures and thus may appear across multiple cells in the map. Table 3 provides a breakdown of the number of studies within each broad intervention category.

The most frequently assessed outcome category was terrorism with 15 policing interventions (two RCTs, 15 quasi‐experiments), two courts interventions (both quasi‐experiments) and three corrections interventions (all quasi‐experiments) employing terrorism related outcome measures such as terrorism incidents. Interventions that were evaluated based on investigation efficacy (seven RCTs, 11 quasi‐experiments) and/or organisational factors (15 quasi‐experiments, one systematic review) were also common, particularly when evaluating policing interventions. In contrast, relatively few interventions employed measures of radicalisation or extremism in evaluating impact.

#### 5.2.3 Study design and document type

**Figure 3:**
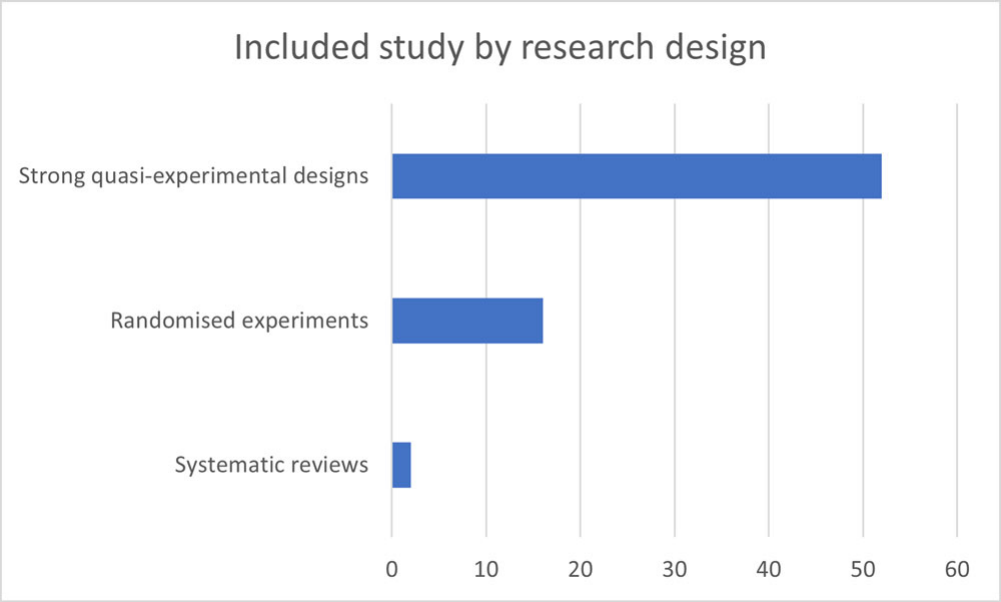
Included studies by research design.

As can be seen in Figure 3, most of the included studies (74.29%) were classified as a strong quasi‐experimental design (*n* = 52). Our EGM contains just 16 randomised experiments and two systematic reviews. Table 4 provides a breakdown of the included studies by document type. Journal articles comprised the majority of eligible studies (*n* = 46) followed by theses/dissertations (*n* = 15). Books, book chapters, conference papers and reports were less common.

**Table 4:** Included studies by document type.

Document type	Frequency	Percentage
Book	1	1.43
Book chapter	1	1.43
Conference paper	2	2.86
Journal article	46	65.71
Report/other	5	7.14
Thesis or dissertation	15	21.43
Total	70	100.00

#### 5.2.4 Study location

**Figure 4:**
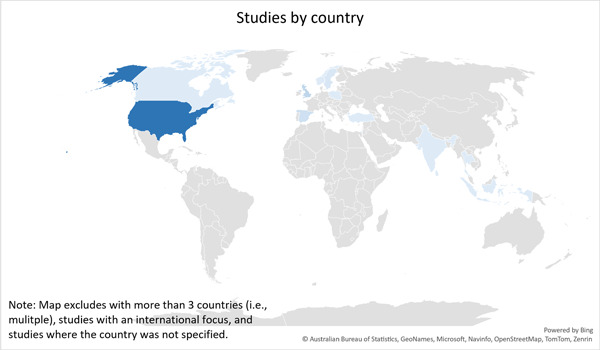
Studies by country location.

Table 5 and Figure 4 offer an overview of the included studies by study region. Just over half of the studies included in the EGM were situated in North America (*n* = 36), followed by North‐West Europe (*n* = 10) and Southern and Eastern Europe (*n* = 5). Very limited evidence was found in North Africa and the Middle East (*n* = 2), Southern and Central Asia (*n* = 2) and South East Asia (*n* = 1). No evidence was found for Central or South America, Sub‐Saharan Africa, North East Asia or Oceania. It should be noted however that the lack of evidence in these regions could be a function of the English search terms employed. Over three quarters of the research was carried out in high‐income countries (as classified by the World Bank, 2021).

**Table 5:** Included studies by study region.

Study region	Frequency	Percentage
North America	36	51.43
Central or South America	0	0.00
Oceania	0	0.00
North‐West Europe	10	14.29
Southern and Eastern Europe	5	7.14
North Africa and the Middle East	2	2.86
Sub‐Saharan Africa	0	0.00
South‐East Asia	1	1.43
North‐East Asia	0	0.00
Southern and Central Asia	2	2.86
Other (multiple countries/international focus/unspecified)	14	20.00
Total	70	100.00

#### 5.2.5 Intervention focus and ideology targeted

Most studies contained interventions focused on preventing terrorism (*n* = 54) rather than radicalisation (*n* = 4). Twelve studies were categorised as preventing both radicalisation and terrorism. The majority of interventions evaluated did not explicitly target one particular ideology (*n* = 62) (Table 6). From the remaining studies, four studies targeted far‐left ideologies (mostly Basque nationalism), three targeted far‐right ideologies and one targeted eco‐terrorism. We found no studies that explicitly targeted ‘lone wolf’ attacks.

**Table 6:** Included studies by ideology targeted.

Ideology	Frequency	Percentage
Far right	3	4.29
Far left	4	5.71
Single issue: Eco‐terrorism	1	1.43
Lone wolf	0	0.00
General/not specified	62	88.57
Total	70	100.00

#### 5.2.6 Target population

Almost a quarter of the interventions reported in the included studies (24.29%) were targeted toward agencies or organisations and a further 20.00% were targeted toward criminal justice practitioners (see Table 7). This is not surprising given agencies and practitioners are of more accessible target population than at risk groups. Another 31.43% studies reported on interventions that were designed to target either at risk or radicalised individuals. Other studies were focused on micro, meso and macro places (2, 8 and 5, respectively). No studies reported on interventions targeting victims or family members of radicalised individuals, highlighting in a clear gap in the evaluation research.

**Table 7:** Included studies by target population.

Target population	Frequency	Percentage
Individuals or groups who have been identified as at risk of becoming radicalised or engaging in violent extremism	2	2.86
Individuals or groups who have been engaged in violent extremism and/or terrorist activity, or are suspected to have engaged in these activities	18	25.71
Radicalised individuals or groups	2	2.86
Agencies or organisations	17	24.29
Criminal justice practitioners	14	20.00
Micro places (e.g., street corners, buildings, police beats, street segments)	2	2.86
Communities	8	11.43
Macro places (neighbourhoods or larger geographies)	5	7.14
Victims	0	0.00
Family members of radicalised individuals or individuals who have engaged in violent extremism and/or terrorist activity	0	0.00
Multiple populations	2	2.86
Total	70	100.00

### 5.3 Risk of bias in included reviews

Our EGM includes two Campbell Collaboration systematic reviews (Mazerolle et al., 2020, 2021). Risk of bias for each of these reviews was assessed using the AMSTAR 2 Checklist (Shea et al., 2017). Both reviews were assessed as ‘moderate’ quality (see Table 8). For Mazerolle et al. (2020), 12 checklist items were marked as yes, three items were marked as ‘no meta‐analysis conducted’, and one item was marked as ‘partial yes’. Specifically, checklist item 7 (related to excluded studies) was marked as partial yes. This is because the authors provided a list of excluded studies but did not provide justifications for all exclusions. For Mazerolle et al. (2021), 12 checklist items were marked as ‘yes’, 3 items were marked as ‘no meta‐analysis conducted,’ and 1 item was marked as ‘no’. Checklist item 7 (excluded studies) was rated as ‘no’. The authors noted that due to the large volume of full‐text studies screened (*n* = 5,149), it was not possible to provide an exhaustive list of excluded studies or justify exclusion decisions.

**Table 8:** Risk of bias.

AMSTAR 2 checklist	Mazerolle et al. (2020)	Mazerolle et al. (2021)
1. Did the research questions and inclusion criteria for the review include the components of PICO?	Yes	Yes
*For Yes*:		
Population Intervention Comparator Group Outcome Timeframe for follow‐up (optional)		
2. Did the report of the review contain an explicit statement that the review methods were established prior to the conduct of the review and did the report justify any significant deviations from the protocol?	Yes	Yes
*For Partial Yes*:		
Review question(s) A search strategy Inclusion/exclusion criteria A risk of bias assessment		
For Yes:		
A meta‐analysis/synthesis plan, if appropriate a plan for investigating causes of heterogeneity		
3. Did the review authors explain their selection of the study designs for inclusion in the review?	Yes (explanation for including both RCTs and NRSI)	Yes (explanation for including both RCTs and NRSI)
*For Yes, the review should satisfy ONE of the following*:		
Explanation for including only RCTs OR explanation for including only NRSI OR explanation for including both RCTs and NRSI		
4. Did the review authors use a comprehensive literature search strategy?	Yes	Yes
*For Partial Yes (all the following)*:		
Searched at least 2 databases Provided key word and/or search strategy Justified publication restrictions (e.g., language)		
*For Yes, should also have (all the following)*:		
Searched the reference lists/bibliographies of included studies Searched trial/study registries Included/consulted content experts in the field Where relevant, searched for grey literature Conducted search within 24 months of completion of the review.		
5. Did the review authors perform study selection in duplicate?	Yes (two reviewers selected a sample of eligible studies and achieved good agreement (at least 80%), with the remainder selected by one reviewer)	Yes (two reviewers selected a sample of eligible studies and achieved good agreement (at least 80%), with the remainder selected by one reviewer)
*For Yes, either ONE of the following*:		
At least two reviewers independently agreed on selection of eligible studies and achieved consensus on which studies to include OR two reviewers selected a sample of eligible studies and achieved good agreement (at least 80%), with the remainder selected by one reviewer		
6. Did the review authors perform data extraction in duplicate?	Yes (two reviewers extracted data from a sample of eligible studies and achieved good agreement (at least 80%), with the remainder extracted by one reviewer)	Yes (two reviewers extracted data from a sample of eligible studies and achieved good agreement (at least 80%), with the remainder extracted by one reviewer)
*For Yes, either ONE of the following*:		
At least two reviewers achieved consensus on which data to extract from included studies OR two reviewers extracted data from a sample of eligible studies and achieved good agreement (at least 80%), with the remainder extracted by one reviewer		
7. Did the review authors provide a list of excluded studies and justify the exclusions?	Partial Yes (provided a list of all potentially relevant studies that were read in full‐text form but excluded from the review)	No (the authors noted it was not possible to include a list of all full‐text studies examined given the large number of studies reviewed).
*For Partial Yes*:		
Provided a list of all potentially relevant studies that were read in full‐text form but excluded from the review		
*For Yes, must also have*:		
Justified the exclusion from the review of each potentially relevant study		
8. Did the review authors describe the included studies in adequate detail?	Yes	Yes
*For Partial Yes*:		
described populations described interventions described comparators described outcomes described research designs		
*For Yes, should also have ALL of the following*:		
described population in detail described intervention in detail (included doses where relevant) described comparator in detail (including doses where relevant) described study's setting timeframe for follow‐up		
9. Did the review authors use a satisfactory technique for assessing the risk of bias (RoB) in individual studies that were included in the review?	RCTs—N/A No RCTs were included. NRSI—Yes	RCTs—N/A No RCTs were included. NRSI—Yes
*RCTs*		
*For Partial Yes, must have assessed RoB from*:		
Unconcealed allocation, and Lack of blinding of patients and assessors when assessing outcomes (unnecessary for objective outcomes such as all cause mortality)		
*For Yes, must also have assessed RoB from*:		
Allocation sequence that was not truly random, and Selection of the reported result from among multiple measurements or analyses of a specified outcome		
*NRSI*		
*For Partial Yes, must have assessed RoB*:		
From confounding, and From selection bias		
*For Yes, must also have assessed RoB*:		
Methods used to ascertain exposures and outcomes, and Selection of the reported result from among multiple measurements or analyses of a specified outcome		
10. Did the review authors report on the sources of funding for the studies included in the review?	Yes	Yes
*For Yes*		
Must have reported on the sources of funding for individual studies included in the review. Note: Reporting that the reviewers looked for this information but it was not reported by study authors also qualifies.		
11. If meta‐analysis was performed did the review authors use appropriate methods for statistical combination of results?	No meta‐analysis conducted	No meta‐analysis conducted
*RCTS*		
The authors justified combining the data in a meta‐analysis AND they used an appropriate weighted technique to combine study results and adjusted for heterogeneity if present. AND investigated the causes of any heterogeneity		
*For NRSI*		
The authors justified combining the data in a meta‐analysis AND they used an appropriate weighted technique to combine study results, adjusting for heterogeneity if present AND they statistically combined effect estimates from NRSI that were adjusted for confounding, rather than combining raw data, or justified combining raw data when adjusted effect estimates were not available AND they reported separate summary estimates for RCTs and NRSI separately when both were included in the review.		
12. If meta‐analysis was performed, did the review authors assess the potential impact of RoB in individual studies on the results of the meta‐analysis or other evidence synthesis?	No meta‐analysis conducted	No meta‐analysis conducted
Included only low risk of bias RCTs OR, if the pooled estimate was based on RCTs and/or NRSI at variable RoB, the authors performed analyses to investigate possible impact of RoB on summary estimates of effect.		
13. Did the review authors account for RoB in individual studies when interpreting/discussing the results of the review?	Yes (if RCTs with moderate or high RoB, or NRSI were included in the review provided a discussion of the likely impact of RoB on the results)	Yes (if RCTs with moderate or high RoB, or NRSI were included in the review provided a discussion of the likely impact of RoB on the results)
Included only low risk of bias RCTs OR, if RCTs with moderate or high RoB, or NRSI were included in the review provided a discussion of the likely impact of RoB on the results.		
14. Did the review authors provide a satisfactory explanation for, and discussion of, any heterogeneity observed in the results of the review? There was no significant heterogeneity in the results OR if heterogeneity was present the authors performed an investigation of sources of any heterogeneity in the results and discussed the impact of this on the results of the review	Yes (there was no significant heterogeneity in the results)	Yes (there was no significant heterogeneity in the results)
15. If they performed quantitative synthesis did the review authors carry out an adequate investigation of publication bias (small study bias) and discuss its likely impact on the results of the review? Performed graphical or statistical tests for publication bias and discussed the likelihood and magnitude of impact of publication bias	No meta‐analysis conducted	No meta‐analysis conducted
16. Did the review authors report any potential sources of conflict of interest, including any funding they received for conducting the review? The authors reported no competing interests OR the authors described their funding sources and how they managed potential conflicts of interest	Yes (the authors described their funding sources and how they managed potential conflicts of interest)	Yes (the authors described their funding sources and how they managed potential conflicts of interest)
Overall rating	Moderate	Moderate

## 6 DISCUSSION

### 6.1 Summary of main results

Our EGM captures 70 eligible studies from 71 documents. This includes two systematic reviews, 16 randomised experiments and 52 strong quasi‐experimental designs. The evaluation evidence is unevenly distributed across interventions with the overwhelming majority reporting on policing interventions targeting terrorism. Limited studies evaluate courts or custodial corrections interventions. No studies evaluated community corrections interventions. While a considerable number of studies (*n* = 20) use measures related to terrorism to evaluate intervention effectiveness (such as number of terrorism incidents, fatalities resulting from terrorism, and citizen willingness to report terrorism), very few studies evaluate intervention effectiveness using measures of radicalisation and/or extremism (such as risk assessment tools, deradicalisation, support for terrorism, engagement in extremist activity and connection to extremist social networks).

### 6.2 Areas of major gaps in the evidence

Several evidence gaps are worth noting. First, eligible studies tended to focus on terrorism related outcomes rather than extremism or radicalisation related outcomes. Evaluating effective ways criminal justice practitioners could intervene to assist the disengagement/deradicalisation process is a key area for future research. Second, our review found no eligible studies examining extremist related recidivism outcomes, likely due to the lack of community corrections studies identified. Last, despite the opportunities available to intervene in online settings, we found no internet‐based criminal justice interventions. We suggest that this area requires further exploration.

However it is important to note that gaps on the EGM are representative of a lack of strong impact evaluation evidence, rather than necessarily a lack of interventions. This distinction is particularly important for corrections—where we find limited evaluation research related to prisons and no evaluation research pertaining to community‐based approaches. However, we are aware of several prison‐based interventions designed to prevent/reduce radicalisation. For instance, Vejvodová & Kolář (2020) details training initiatives and awareness programs with corrective services staff to assist in the detection and prevention of both radicalisation and violent extremism and van der Heide and Kearney (2020) describe specialised terrorist units used to house extremist offenders in prison. Others discuss targeted rehabilitation and reintegration programs delivered by corrections agencies. For instance, Cherney and Belton (2021) examine the Proactive Integrated Support Model (PRISM), a disengagement intervention delivered to radicalised or at‐risk inmates. However, methodological challenges such as small participant numbers, and difficulties accessing often sensitive data present an ongoing challenge for carrying out impact evaluations in this space (Cherney & Belton, 2021).

### 6.3 Limitations of the EGM

We employed a comprehensive search strategy covering the GPD, 8 academic database platforms, 5 trial registries and over 30 grey literature sources to inform this EGM. However, there are several limitations which should be acknowledged.

1. While we included general and broad search terms related to terrorism, we did not include specific terms targeting eco‐, bio‐ or cyber‐terrorism. While these studies were included within our definition of terrorism (following a deviation from the protocol), we may not have captured all eligible studies related to eco, cyber or bio‐terrorism attacks.
2. Although we did not place restrictions on language, our search terms were in English only. Therefore, studies written in languages other than English may not have been identified in our search. We recommend future updates of this EGM consider expanding the search terms to include languages other than English, particularly given the global nature of radicalisation, violent extremism and terrorism.
3. We searched an extensive list of grey literature locations (see Table 1) which was informed by previous systematic reviews in this area (Mazerolle et al., 2020, 2021) and expert advice. However, our list was not exhaustive and thus there is potential grey literature that was not located in the search. In particular, we recognise our grey literature search locations largely focus on research repositories based in the United States, Canada, the United Kingdom, Australia, and New Zealand. Future updates of this EGM could consider expanding the grey literature search locations to ensure greater representation of the Global South.

### 6.4 Stakeholder engagement throughout the EGM process

Given project time constraints we found stakeholder engagement challenging to incorporate throughout the EGM process. Stakeholder engagement remains ongoing and may inform future iterations of the map.

## 7 AUTHORS’ CONCLUSIONS

### 7.1 Implications for research, practice and/or policy

Our risk of bias assessment of the two eligible systematic reviews indicated that these reviews were of ‘moderate’ quality. In line with the Campbell Collaboration EGM guidelines, we did not carry out a risk of bias assessment for primary research studies. However, we restricted our inclusion criteria to high‐quality impact evaluations and thus our methodological thresholds excluded study designs with the highest inherent risk of bias.

The vast majority of the studies we found were policing interventions. The map also revealed a number of significant gaps in studies evaluating criminal justice responses to preventing radicalisation, violent extremism and terrorism. Most notably, we identified no studies in the area of community corrections, just three interventions implemented by the courts, and four custodial correctional programs. While outcomes related to terrorism (such as terrorism incidents, and fatalities caused by terrorism) were commonly used, very few studies assessed intervention effectiveness against measures of radicalisation/extremism.

Conducting high‐quality evaluation research on rare and hidden problems is a well‐known and challenging problem in criminological and criminal justice research (Curtis, 2013; Petersen & Valdez, 2005; Watters & Biernacki, 1989). Problems like terrorism and radicalisation fall into this category of rare (Bausch, Faria & Zeitzoff, 2013), hidden (see Merari, 1991; Silke, 2009) and hard‐to‐reach populations (see Silke, 2001). Moreover, the unique nature of some of the more challenging dynamics of terrorism—such as the secretive and hidden nature of radicalisation pathways (see McCauley & Moskalenko, 2008) and the complexities around terrorism target selection (see Clarke & Newman, 2006)—requires evaluators to think creatively about applying strong methodological approaches to assess intervention impacts. It is also possible that some counterterrorism evaluations are ‘unpublishable’ due to the sensitive nature of the data they draw upon.

Researcher‐practitioner partnerships with criminal justice agencies often take many years to ‘develop, grow and maintain’ (Rudes et al., 2014, p. 249). Made ever more complicated by the rarity and hidden nature of terrorism problems, skilled criminal justice evaluators are needed who can use a range of methodological and statistical approaches to generate high‐quality scientific results that offer high validity and reliability in the findings. Building these researcher‐practitioner terrorism evaluation partnerships requires mutual trust (see Rudes et al., 2014) and the need for evaluators to have an early seat at the planning table (see La Vigne, 2021). It is therefore unsurprising in our EGM that we find very few RCTs (*n* = 16) and a relatively high volume of rejected studies using ineligible study methods. We suggest, therefore, that future counterterrorism research pays attention to building researcher‐practitioner partnerships that focus on scientific quality with a clear agenda to build a more robust evidence base.

With the results of our EGM in mind, we challenge all areas of the criminal justice system to foster evaluation partnerships with skilled evaluators in an effort to build a more substantial evidence base on the effectiveness of counterterrorism programs, particularly in the domains of courts and corrections (both custodial and community‐based). We further suggest that future research focus attention on studies that consolidate sound measurement of terrorism‐related outcomes and, at the same time, expands the range of outcome measures to better capture the potential benefits and harms of counterterrorism programs.

## CONTRIBUTIONS OF AUTHORS

- Content: Michelle Sydes, Lorelei Hine, Laura Dugan, Angela Higginson, Lorraine Mazerolle.
- Information retrieval: Lorelei Hine, Michelle Sydes, Angela Higginson, James McEwan.
- EGM methods: James McEwan, Michelle Sydes, Angela Higginson

## DECLARATIONS OF INTEREST

Two of the review authors held internal roles within the Campbell Collaboration Crime and Justice Group while understaking this project. Angela Higginson is an Editor and Co‐Chair of the Crime and Justice Coordinating Group (CJCG) and Lorelei Hine was the Managing Editor of the CJCG. Angela Higginson and Lorelei Hine were not involved in any editorial or internal Campbell Collaboration communications about this review.

## PLANS FOR UPDATING THE EGM

Michelle Sydes will be responsible for updates of this review, which are anticipated to occur every 5 years.

## DIFFERENCES BETWEEN PROTOCOL AND REVIEW

We made several deviations from the protocol. These are outlined below:

- Defining terrorism:
  ○ We extended our initial definition of terrorism to cover both bioterrorism and cyberterrorism based on early screening patterns. This definition is provided in full under Types of Interventions.
- Systematic search:
  ○ We stated in the protocol that we would search journals categorised by Web of Science as related to terrorism and radicalisation. This was not necessary as these journals were adequately covered by our systematic search in Web of Science. We did however include additional terrorism journals that were not indexed in any academic databases (listed in Table 1).
- Screening criteria:
  ○ In the screening criteria provided in the protocol we referred to criminal justice interventions for preventing or *responding* to terrorism/radicalisation. Given the overall focus of the review is prevention, we found that including the word ‘respond’ did not align with the project aims and therefore was removed.
- Forward citation search:
  ○ In the protocol, we outlined a plan to use Google Scholar to carry out our forward citation search. To expedite this process, we used Paperfetcher (Pallath & Zhang, 2022). Paperfetcher uses the DOI to automatically retrieve all DOIs that have cited the research. Therefore research without a DOI that had cited the study would not have been located through this approach.
- Title and Abstract screening:
  ○ We initially planned to continue Title and Abstract screening up until the point that DisitllerSR indicated 100% of all potentially eligible records were included. Given the large volume of records captured in our systematic search and the broad scope of our review, we opted to screen Title and Abstracts until we reached 95% of all potentially eligible records. This approach is in line with Sarma (2022).
- Coding:
  ○ We used a simplified coding form than the form provided in the protocol. This was to ensure only information that would be included in the EGM was extracted during the coding process.
- Presentation of the EGM:
  ○ In the protocol we planned to map systematic reviews and research studies separately. However, there was an insufficient number of systematic reviews captured in our search (*n* = 2) to warrant two separate maps. Instead, we chose to map systematic reviews and research studies together. We used colour coding to differentiate between study types.
  ○ While we stated in the protocol that we planned to visually differentiate between systematic reviews that have included studies and those that do not, our study *only* captured systematic reviews that contained potentially eligible studies.
  ○ We also stated in the protocol that where studies appear in more than one column of the map, this would be flagged to avoid the potential for double counting. During the process of creating the map, it became clear that many studies included multiple eligible interventions and/or multiple eligible outcomes. The issue of presenting studies in multiple cells therefore became unavoidable—particularly when the map is shown at its finest granularity. To align more closely with user needs and expectations, we chose to focus on demonstrating *where* there were studies, and cautioned the user against double counting. We provide further detail under Dependencies.

## PUBLISHED NOTES

**Characteristics of studies**

**Characteristics of included studies**

**Table:** 

Adcox 2014	
**Notes**	
Risk of bias table	
Aksu 2014	
**Notes**	
Risk of bias table	
Alison et al. 2013	
**Notes**	
Risk of bias table	
Alison et al. 2014	
**Notes**	
Risk of bias table	
Amirault and Bouchard 2017	
**Notes**	
Risk of bias table	
Amirault et al. 2016	
**Notes**	
Risk of bias table	
Anarumo 2005	
**Notes**	
Risk of bias table	
Asal et al. 2016	
**Notes**	
Risk of bias table	
Barros et al. 2009	
**Notes**	
Risk of bias table	
Baudains et al. 2019	
**Notes**	
Risk of bias table	
Bentley 2020	
**Notes**	
Risk of bias table	
Buesa and Baumert 2018	
**Notes**	
Risk of bias table	
Burruss et al. 2012	
**Notes**	
Risk of bias table	
Campbell 2016	
**Notes**	
Risk of bias table	
Carter et al. 2014	
**Notes**	
Risk of bias table	
Chenoweth and Clarke 2010	
**Notes**	
Risk of bias table	
Christiansen et al. 2018	
**Notes**	
Risk of bias table	
Comens 2016	
**Notes**	
Risk of bias table	
Davis et al. 2004	
**Notes**	
Risk of bias table	
Davis et al. 2016	
**Notes**	
Risk of bias table	
Freilich et al. 2020	
**Notes**	
Risk of bias table	
Fritz 2019	
**Notes**	
Risk of bias table	
Giblin et al. 2009	
**Notes**	
Risk of bias table	
Granhag et al. 2015	
**Notes**	
Risk of bias table	
Granhag et al. 2016	
**Notes**	
Risk of bias table	
Gøtzsche‐Astrup et al. 2022	
**Notes**	
Risk of bias table	
Han 2014	
**Notes**	
Risk of bias table	
Hasisi et al. 2020	
**Notes**	
Risk of bias table	
Havlickova 2018	
**Notes**	
Risk of bias table	
Horgan 2012	
**Notes**	
Risk of bias table	
Jones 2017	
**Notes**	
Risk of bias table	
Kirisci 2022	
**Notes**	
Risk of bias table	
Klick and Tabarrok 2005	
**Notes**	
Risk of bias table	
Langley et al. 2021	
**Notes**	
Risk of bias table	
Lehrer and Lepage 2020	
**Notes**	
Risk of bias table	
Lindekilde et al. 2021	
**Notes**	
Risk of bias table	
Luke 2014	
**Notes**	
Risk of bias table	
Luke et al. 2013	
**Notes**	
Risk of bias table	
Marion and Cronin 2009	
**Notes**	
Risk of bias table	
Mazerolle et al. 2020	
**Notes**	
Risk of bias table	
Mazerolle et al. 2021	
**Notes**	
Risk of bias table	
Merola and Vovak 2013	
**Notes**	
Risk of bias table	
Okoye 2016	
**Notes**	
Risk of bias table	
Oleszkiewicz 2017	
**Notes**	
Risk of bias table	
Oleszkiewicz et al. 2017	
**Notes**	
Risk of bias table	
Pearce et al. 2019	
**Notes**	
Risk of bias table	
Pearce et al. 2020	
**Notes**	
Risk of bias table	
Perkoski and Chenoweth 2010	
**Notes**	
Risk of bias table	
Pierce 2004	
**Notes**	
Risk of bias table	
Rabbit 2009	
**Notes**	
Risk of bias table	
Randol 2013	
**Notes**	
Risk of bias table	
Regens et al. 2016a	
**Notes**	
Risk of bias table	
Regens et al. 2016b	
**Notes**	
Risk of bias table	
Regens et al. 2017	
**Notes**	
Risk of bias table	
Sanchez‐Cuenca 2009	
**Notes**	
Risk of bias table	
Schaible and Sheffield 2012	
**Notes**	
Risk of bias table	
Scott 2006	
**Notes**	
Risk of bias table	
Scott 2009	
**Notes**	
Risk of bias table	
Shields 2008	
**Notes**	
Risk of bias table	
Skorupski and Uchroński 2018	
**Notes**	
Risk of bias table	
Skurka 2020	
**Notes**	
Risk of bias table	
Sorochinski et al. 2014	
**Notes**	
Risk of bias table	
Stewart 2010	
**Notes**	
Risk of bias table	
Stewart and Oliver 2021	
**Notes**	
Risk of bias table	
Surmon‐Bohr et al. 2020	
**Notes**	
Risk of bias table	
Tankebe 2020	
**Notes**	
Risk of bias table	
Webber et al. 2018	
**Notes**	
Risk of bias table	
White et al. 2014	
**Notes**	
Risk of bias table	
Williams et al. 2016	
**Notes**	
Risk of bias table	
Williams et al. 2019	
**Notes**	
Risk of bias table	
Yang and Jen 2018	
**Notes**	
Risk of bias table	
Footnotes	
Characteristics of excluded studies	
Abrahms & Potter 2015	
**Reason for exclusion**	Ineligible intervention
Adiri 2008	
**Reason for exclusion**	Ineligible intervention
Akartuna & Thornton 2021	
**Reason for exclusion**	Ineligible research design
Al‐Dahash 2017	
**Reason for exclusion**	Ineligible research design
Allen 2012	
**Reason for exclusion**	Ineligible intervention
Alsubaie 2016	
**Reason for exclusion**	Ineligible research design
Altier 2021	
**Reason for exclusion**	Ineligible intervention
Ambrozik 2018	
**Reason for exclusion**	Ineligible intervention
Anderson 2011	
**Reason for exclusion**	Ineligible research design
Aplin & Rogers 2020	
**Reason for exclusion**	Ineligible research design
Argomaniz 2010	
**Reason for exclusion**	Ineligible research design
Asongu & Nwachukwu 2019	
**Reason for exclusion**	Ineligible intervention
Asongu et al. 2019	
**Reason for exclusion**	Ineligible intervention
Ayres 2018	
**Reason for exclusion**	Ineligible intervention
Bailey 2009	
**Reason for exclusion**	Ineligible research design
Bailey & Cree 2011	
**Reason for exclusion**	Ineligible research design
Baker 2016	
**Reason for exclusion**	Ineligible intervention
Balestrini 2021	
**Reason for exclusion**	Ineligible research design
Bali 2009	
**Reason for exclusion**	Ineligible research design
Banks & Tauber 2014	
**Reason for exclusion**	Ineligible research design
Baradaran et al. 2014	
**Reason for exclusion**	Ineligible intervention
Barrett 2009	
**Reason for exclusion**	Ineligible intervention
Barsh 2010	
**Reason for exclusion**	Ineligible research design
Basit 2021	
**Reason for exclusion**	Ineligible intervention
Bastrykin 2021	
**Reason for exclusion**	Ineligible research design
Bazex et al. 2017	
**Reason for exclusion**	Ineligible research design
Beaton & Johnson 2002	
**Reason for exclusion**	
Bennett 2013	
**Reason for exclusion**	Ineligible intervention
Bolhuis & van Wijk 2020	
**Reason for exclusion**	Ineligible intervention
Bonnell et al. 2011	
**Reason for exclusion**	Ineligible research design
Bradley‐Engen et al. 2009	
**Reason for exclusion**	Ineligible research design
Bransford 2012	
**Reason for exclusion**	Ineligible intervention
Brinser & King 2016	
**Reason for exclusion**	Ineligible research design
Brooks 2015	
**Reason for exclusion**	Ineligible intervention
Brown 2013	
**Reason for exclusion**	Ineligible research design
Brown 2014	
**Reason for exclusion**	Ineligible research design
Burruss et al. 2010	
**Reason for exclusion**	Ineligible intervention
Carson 2014	
**Reason for exclusion**	Ineligible research design
Carter et al. 2013	
**Reason for exclusion**	Ineligible research design
Carthy et al. 2020	
**Reason for exclusion**	Ineligible intervention
Celik 2015	
**Reason for exclusion**	Ineligible intervention
Chappell & Gibson 2009	
**Reason for exclusion**	Ineligible intervention
Chenoweth & Dugan 2015	
**Reason for exclusion**	Ineligible intervention
Cherney & Belton 2020	
**Reason for exclusion**	Ineligible research design
Cherney and Belton 2021a	
**Reason for exclusion**	Ineligible research design
Cherney and Belton 2021a	
**Reason for exclusion**	Ineligible research design
Cherney and Murphy 2013	
**Reason for exclusion**	Ineligible research design
Cherney and Murphy 2019	
**Reason for exclusion**	Ineligible research design
Chong and Lopez‐De‐Silanes 2015	
**Reason for exclusion**	Ineligible intervention
Chongruksa et al. 2012	
**Reason for exclusion**	Ineligible intervention
Chou 2015	
**Reason for exclusion**	Ineligible research design
Christensen and Aars 2021	
**Reason for exclusion**	Ineligible research design
Clanton 2013	
**Reason for exclusion**	Ineligible intervention
Clubb et al. 2019	
**Reason for exclusion**	Ineligible intervention
Coffin 2007	
**Reason for exclusion**	Ineligible intervention
Congress 2008	
**Reason for exclusion**	Ineligible research design
Coulthart 2015	
**Reason for exclusion**	Ineligible intervention
Cozine 2010	
**Reason for exclusion**	Ineligible research design
Craun et al. 2019	
**Reason for exclusion**	Ineligible research design
Creel 2015	
**Reason for exclusion**	Ineligible research design
Croissant and Barlow 2007	
**Reason for exclusion**	Ineligible intervention
Csizner 2021	
**Reason for exclusion**	Ineligible research design
Cunningham 2003	
**Reason for exclusion**	Ineligible research design
Cutts 2009	
**Reason for exclusion**	Ineligible research design
Damjanovic et al. 2020	
**Reason for exclusion**	Ineligible intervention
Damphousse and Shields 2007	
**Reason for exclusion**	Ineligible research design
Davies 2018	
**Reason for exclusion**	Ineligible research design
Daxecker 2017	
**Reason for exclusion**	Ineligible intervention
de Oliveira et al. 2018	
**Reason for exclusion**	Ineligible intervention
Decker et al. 2003	
**Reason for exclusion**	Ineligible research design
Degeneffe et al. 2009	
**Reason for exclusion**	Ineligible intervention
Denemark 2012	
**Reason for exclusion**	Ineligible research design
Department of Homeland Security 2015	
**Reason for exclusion**	Ineligible research design
Devine 2007	
**Reason for exclusion**	Ineligible research design
Di Tella and Schargrodsky 2004	
**Reason for exclusion**	Ineligible research design
Dias 2020	
**Reason for exclusion**	Ineligible research design
Dileep and Sekhar 2012	
**Reason for exclusion**	Ineligible intervention
Doan 2012	
**Reason for exclusion**	Ineligible research design
Dombroski 2005	
**Reason for exclusion**	Ineligible intervention
Dombroski and Fischbeck 2006	
**Reason for exclusion**	Ineligible intervention
Donahaue 2011	
**Reason for exclusion**	Ineligible intervention
Donovan and Coupe 2013	
**Reason for exclusion**	Ineligible research design
dos Santos Neto 2014	
**Reason for exclusion**	Ineligible research design
Draca et al. 2011	
**Reason for exclusion**	Ineligible research design
Dragu 2009	
**Reason for exclusion**	Ineligible intervention
Dreher et al. 2010	
**Reason for exclusion**	Ineligible intervention
Drnevich et al. 2009	
**Reason for exclusion**	Ineligible intervention
Dugan 2009	
**Reason for exclusion**	Ineligible research design
Dugan and Chenoweth 2013	
**Reason for exclusion**	Ineligible research design
Dugan and Chenoweth 2018	
**Reason for exclusion**	Ineligible research design
Dugan et al. 2005	
**Reason for exclusion**	Ineligible research design
Duke et al. 2018	
**Reason for exclusion**	Ineligible intervention
Duong 2011	
**Reason for exclusion**	Ineligible intervention
Duvall et al. 2012	
**Reason for exclusion**	Ineligible intervention
Dyrmishi et al. 2021	
**Reason for exclusion**	Ineligible research design
Earle and Soule 2010	
**Reason for exclusion**	Ineligible research design
Ecaterina 2016	
**Reason for exclusion**	Ineligible research design
Eilbert 2005	
**Reason for exclusion**	Ineligible intervention
Ekici 2009	
**Reason for exclusion**	Ineligible intervention
El Amine 2008	
**Reason for exclusion**	Ineligible research design
Ellis 2011	
**Reason for exclusion**	Ineligible intervention
Encina 2020	
**Reason for exclusion**	Ineligible intervention
Enders and Sandler 2011	
**Reason for exclusion**	Ineligible research design
Eser 2008	
**Reason for exclusion**	Ineligible research design
Ezeani 2017	
**Reason for exclusion**	Ineligible intervention
Fair 2012	
**Reason for exclusion**	Ineligible research design
Fairweather 2009	
**Reason for exclusion**	Ineligible research design
Fay and Crutchfield 2019	
**Reason for exclusion**	Ineligible intervention
Feddes and Gallucci 2015	
**Reason for exclusion**	Ineligible research design
Federal Bureau of Investigation 2004	
**Reason for exclusion**	Ineligible research design
Feigenson et al. 2004	
**Reason for exclusion**	Ineligible intervention
Felthous and Saß 2020	
**Reason for exclusion**	Ineligible intervention
Ferzan 2009	
**Reason for exclusion**	Ineligible intervention
Ficara et al. 2021	
**Reason for exclusion**	Ineligible intervention
Florio 2014	
**Reason for exclusion**	Ineligible research design
Fonseca 2010	
**Reason for exclusion**	Ineligible research design
Forbes et al. 2021	
**Reason for exclusion**	Ineligible intervention
Foster 2017	
**Reason for exclusion**	Ineligible intervention
Francis 2011	
**Reason for exclusion**	Ineligible research design
Franco 2009	
**Reason for exclusion**	Ineligible intervention
Franco and Sell 2010	
**Reason for exclusion**	Ineligible intervention
Franco and Sell 2011	
**Reason for exclusion**	Ineligible intervention
Garcia‐Retamero and Dhami 2013	
**Reason for exclusion**	Ineligible research design
Gardner 2002	
**Reason for exclusion**	Ineligible research design
Gardner 2007	
**Reason for exclusion**	Ineligible research design
Gehr 2004	
**Reason for exclusion**	Ineligible research design
George 2018	
**Reason for exclusion**	Ineligible intervention
Gibbs 2012	
**Reason for exclusion**	Ineligible research design
Giblin et al. 2014	
**Reason for exclusion**	Ineligible research design
Gil‐Alana and Barros 2010	
**Reason for exclusion**	Ineligible research design
Gill et al. 2021	
**Reason for exclusion**	Ineligible research design
Giorgio et al. 2018	
**Reason for exclusion**	Ineligible research design
Glomseth and Gottschalk 2009	
**Reason for exclusion**	Ineligible intervention
Gomez 2010	
**Reason for exclusion**	Ineligible intervention
González Agudelo 2011	
**Reason for exclusion**	Ineligible research design
Goodwill et al. 2010	
**Reason for exclusion**	Ineligible research design
Government Accountability Office 2004	
**Reason for exclusion**	Ineligible research design
Gruenewald et al. 2019	
**Reason for exclusion**	Ineligible research design
Guidetti 2006	
**Reason for exclusion**	Ineligible research design
Gyves 2006	
**Reason for exclusion**	Ineligible research design
Gündoğdu 2019	
**Reason for exclusion**	Ineligible intervention
Haglund 2009	
**Reason for exclusion**	Ineligible research design
Haner et al. 2022	
**Reason for exclusion**	Ineligible research design
Harms 2011	
**Reason for exclusion**	Ineligible intervention
Harris‐Hogan et al. 2016	
**Reason for exclusion**	Ineligible research design
Harsch and Maksimov 2019	
**Reason for exclusion**	Ineligible research design
Hasisi and Weisburd 2014	
**Reason for exclusion**	Ineligible research design
Hasisi et al. 2014	
**Reason for exclusion**	Ineligible research design
Heinrich et al. 2013	
**Reason for exclusion**	Ineligible research design
Hirschi and Widmer 2012	
**Reason for exclusion**	Ineligible research design
Hofnung and Margel 2010	
**Reason for exclusion**	Ineligible intervention
Home Office 2010	
**Reason for exclusion**	Ineligible research design
Home Office 2014	
**Reason for exclusion**	Ineligible research design
Home Office 2016	
**Reason for exclusion**	Ineligible intervention
Home Office 2020a	
**Reason for exclusion**	Ineligible research design
Home office 2020b	
**Reason for exclusion**	Ineligible research design
Horne and Bestvater 2016	
**Reason for exclusion**	Ineligible research design
Horowitz 2010	
**Reason for exclusion**	Ineligible intervention
Hsu and McDowall 2020	
**Reason for exclusion**	Ineligible research design
Huq et al. 2011	
**Reason for exclusion**	Ineligible research design
International Association of Chiefs of Police 2005	
**Reason for exclusion**	Ineligible research design
Jackson 2011	
**Reason for exclusion**	Ineligible intervention
Jackson et al. 2021	
**Reason for exclusion**	Ineligible research design
Jansen 2008	
**Reason for exclusion**	Ineligible intervention
Jeffries and Apeh 2020	
**Reason for exclusion**	Ineligible intervention
Jensen et al. 2020	
**Reason for exclusion**	Ineligible intervention
Johnson 2012	
**Reason for exclusion**	Ineligible intervention
Johnson 2016	
**Reason for exclusion**	Ineligible research design
Johnson and Hunter 2017	
**Reason for exclusion**	Ineligible research design
Johnson et al. 2006	
**Reason for exclusion**	Ineligible research design
Jonathan 2010	
**Reason for exclusion**	Ineligible research design
Jones 2010	
**Reason for exclusion**	Ineligible research design
Jones and Brimbal 2017	
**Reason for exclusion**	Ineligible research design
Kaza et al. 2007	
**Reason for exclusion**	Ineligible research design
Kearns 2016	
**Reason for exclusion**	Ineligible research design
Kearns 2020	
**Reason for exclusion**	Ineligible research design
Kennedy 2019	
**Reason for exclusion**	Ineligible research design
Kessing and Andersen 2018	
**Reason for exclusion**	Ineligble research design
Khanna and Zimmermann 2017	
**Reason for exclusion**	Ineligible intervention
Kilburn et al. 2011	
**Reason for exclusion**	Ineligible research design
Kim and de Guzman 2012	
**Reason for exclusion**	Ineligible intervention
King et al. 2018	
**Reason for exclusion**	Ineligible research design
Kingshott 2003	
**Reason for exclusion**	Ineligible intervention
Kirpekar 2007	
**Reason for exclusion**	Ineligible research design
Kollias 2009	
**Reason for exclusion**	Ineligible intervention
Kondrasuk 2004	
**Reason for exclusion**	Ineligible intervention
Konig and Finke 2015	
**Reason for exclusion**	Ineligible intervention
Korte 2005	
**Reason for exclusion**	Ineligible research design
Kristoff 2019	
**Reason for exclusion**	Ineligible intervention
Kılıçlar et al. 2018	
**Reason for exclusion**	Ineligible research design
Lafaye 2021	
**Reason for exclusion**	Ineligible research design
LaFree 2012	
**Reason for exclusion**	Ineligible research design
LaFree et al. 2006	
**Reason for exclusion**	Ineligible research design
LaFree et al. 2020	
**Reason for exclusion**	Ineligible intervention
Lazos 2002	
**Reason for exclusion**	Ineligible intervention
Lee 2010	
**Reason for exclusion**	Ineligible research design
Lee 2014	
**Reason for exclusion**	Ineligible research design
Lesniewicz 2009	
**Reason for exclusion**	Ineligible intervention
Levine et al. 2017	
**Reason for exclusion**	Ineligible research design
Lieberman 2009	
**Reason for exclusion**	Ineligible research design
Lin et al. 2017	
**Reason for exclusion**	Ineligible research design
Lobnikar et al. 2019	
**Reason for exclusion**	Ineligible research design
Luke and Hartwig 2014	
**Reason for exclusion**	Ineligible research design
Lum et al. 2006a	
**Reason for exclusion**	Ineligible intervention
Lum et al. 2006b	
**Reason for exclusion**	Ineligible intervention
Lum et al. 2008	
**Reason for exclusion**	Ineligible intervention
Macpherson 2018	
**Reason for exclusion**	Ineligible research design
Madon et al. 2017	
**Reason for exclusion**	Ineligible research design
Marchment and Gill 2021	
**Reason for exclusion**	Ineligible intervention
Martinez‐Herrera 2002	
**Reason for exclusion**	Ineligible research design
Martonosi 2005	
**Reason for exclusion**	Ineligible intervention
Masleša 2003	
**Reason for exclusion**	Ineligible research design
McCann 2016	
**Reason for exclusion**	Ineligible research design
McCann 2017	
**Reason for exclusion**	Ineligible research design
McClure 2010	
**Reason for exclusion**	Ineligible research design
McGowan 2008	
**Reason for exclusion**	Ineligible research design
McGuirk 2019	
**Reason for exclusion**	Ineligible research design
Mehra 2021	
**Reason for exclusion**	Ineligible research design
Metcalfe and Hodge 2018	
**Reason for exclusion**	Ineligible research design
Meyer 2009	
**Reason for exclusion**	Ineligible research design
Michel and Stys 2014	
**Reason for exclusion**	Ineligible research design
Milla et al. 2020	
**Reason for exclusion**	Ineligible research design
Miller and Hayward 2019	
**Reason for exclusion**	Ineligible intervention
Minhas 2018	
**Reason for exclusion**	Ineligible research design
Moore 2014	
**Reason for exclusion**	Ineligible research design
Moran Blanco 2017	
**Reason for exclusion**	Ineligible research design
Motreff et al. 2020	
**Reason for exclusion**	Ineligible intervention
Motta‐Allen 2008	
**Reason for exclusion**	Ineligible intervention
Muluk et al. 2020	
**Reason for exclusion**	Ineligible research design
Murphy et al. 2019a	
**Reason for exclusion**	Ineligible intervention
Murphy et al. 2019b	
**Reason for exclusion**	Ineligible research design
Murphy et al. 2020	
**Reason for exclusion**	Ineligible research design
Murray et al. 2015	
**Reason for exclusion**	Ineligible research design
Namgung 2013	
**Reason for exclusion**	Ineligible research design
Nash 2017	
**Reason for exclusion**	Ineligible research design
Nesbitt and Hagg 2020	
**Reason for exclusion**	Ineligible research design
Nesbitt et al. 2019	
**Reason for exclusion**	Ineligible intervention
Nicholson and Allely 2021	
**Reason for exclusion**	Ineligible intervention
Norris 2020	
**Reason for exclusion**	Ineligible research design
Norris 2021	
**Reason for exclusion**	Ineligible research design
Norris and Grol‐Prokopczyk 2015	
**Reason for exclusion**	Ineligible research design
Nugent et al. 2005	
**Reason for exclusion**	Ineligible research design
O'Connor 2005	
**Reason for exclusion**	Ineligible intervention
Odabasi 2010	
**Reason for exclusion**	Ineligible research design
Odenwald et al. 2007	
**Reason for exclusion**	Ineligible research design
Omer et al. 2007	
**Reason for exclusion**	Ineligible research design
Onyango 2018	
**Reason for exclusion**	Ineligible research design
Ortiz et al. 2007	
**Reason for exclusion**	Ineligible research design
Ouellet et al. 2017	
**Reason for exclusion**	Ineligible research design
Owonikoko 2020	
**Reason for exclusion**	Ineligible intervention
Ozdogan and Ozdogan 2007	
**Reason for exclusion**	Ineligible research design
Ozgul et al. 2009	
**Reason for exclusion**	Ineligible intervention
Pacheco 2004	
**Reason for exclusion**	Ineligible intervention
Parker and Lindekilde 2020	
**Reason for exclusion**	Ineligible intervention
Parkin et al. 2021	
**Reason for exclusion**	Ineligible research design
Peatfield 2018	
**Reason for exclusion**	Ineligible intervention
Pedersen et al. 2016	
**Reason for exclusion**	Ineligible research design
Pelfrey 2007	
**Reason for exclusion**	Ineligible intervention
Penn et al. 2009	
**Reason for exclusion**	Ineligible research design
Perliger et al. 2009	
**Reason for exclusion**	Ineligible research design
Perry et al. 2017	
**Reason for exclusion**	Ineligible intervention
Phillips 2017	
**Reason for exclusion**	Ineligible research design
Pico‐Malaver et al. 2018	
**Reason for exclusion**	Ineligible research design
Poutvaara and Priks 2006	
**Reason for exclusion**	Ineligible intervention
Poutvaara and Priks 2009	
**Reason for exclusion**	Ineligible research design
Power and Alison 2017	
**Reason for exclusion**	Ineligible research design
Power et al. 2016	
**Reason for exclusion**	Ineligible research design
Price 2017	
**Reason for exclusion**	Ineligible research design
Prosser 2019	
**Reason for exclusion**	Ineligible research design
Pérez‐Armendáriz and Duquette‐Rury 2021	
**Reason for exclusion**	Ineligible intervention
Quick and Choo 2017	
**Reason for exclusion**	Ineligible intervention
Ramic and Dzanic 2017	
**Reason for exclusion**	Ineligible intervention
Randol 2011	
**Reason for exclusion**	Ineligible research design
Reaves and Trotter 2017	
**Reason for exclusion**	Ineligible research design
Regens et al. 2015	
**Reason for exclusion**	Ineligible research design
Rehman 2015	
**Reason for exclusion**	Ineligible intervention
Reiter and Doosje 2021	
**Reason for exclusion**	Ineligible intervention
Restrepo et al. 2006	
**Reason for exclusion**	Ineligible research design
Roberts 2012	
**Reason for exclusion**	Ineligible research design
Roberts et al. 2012	
**Reason for exclusion**	Ineligible research design
Robinson et al. 2019	
**Reason for exclusion**	Ineligible research design
Rosen 2010	
**Reason for exclusion**	Ineligible research design
Safer‐Lichtenstein 2015	
**Reason for exclusion**	Ineligible research design
Saiya and Manchanda 2020	
**Reason for exclusion**	Ineligible intervention
Sandler et al. 2011	
**Reason for exclusion**	Ineligible research design
Sandomir 2009	
**Reason for exclusion**	Ineligible intervention
Savoia et al. 2020	
**Reason for exclusion**	Ineligible intervention
Schafer et al. 2009	
**Reason for exclusion**	Ineligible research design
Schanzer et al. 2016	
**Reason for exclusion**	Ineligible research design
Schmidt and Sikkink 2018	
**Reason for exclusion**	Ineligible intervention
Sehee 2015	
**Reason for exclusion**	Ineligible research design
Sela‐Shayovitz 2015	
**Reason for exclusion**	Ineligible intervention
Shaukenova et al. 2016	
**Reason for exclusion**	Ineligible intervention
Shields et al. 2015	
**Reason for exclusion**	Ineligible research design
Smith 2014	
**Reason for exclusion**	Ineligible intervention
Smith et al. 2002	
**Reason for exclusion**	Ineligible intervention
Smith et al. 2011	
**Reason for exclusion**	Ineligible research design
Spaniel 2019	
**Reason for exclusion**	Ineligible research design
Sproat 2010	
**Reason for exclusion**	Ineligible research design
Sternlieb 2015	
**Reason for exclusion**	Ineligible intervention
Stewart and Morris 2009	
**Reason for exclusion**	Ineligible intervention
Stewart and Mueller 2008	
**Reason for exclusion**	Ineligible research design
Stewart and Mueller 2013a	
**Reason for exclusion**	Ineligible research design
Stewart and Mueller 2013b	
**Reason for exclusion**	Ineligible research design
Stewart and Mueller 2014	
**Reason for exclusion**	Ineligible research design
Stewart and Mueller 2015	
**Reason for exclusion**	Ineligible research design
Sun et al. 2022	
**Reason for exclusion**	Ineligible intervention
Sunstein 2008	
**Reason for exclusion**	Ineligible intervention
Taskarina and Veronika 2021	
**Reason for exclusion**	Ineligible intervention
Thomas 2014	
**Reason for exclusion**	Ineligible research design
Timofeeva and Yavorsky 2016	
**Reason for exclusion**	Ineligible intervention
Trujillo et al. 2009	
**Reason for exclusion**	Ineligible research design
Ugwueze et al. 2022	
**Reason for exclusion**	Ineligible research design
Ullah and Ibrar 2019	
**Reason for exclusion**	Ineligible research design
Urciuoli et al. 2013	
**Reason for exclusion**	Ineligible research design
van Hemert et al 2014	
**Reason for exclusion**	Ineligible research design
Vardalis and Waters 2010	
**Reason for exclusion**	Ineligible research design
Vicino 2006	
**Reason for exclusion**	Ineligible research design
Vogel and Kebbell 2011	
**Reason for exclusion**	Ineligible research design
Waddington 2017	
**Reason for exclusion**	Ineligible research design
Walker 2008	
**Reason for exclusion**	Ineligible research design
Walker 2009b	
**Reason for exclusion**	Ineligible research design
Walker 2009b	
**Reason for exclusion**	Ineligible intervention
Walsh 2011	
**Reason for exclusion**	Ineligible research design
Walsh and Piazza 2010	
**Reason for exclusion**	Ineligible intervention
Weill 2020	
**Reason for exclusion**	Ineligible research design
White et al. 2013	
**Reason for exclusion**	Ineligible research design
Wood 2017	
**Reason for exclusion**	Ineligible research design
Wyckoff 2020	
**Reason for exclusion**	Ineligible research design
Yaoren 2019	
**Reason for exclusion**	Ineligible intervention
Yesberg et al. 2021	
**Reason for exclusion**	Ineligible intervention
Zhirukhina 2018	
**Reason for exclusion**	Ineligible intervention
Černigoj 2020	
**Reason for exclusion**	Ineligible intervention
Footnotes	
Characteristics of studies awaiting classification	
Barak‐Erez 2010	
**Notes**	
Krstic 2019	
**Notes**	
Soto Torres 2010	
**Notes**	
Yoshida 2008	
**Notes**	

## SOURCES OF SUPPORT

**Internal sources**

Griffith Criminology Institute—Strategic Development Grant, Australia

**External sources**

Horizon 2020 (Grant No.: 699824), DHS Science and Technology Directorate, and the Five Research and Development (5RD) Countering Violent Extremism Network, Other

## Supporting information

Supporting information.

Supporting information.
