# Supplementary material for: Criminal justice interventions for preventing radicalisation, violent extremism and terrorism: An evidence and gap map
Source: Campbell Syst Rev. 2023 Nov 14;19(4):e1366. doi: 10.1002/cl2.1366 (PMC10644945; doi:10.1002/cl2.1366)
Supplement: Supplementary file 1 — Supporting information. [file CL2-19-e1366-s001.docx]

# Appendices

## 1 GPD Systematic search strategy

### Search Terms

To ensure optimum sensitivity and specificity, the GPD search strategy utilises a combination of free-text and controlled vocabulary search terms. Because controlled vocabularies and search capabilities vary across databases, the exact combination of search terms and field codes are adapted to each database. Final search syntax for each location will be reported in the final review.

The free-text search terms for the GPD are provided in Table 1 and are grouped by substantive (i.e., some form of policing) and evaluation terminology. Although the search strategy may vary slightly across search locations, it follows a number of general rules:

- Search terms are combined into search strings using Boolean operators “AND” and “OR”. Specifically, terms within each category are combined with “OR”, and categories will be combined with “AND”. For example: (police OR policing OR “law#enforcement”) AND (analy* OR ANCOVA OR ANOVA OR …).
- Compound terms (e.g., law enforcement) are considered single terms in search strings by using quotation marks (i.e., “law*enforcement”) to ensure that the database searches for the entire term rather than separate words.
- Wild cards and truncation codes are used for search terms with multiple iterations from a stem word (e.g., evaluation, evaluate) or spelling variations (e.g., evaluat* or randomi#e).
- If a database has a controlled vocabulary term that is equivalent to “POLICE”, the term is combined in a search string that includes both the policing and evaluation free-text search terms. This approach ensures that the search retrieves documents that do not use policing terms in the title/abstract but have been indexed as being related to policing in the database. An example of this approach is the following search string: (((SU: “POLICE”) OR (TI,AB,KW: police OR policing OR “law*enforcement”)) AND (TI,AB,KW: intervention* OR evaluat* OR compar* OR …)).
- For search locations with limited search functionality, a broad search that uses only the policing free-text terms is implemented.
- Multidisciplinary database searches are limited to relevant disciplines (e.g., include social sciences but exclude physical sciences).
- Search results are refined to exclude specific types of documents that are not suitable for systematic reviews (e.g., newspapers, front/back matter, book reviews).

#### Table 1. Free-text search terms for the GPD systematic search

| **Policing Search Terms** | **Evaluation Search Terms** | | | |
| --- | --- | --- | --- | --- |
| police  policing  “law*enforcement”  constab*  detective*  sheriff* | analy*  ANCOVA  ANOVA  “ABAB design”  “AB design”  baseline  causa*  “chi#square”  coefficient*  “comparison condition*”  “comparison group*”  “control condition*”  “control group*”  correlat*  covariat*  “cross#section*” | data  effect*  efficacy  eval*  experiment*  hypothes*  impact*  intervent*  interview*  longitudinal  MANCOVA  MANOVA  “matched group”  measure*  “meta-analy*”  “odds#ratio* | outcome*  paramet*  “post-test”  posttest  “post test”  predict*  “pre-test”  pretest  program*  “propensity score*”  quantitative  “quasi#experiment*”  questionnaire*  random*  RCT  regress* | result*  “risk#ratio*”  sampl*  “standard deviation*”  statistic*  studies  study  survey*  “systematic review*”  “t#test*”  “time#series”  treatment*  variable*  variance |

### Search Locations

To reduce publication and discipline bias, the GPD search strategy adopts an international scope and involves searching for literature across a number of disciplines (e.g., criminology, law, political science, public health, sociology, social science and social work). The search captures a comprehensive range of published (i.e., journal articles, book chapters, books) and unpublished literature (e.g., working papers, governmental reports, technical reports, conference proceedings, dissertations) by implementing a search strategy across bibliographic/academic, grey literature, and dissertation databases or repositories.

It is noted that there is substantial overlap of the content coverage between many of the databases. Therefore, the *Optimal Searching of Indexing Databases* (OSID) computer program (Neville & Higginson, 2014) has been used to analyse the content crossover for all databases that have accessible content coverage lists. OSID analyses the content coverage and creates a search location solution that provides the most comprehensive coverage via the least number of databases. Another advantage of using OSID when designing a search strategy is the reduction in the number of duplicates that would need to be removed prior to the screening phase. Databases with >10 unique titles are searched in full, whereas databases with ≤10 unique titles were searched only the unique titles and any non-serial content (e.g., reports, conference proceedings). Where a modified search of a database would be more labour-intensive than a full search and export results, a full search of the database is conducted. The final search locations and solutions are reported in Table 2.

#### Table 2. GPD search locations and protocol (January 1^st^ 1950 – December 2019)

| **INDEXED & ACADEMIC DATABASES** |  | **CONTENT COVERAGE FED INTO OSID?** | **FULL OR MODIFIED SEARCH?** | **SEARCH MODIFICATIONS** |
| --- | --- | --- | --- | --- |
| **ProQuest** | Criminal Justice | Yes | Full | None. |
|  | Dissertation and Theses Database Global | Not Available | Modified | Social Sciences subset. |
|  | Political Science | Yes | Full | None. |
|  | Periodical Archive Online | Yes | Full | None. |
|  | Research Library | Yes | Modified | Social Sciences subset. |
|  | Social Science Journals | Yes | Full | None. |
|  | Sociology | Yes | Modified | Search 2 unique journal titles and non-serial content only. |
|  | Applied Social Sciences Index and Abstracts | Yes | Full | None. |
|  | International Bibliography of the Social Sciences | Yes | Full | None. |
|  | Public Affairs Information Service | Yes | Full | None. |
|  | Social Services Abstracts | Yes | Modified | Search 5 unique journal titles and non-serial content only. |
|  | Sociological Abstracts | Yes | Full | None. |
|  | Worldwide Political Sciences Abstracts | Yes | Modified | Search 9 unique journal titles and non-serial content only. |
| **EBSCO** | Academic Search Premier | Yes | Full | None. |
|  | Criminal Justice Abstracts | Yes | Full | None. |
|  | EconLit | Yes | Full | None. |
|  | MEDLINE with Full-Text (for initial search 1950-2014) | Yes | Full | None. |
|  | Social Sciences Full-Text | Yes | Full | None. |
| **OVID** | International Political Science Abstracts | Not Available | Full | None. |
|  | PsycARTICLES | Yes | Modified | Search 4 unique journal titles only. |
|  | PsycEXTRA | Not Available | Full | None. |
|  | PsycINFO | Yes | Full | None. |
|  | Social Work Abstracts | Not Available | Full | None. |
| **Web of Science** | Current Contents Connect – Social and Behavioural Sciences Edition | Yes | Modified | Search 1 unique journal title and non-serial content only. |
|  | Book Citation Index (Social Sciences and Humanities) | Not Available | Full | None. |
|  | Conference Proceedings Citation Index (Social Sciences and Humanities) | Not Available | Full | None. |
|  | MEDLINE with Full-Text (for searches 2015 onwards) | Yes | Full | None. |
|  | Social Science Citation Index | Yes | Full | None. |
| **Informit** | Australian Attorney General Information Service | Yes | Full | None. |
|  | Australian Criminology Database (CINCH) | Yes | Full | None. |
|  | Australian Federal Police Database | Yes | Full | None. |
|  | Australian Public Affairs Full-Text | Yes | Full | None. |
|  | DRUG | Yes | Full | None. |
|  | Health & Society Database | Yes | Modified | Search unique journal titles and non-serial content only. |
|  | Humanities and Social Sciences Collection | Yes | Full | None. |
| **Gale-Cengage** | Expanded Academic ASAP | Yes | Full | None. |
| **STANDALONE & OPEN ACCESS DATABASES** | Cambridge Journals Online | Yes | Modified | Search 4 unique journal titles in Law and Political Science collections and full search of Social Studies collection. |
|  | Directory of Open Access Journals | Yes | Full | None. |
|  | HeinOnline | Yes | Modified | Law Journals Online collection only. |
|  | JSTOR | Yes | Modified | Search unique titles across the Law, Political Science, Public Health, Public Policy, Social Work and Sociology collections only. The Criminal Justice collection had no unique content and so will be excluded from the search. Only 10% of content in this database have abstracts and a full-text search returns >250,000 results because of inability to construct complex search strings. Therefore, a modified search of the unique titles across these collections will be more pragmatic than a full search of the database. |
|  | Oxford Scholarship Online | Yes | Full | None. |
|  | Sage Journals Online and Archive (Sage Premier) | Yes | Modified | Search 5 unique journal titles and non-serial content only. |
|  | ScienceDirect | Yes | Full | None. |
|  | SCOPUS | Yes | Full | None. |
|  | SpringerLink | Yes | Full | Although this database has low uniqueness when combined with the full set of databases, a full search using only the policing search terms will be more pragmatic than a modified search on unique titles because of the restricted search functionality of this database. |
|  | Taylor & Francis Online | Yes | Modified | Although this database has low uniqueness when combined with the full set of databases, a full search using only the policing search terms will be more pragmatic than a modified search on unique titles because of the restricted search functionality of this database. |
|  | Wiley Online Library | Yes | Full | None. |
|  | California Commission on Peace Officer Standards & Training Library | No | Full | None. |
|  | Cochrane Library | No | Full | None. |
|  | CrimeSolutions.gov | No | Full | None. |
|  | Database of Abstracts of Reviews of Effectiveness (DARE) | No | Full | None. |
|  | FBI – The Fault (Reports and Publications) | No | Full | None. |
|  | Evidence-Based Policing Matrix | No | Full | None. |
|  | International Initiative for Impact Evaluation Database (3ie) | No | Full | None. |
|  | National Criminal Justice Reference Service | No | Full | None. |
|  | Safety Lit Database | No | Full | None. |
|  | Australian Institute of Criminology | No | Full | None. |
|  | Bureau of Police Research and Development (India) | No | Full | None. |
|  | Canadian Police Research Catalogue | No | Full | None. |
|  | Centre for Problem-Oriented Policing | No | Full | None. |
|  | College of Policing (including POLKA and Crime Reduction Toolkit) | No | Full | None. |
|  | European Police College (CEPOL) | No | Full | None. |
|  | Evidence for Policy and Practice Information and Coordinating Centre | No | Full | None. |
|  | National Research Institute of Police Science (Japanese) | No | Full | None. |
|  | Office of Community Oriented Policing Services | No | Full | None. |
|  | Police Executive Research Forum (US) | No | Full | None. |
|  | Police Foundation (US) | No | Full | None. |
|  | Tasmania Institute of Law Enforcement Studies (Australia) | No | Full | None. |
|  | Policing Online Information System (POLIS, Europe) | No | Full | None. |
|  | Scottish Institute for Policing Research | No | Full | None. |
|  | Centre of Excellence in Policing and Security (Australian, now archived) | No | Full | None. |
|  | Alcohol and Alcohol Problems Science Database (ETOH, now archived) | No | Full | None. |
|  | African Journals Online | No | Full | None. |
|  | Campbell Collaboration Library of Systematic Reviews | No | Full | None. |
|  | Criminal Justice Press (Crime Prevention Studies, volumes 1-27) | No | Full | None. |
|  | Danish National Police (Politi) | No | Full | None. |
|  | Drug Policy Alliance – Lindesmith Library (Online Resource Library) | No | Full | None. |
|  | DrugScope | No | Full | None. |
|  | Finnish Police (Poliisi) | No | Full | None. |
|  | GeoRef | No | Full | None. |
|  | German Federal Criminal Police Office (Bundeskriminalamt) | No | Full | None. |
|  | Home Office (United Kingdom) | No | Full | None. |
|  | Indian Citation Index (Social Science and Humanities Subset) | No | Full | None. |
|  | Institute for Law and Justice | No | Full | None. |
|  | Jill Dando Institute of Crime Science (JDI) | No | Full | None. |
|  | Justice Research and Statistics Association- State Statistical Analysis Centers | No | Full | None. |
|  | Ministry of Justice (United Kingdom) | No | Full | None. |
|  | Netherlands Institute for the Study of Crime and Law Enforcement (NSCR) | No | Full | None. |
|  | Netherlands Police (Politie) | No | Full | None. |
|  | New Zealand Ministry of Justice | No | Full | None. |
|  | New Zealand Police | No | Full | None. |
|  | Norwegian Ministry of Justice and the Police | No | Full | None. |
|  | Royal Canadian Mounted Police | No | Full | None. |
|  | SAGE Knowledge | No | Full | None. |
|  | Swedish National Council on Crime Prevention (Brå) | No | Full | None. |
|  | Swedish Police Service | No | Full | None. |
|  | Urban Institute | No | Full | None. |
|  | YU-DSpace Repository | No | Full | None. |

## 2 GPD Systematic Compilation Strategy

### Inclusion Criteria

Each record captured by the GPD systematic search must satisfy all inclusion criteria to be included in the GPD: timeframe, intervention and research design. There are no restrictions applied to the types of outcomes, participants, settings or languages considered eligible for inclusion in the GPD.

#### Types of interventions

Each document must contain an impact evaluation of a policing intervention. Policing interventions are defined as some kind of a strategy, program, technique, approach, activity, campaign, training, directive, or funding/organisational change that involves police in some way (other agencies or organisations can be involved). Police involvement is broadly defined as:

- Police initiation, development or leadership
- Police are recipients of the intervention or the intervention is related, focused or targeted to police practices
- Delivery or implementation of the intervention by police

#### Types of study designs

The GPD includes quantitative impact evaluations of policing interventions that utilise randomised experimental (e.g., RCTs) or quasi-experimental evaluation designs with a valid comparison group that does not receive the intervention. The GPD includes designs where the comparison group receives ‘business-as-usual’ policing, no intervention or an alternative intervention (treatment-treatment designs).

The specific list of research designs included in the GPD are as follows:

- Systematic reviews with or without meta-analyses
- Cross-over designs
- Cost-benefit analyses
- Regression discontinuity designs
- Designs using multivariate controls (e.g., multiple regression)
- Matched control group designs with or without pre-intervention baseline measures (propensity or statistically matched)
- Unmatched control group designs with pre-post intervention measures which allow for difference-in-difference analysis
- Unmatched control group designs without pre-intervention measures where the control group has face validity
- Short interrupted time-series designs with control group (less than 25 pre- and 25 post-intervention observations)
- Long interrupted time-series designs with or without a control group (≥25 pre- and post-intervention observations)
- Raw unadjusted correlational designs where the variation in the level of the intervention is compared to the variation in the level of the outcome

The GPD excludes single group designs with pre- and post-intervention measures as these designs are highly subject to bias and threats to internal validity.

### Systematic Screening

To establish eligibility, records captured by the GPD search are progress through a series of systematic stages which are summarised in Table 1, with additional detail provided in the following subsections.

All research staff working on the GPD undergo standardised training before beginning work within any of the stages detailed below. Staff then complete short training simulations to enable an assessment of their understanding of the GPD protocols and highlight any areas for additional training. In addition, random samples of each staff’s work are regularly cross-checked to ensure adherence to protocols. Disagreements about screening decisions between staff are mediated by either the project manager or GPD chief investigators.

#### Title and abstract screening

After removing duplicates, the title and abstract of records captured by the GPD systematic search is screened by trained research staff to identify potentially eligible research that satisfies the following criteria:

- Document is dated between 1950 – present
- Document is unique (i.e., not a duplicate)
- Document is about police or policing
- Document is an eligible document type (e.g., not a book review)

Records are excluded if the answer to any one of the criteria is unambiguously ‘No’, and will be classified as potentially eligible otherwise. Records classified as eligible at the title and abstract screening stage progress to full-text document retrieval and screening stages.

#### Full-text eligibility screening

Wherever possible, a full-text electronic version of an eligible record is imported into *SysReview* (review management software; Higginson & Neville, 2015). For records without an electronic version, a hardcopy of the record is located to enable full-text eligibility screening. The full-text of each document is screened to identify studies that satisfy the following criteria:

- Document is dated between 1950 – present
- Document is unique
- Document reports a quantitative statistical comparison
- Document reports on policing evaluation
- Document reports in a quantitative impact evaluation of a policing intervention
- Evaluation uses an eligible research design

| **SYSTEMATIC SEARCH OF PUBLISHED & UNPUBLISHED LITERATURE** |
| --- |
| ⇓ |
| **EXPORT SEARCH RESULTS**   - Bibliographic data and abstracts exported into EndNote - Data cleaned and duplicate records removed |
| ⇓ |
| **IMPORT SEARCH RESULTS INTO *SYSREVIEW*** |
| ⇓ |
| **SCREEN TITLES AND ABSTRACTS FOR ELIGIBILITY**   1. Not a duplicate document? 2. Between 1950 – present? 3. About police or policing? 4. Eligible document type?   ***If not clearly excluded on any criteria…*** |
| ⇓ |
| **DOCUMENT RETRIEVAL**   - Retrieve electronic and hard copies of all eligible documents - Attach electronic versions to records in *SysReview* |
| ⇓ |
| **SCREEN FULL-TEXT OF DOCUMENTS**  **FOR FINAL ELIGIBILITY**   1. Not a duplicate document? 2. Between 1950 – present? 3. Quantitative statistical comparison? 4. Policing intervention? 5. Quantitative impact evaluation? 6. Eligible research design?   ***If ‘Yes’ to all…*** |
| ⇓ |
| **CATEGORISE ELIGIBLE DOCUMENTS**   1. Research design 2. Intervention location 3. Publication date 4. Problem targeted 5. Evaluation outcome measure(s) 6. Type of policing intervention |
| ⇓ |
| **GLOBAL POLICING DATABASE (GPD)**  Web-based  Searchable  Updated biennially |

*Table 1.* GPD systematic compilation process

## 3 Search syntax for other database searches

| **PsycINFO (OVID)** | |
| --- | --- |
| **Search date** | 08/06/2022 |
| **Search notes** | APA PsycBooks <1806 to May 2022>  APA PsycInfo <1806 to May Week 5 2022> |
| 1 | (extremis* or "far left*" or "far-left*" or "far right*" or "far-right*" or "foreign fight*" or "foreign-fight*" or "freedom fight*" or "freedom-fight*" or guerrilla or "homeland security" or "ideological violence*" or "ideologically motivat*" or "ideologically-motivat*" or indoctrinat* or "left wing*" or "left-wing*" or "lone wol*" or "lone-wol*" or militant* or "national security" or "political violence*" or "politically motivat*" or "politically-motivat*" or radicali* or rebel* or "religious violence*" or "religiously motivat*" or "religiously-motivat*" or "right wing*" or "right-wing*" or "single issue*" or "single-issue*" or supremacis* or terror* or vigilante* or vigilantism or deradicali* or "de-radicali*" or "counter-terror*" or counterterror* or "counter-extremis*" or counterextremis* or separatis* or militia* or jihad*).ab,hw,id,mh,ot,ti. |
| 2 | (accused or acquit* or adjourn* or adjudicat* or admiss* or affida* or appeal* or appellate or apprehend* or arbitrat* or arraign* or arrest* or attorney* or authorit* or bail* or barrister* or breach* or "case manage*" or "case-manage*" or caution* or charge* or clerk* or confinement* or convict* or coroner* or correction* or court* or crime* or criminal* or "cross examin*" or "cross-examin*" or custod* or defendant* or defense or defence or detain* or detention* or deter* or divert* or diversion* or enforc* or execut* or felon* or forensic* or gaol* or guilt* or "high security" or "high-security" or "halfway house" or "halfway-house" or imprison* or incarcerat* or indict* or infract* or infring* or injunct* or inquest* or innocen* or inmate* or juris* or jail* or judge* or judic* or juror* or juries or jury or justice or law* or legal* or legislat* or litigat* or "low security" or "low-security" or magistrate* or mandat* or mitigat* or marshal* or misdem* or "medium security*" or "medium-security*" or offend* or offence* or officer* or official* or ordinance or parole* or pardon* or penal* or plea* or pre-admiss* or pre-arrest* or pre-imprison* or pre-trial* or precedent* or prevent* or prison* or probat* or prohibit* or prosecut* or punish* or recividis* or rehab* or reintegrat* or remand* or reoffend* or "re-offend*" or ruling* or sanction* or sentenc* or solicitor* or statut* or subpoena* or supervis* or surveil* or suspect* or testif* or testimon* or trial* or tribunal* or verdict* or victim* or witness*).ab,hw,id,mh,ot,ti. |
| 3 | ("comparison condition*" or "comparison-condition*" or "comparison group*" or "comparison-group*" or "control condition*" or "control-condition*" or "control group*" or "control-group*" or effective or efficac* or evaluat* or experiment* or intervent* or "matched group*" or "matched-group*" or program* or "quasi-experiment*" or "quasiexperiment*" or "quasi experiment*" or random* or RCT or treatment* or trial*).ab,hw,id,mh,ot,ti. |
| 4 | 1 and 2 and 3 |
| 5 | limit 4 to yr="2002 - 2021" |
| 6 | remove duplicates from 5 |
| **Social Science Citation Index, Conference Proceedings Index and Emerging Sources Index (Web of Science)** | |
| **Search date** | 30/06/2022 |
| **Search notes** | Databases were searched concurrently. TS = searching on title, abstract, author keywords, and Keywords Plus |
| 1 | TS=(extremis* or "far left*" or "far-left*" or "far right*" or "far-right*" or "foreign fight*" or "foreign-fight*" or "freedom fight*" or "freedom-fight*" or guerrilla or "homeland security" or "ideological violence*" or "ideologically motivat*" or "ideologically-motivat*" or indoctrinat* or "left wing*" or "left-wing*" or "lone wol*" or "lone-wol*" or militant* or "national security" or "political violence*" or "politically motivat*" or "politically-motivat*" or radicali* or rebel* or "religious violence*" or "religiously motivat*" or "religiously-motivat*" or "right wing*" or "right-wing*" or "single issue*" or "single-issue*" or supremacis* or terror* or vigilante* or vigilantism or deradicali* or "de-radicali*" or "counter-terror*" or counterterror* or "counter-extremis*" or counterextremis* or separatis* or militia* or jihad*) |
| 2 | TS=(accused or acquit* or adjourn* or adjudicat* or admiss* or affida* or appeal* or appellate or apprehend* or arbitrat* or arraign* or arrest* or attorney* or authorit* or bail* or barrister* or breach* or "case manage*" or "case-manage*" or caution* or charge* or clerk* or confinement* or convict* or coroner* or correction* or court* or crime* or criminal* or "cross examin*" or "cross-examin*" or custod* or defendant* or defense or defence or detain* or detention* or deter* or divert* or diversion* or enforc* or execut* or felon* or forensic* or gaol* or guilt* or "high security" or "high-security" or "halfway house" or "halfway-house" or imprison* or incarcerat* or indict* or infract* or infring* or injunct* or inquest* or innocen* or inmate* or juris* or jail* or judge* or judic* or juror* or juries or jury or justice or law* or legal* or legislat* or litigat* or "low security" or "low-security" or magistrate* or mandat* or mitigat* or marshal* or misdem* or "medium security*" or "medium-security*" or offend* or offence* or officer* or official* or ordinance or parole* or pardon* or penal* or plea* or pre-admiss* or pre-arrest* or pre-imprison* or pre-trial* or precedent* or prevent* or prison* or probat* or prohibit* or prosecut* or punish* or recividis* or rehab* or reintegrat* or remand* or reoffend* or "re-offend*" or ruling* or sanction* or sentenc* or solicitor* or statut* or subpoena* or supervis* or surveil* or suspect* or testif* or testimon* or trial* or tribunal* or verdict* or victim* or witness*) |
| 3 | TS=("comparison condition*" or "comparison-condition*" or "comparison group*" or "comparison-group*" or "control condition*" or "control-condition*" or "control group*" or "control-group*" or effective or efficac* or evaluat* or experiment* or intervent* or "matched group*" or "matched-group*" or program* or "quasi-experiment*" or "quasiexperiment*" or "quasi experiment*" or random* or RCT or treatment* or trial*) |
| 4 | 1 and 2 and 3 |
| 5 | #4 AND Timespan: 2002-01-01 to 2021-12-31 (Publication Date) |
| 6 | #5 AND Exclude document types: Editorial Materials, Book Reviews, Corrections, Biographic-Items, Chronologies, News Items |
| **Medline (Web of Science)** | |
| **Search date** | 12/07/2022 |
| **Search notes** | TS = searching on title, abstract, author keywords, and Keywords Plus |
| 1 | TS=(extremis* or "far left*" or "far-left*" or "far right*" or "far-right*" or "foreign fight*" or "foreign-fight*" or "freedom fight*" or "freedom-fight*" or guerrilla or "homeland security" or "ideological violence*" or "ideologically motivat*" or "ideologically-motivat*" or indoctrinat* or "left wing*" or "left-wing*" or "lone wol*" or "lone-wol*" or militant* or "national security" or "political violence*" or "politically motivat*" or "politically-motivat*" or radicali* or rebel* or "religious violence*" or "religiously motivat*" or "religiously-motivat*" or "right wing*" or "right-wing*" or "single issue*" or "single-issue*" or supremacis* or terror* or vigilante* or vigilantism or deradicali* or "de-radicali*" or "counter-terror*" or counterterror* or "counter-extremis*" or counterextremis* or separatis* or militia* or jihad*) |
| 2 | TS=(accused or acquit* or adjourn* or adjudicat* or admiss* or affida* or appeal* or appellate or apprehend* or arbitrat* or arraign* or arrest* or attorney* or authorit* or bail* or barrister* or breach* or "case manage*" or "case-manage*" or caution* or charge* or clerk* or confinement* or convict* or coroner* or correction* or court* or crime* or criminal* or "cross examin*" or "cross-examin*" or custod* or defendant* or defense or defence or detain* or detention* or deter* or divert* or diversion* or enforc* or execut* or felon* or forensic* or gaol* or guilt* or "high security" or "high-security" or "halfway house" or "halfway-house" or imprison* or incarcerat* or indict* or infract* or infring* or injunct* or inquest* or innocen* or inmate* or juris* or jail* or judge* or judic* or juror* or juries or jury or justice or law* or legal* or legislat* or litigat* or "low security" or "low-security" or magistrate* or mandat* or mitigat* or marshal* or misdem* or "medium security*" or "medium-security*" or offend* or offence* or officer* or official* or ordinance or parole* or pardon* or penal* or plea* or pre-admiss* or pre-arrest* or pre-imprison* or pre-trial* or precedent* or prevent* or prison* or probat* or prohibit* or prosecut* or punish* or recividis* or rehab* or reintegrat* or remand* or reoffend* or "re-offend*" or ruling* or sanction* or sentenc* or solicitor* or statut* or subpoena* or supervis* or surveil* or suspect* or testif* or testimon* or trial* or tribunal* or verdict* or victim* or witness*) |
| 3 | TS=("comparison condition*" or "comparison-condition*" or "comparison group*" or "comparison-group*" or "control condition*" or "control-condition*" or "control group*" or "control-group*" or effective or efficac* or evaluat* or experiment* or intervent* or "matched group*" or "matched-group*" or program* or "quasi-experiment*" or "quasiexperiment*" or "quasi experiment*" or random* or RCT or treatment* or trial*) |
| 4 | 1 and 2 and 3 |
| 5 | #4 AND Timespan: 2002-01-01 to 2021-12-31 (Publication Date) |
| 6 | (#5) NOT DT=(Address OR Addresses OR Autobiography OR Bibliography OR Biography OR Corrected And Republished Article OR Dictionary OR Directory OR Duplicate Publication OR Editorial OR Guideline OR Interactive Tutorial OR Interview OR Introductory Journal Article OR Lecture OR Lectures OR Legal Case OR Legal Cases OR Legislation OR Letter OR News OR Newspaper Article OR Patient Education Handout OR Periodical Index OR Personal Narrative OR Personal Narratives OR Portrait OR Portraits OR Practice Guideline OR Published Erratum OR Retracted Publication OR Retraction Of Publication OR Video Audio Media OR Webcast OR Webcasts) |
| **SciELO (Web of Science)** | |
| **Search date** | 12/07/2022 |
| **Search notes** | TS = searching on title, abstract, author keywords, and Keywords Plus |
| 1 | TS=(extremis* or "far left*" or "far-left*" or "far right*" or "far-right*" or "foreign fight*" or "foreign-fight*" or "freedom fight*" or "freedom-fight*" or guerrilla or "homeland security" or "ideological violence*" or "ideologically motivat*" or "ideologically-motivat*" or indoctrinat* or "left wing*" or "left-wing*" or "lone wol*" or "lone-wol*" or militant* or "national security" or "political violence*" or "politically motivat*" or "politically-motivat*" or radicali* or rebel* or "religious violence*" or "religiously motivat*" or "religiously-motivat*" or "right wing*" or "right-wing*" or "single issue*" or "single-issue*" or supremacis* or terror* or vigilante* or vigilantism or deradicali* or "de-radicali*" or "counter-terror*" or counterterror* or "counter-extremis*" or counterextremis* or separatis* or militia* or jihad*) |
| 2 | TS=(accused or acquit* or adjourn* or adjudicat* or admiss* or affida* or appeal* or appellate or apprehend* or arbitrat* or arraign* or arrest* or attorney* or authorit* or bail* or barrister* or breach* or "case manage*" or "case-manage*" or caution* or charge* or clerk* or confinement* or convict* or coroner* or correction* or court* or crime* or criminal* or "cross examin*" or "cross-examin*" or custod* or defendant* or defense or defence or detain* or detention* or deter* or divert* or diversion* or enforc* or execut* or felon* or forensic* or gaol* or guilt* or "high security" or "high-security" or "halfway house" or "halfway-house" or imprison* or incarcerat* or indict* or infract* or infring* or injunct* or inquest* or innocen* or inmate* or juris* or jail* or judge* or judic* or juror* or juries or jury or justice or law* or legal* or legislat* or litigat* or "low security" or "low-security" or magistrate* or mandat* or mitigat* or marshal* or misdem* or "medium security*" or "medium-security*" or offend* or offence* or officer* or official* or ordinance or parole* or pardon* or penal* or plea* or pre-admiss* or pre-arrest* or pre-imprison* or pre-trial* or precedent* or prevent* or prison* or probat* or prohibit* or prosecut* or punish* or recividis* or rehab* or reintegrat* or remand* or reoffend* or "re-offend*" or ruling* or sanction* or sentenc* or solicitor* or statut* or subpoena* or supervis* or surveil* or suspect* or testif* or testimon* or trial* or tribunal* or verdict* or victim* or witness*) |
| 3 | TS=("comparison condition*" or "comparison-condition*" or "comparison group*" or "comparison-group*" or "control condition*" or "control-condition*" or "control group*" or "control-group*" or effective or efficac* or evaluat* or experiment* or intervent* or "matched group*" or "matched-group*" or program* or "quasi-experiment*" or "quasiexperiment*" or "quasi experiment*" or random* or RCT or treatment* or trial*) |
| 4 | 1 and 2 and 3 |
| 5 | #4 AND Timespan: 2002-01-01 to 2021-12-31 (Publication Date) |
| 6 | (#5) NOT DT=(Addendum OR Announcement OR Editorial OR Letter OR News OR Oration OR Press Release OR Rapid-Communication) |
| **Criminal Justice Abstracts (EBSCO)** | |
| **Search date** | 12/07/2022 |
| **Search notes** | Searched on TI (title), AB (abstract or author-supplied abstract) and KW (author-supplied keywords) |
| 1 | TI ( (extremis* OR "far left*" OR "far-left*" OR "far right*" OR "far-right*" OR "foreign fight*" OR "foreign-fight*" OR "freedom fight*" OR "freedom-fight*" OR guerrilla OR "homeland security" OR "ideological violence*" OR "ideologically motivat*" OR "ideologically-motivat*" OR indoctrinat* OR "left wing*" OR "left-wing*" OR "lone wol*" OR "lone-wol*" OR militant* OR "national security" OR "political violence*" OR "politically motivat*" OR "politically-motivat*" OR radicali* OR rebel* OR "religious violence*" OR "religiously motivat*" OR "religiously-motivat*" OR "right wing*" OR "right-wing*" OR "single issue*" OR "single-issue*" OR supremacis* OR terror* OR vigilante* OR vigilantism OR deradicali* OR "de-radicali*" OR "counter-terror*" OR counterterror* OR "counter-extremis*" OR counterextremis* OR separatis* OR militia* OR jihad*) ) OR AB ( (extremis* OR "far left*" OR "far-left*" OR "far right*" OR "far-right*" OR "foreign fight*" OR "foreign-fight*" OR "freedom fight*" OR "freedom-fight*" OR guerrilla OR "homeland security" OR "ideological violence*" OR "ideologically motivat*" OR "ideologically-motivat*" OR indoctrinat* OR "left wing*" OR "left-wing*" OR "lone wol*" OR "lone-wol*" OR militant* OR "national security" OR "political violence*" OR "politically motivat*" OR "politically-motivat*" OR radicali* OR rebel* OR "religious violence*" OR "religiously motivat*" OR "religiously-motivat*" OR "right wing*" OR "right-wing*" OR "single issue*" OR "single-issue*" OR supremacis* OR terror* OR vigilante* OR vigilantism OR deradicali* OR "de-radicali*" OR "counter-terror*" OR counterterror* OR "counter-extremis*" OR counterextremis* OR separatis* OR militia* OR jihad*) ) OR KW ( (extremis* OR "far left*" OR "far-left*" OR "far right*" OR "far-right*" OR "foreign fight*" OR "foreign-fight*" OR "freedom fight*" OR "freedom-fight*" OR guerrilla OR "homeland security" OR "ideological violence*" OR "ideologically motivat*" OR "ideologically-motivat*" OR indoctrinat* OR "left wing*" OR "left-wing*" OR "lone wol*" OR "lone-wol*" OR militant* OR "national security" OR "political violence*" OR "politically motivat*" OR "politically-motivat*" OR radicali* OR rebel* OR "religious violence*" OR "religiously motivat*" OR "religiously-motivat*" OR "right wing*" OR "right-wing*" OR "single issue*" OR "single-issue*" OR supremacis* OR terror* OR vigilante* OR vigilantism OR deradicali* OR "de-radicali*" OR "counter-terror*" OR counterterror* OR "counter-extremis*" OR counterextremis* OR separatis* OR militia* OR jihad*) ) |
| 2 | TI ( (accused OR acquit* OR adjourn* OR adjudicat* OR admiss* OR affida* OR appeal* OR appellate OR apprehend* OR arbitrat* OR arraign* OR arrest* OR attorney* OR authorit* OR bail* OR barrister* OR breach* OR "case manage*" OR "case-manage*" OR caution* OR charge* OR clerk* OR confinement* OR convict* OR coroner* OR correction* OR court* OR crime* OR criminal* OR "cross examin*" OR "cross-examin*" OR custod* OR defendant* OR defense OR defence OR detain* OR detention* OR deter* OR divert* OR diversion* OR enforc* OR execut* OR felon* OR forensic* OR gaol* OR guilt* OR "high security" OR "high-security" OR "halfway house" OR "halfway-house" OR imprison* OR incarcerat* OR indict* OR infract* OR infring* OR injunct* OR inquest* OR innocen* OR inmate* OR juris* OR jail* OR judge* OR judic* OR juror* OR juries OR jury OR justice OR law* OR legal* OR legislat* OR litigat* OR "low security" OR "low-security" OR magistrate* OR mandat* OR mitigat* OR marshal* OR misdem* OR "medium security*" OR "medium-security*" OR offend* OR offence* OR officer* OR official* OR ordinance OR parole* OR pardon* OR penal* OR plea* OR pre-admiss* OR pre-arrest* OR pre-imprison* OR pre-trial* OR precedent* OR prevent* OR prison* OR probat* OR prohibit* OR prosecut* OR punish* OR recividis* OR rehab* OR reintegrat* OR remand* OR reoffend* OR "re-offend*" OR ruling* OR sanction* OR sentenc* OR solicitor* OR statut* OR subpoena* OR supervis* OR surveil* OR suspect* OR testif* OR testimon* OR trial* OR tribunal* OR verdict* OR victim* OR witness*) ) OR AB ( (accused OR acquit* OR adjourn* OR adjudicat* OR admiss* OR affida* OR appeal* OR appellate OR apprehend* OR arbitrat* OR arraign* OR arrest* OR attorney* OR authorit* OR bail* OR barrister* OR breach* OR "case manage*" OR "case-manage*" OR caution* OR charge* OR clerk* OR confinement* OR convict* OR coroner* OR correction* OR court* OR crime* OR criminal* OR "cross examin*" OR "cross-examin*" OR custod* OR defendant* OR defense OR defence OR detain* OR detention* OR deter* OR divert* OR diversion* OR enforc* OR execut* OR felon* OR forensic* OR gaol* OR guilt* OR "high security" OR "high-security" OR "halfway house" OR "halfway-house" OR imprison* OR incarcerat* OR indict* OR infract* OR infring* OR injunct* OR inquest* OR innocen* OR inmate* OR juris* OR jail* OR judge* OR judic* OR juror* OR juries OR jury OR justice OR law* OR legal* OR legislat* OR litigat* OR "low security" OR "low-security" OR magistrate* OR mandat* OR mitigat* OR marshal* OR misdem* OR "medium security*" OR "medium-security*" OR offend* OR offence* OR officer* OR official* OR ordinance OR parole* OR pardon* OR penal* OR plea* OR pre-admiss* OR pre-arrest* OR pre-imprison* OR pre-trial* OR precedent* OR prevent* OR prison* OR probat* OR prohibit* OR prosecut* OR punish* OR recividis* OR rehab* OR reintegrat* OR remand* OR reoffend* OR "re-offend*" OR ruling* OR sanction* OR sentenc* OR solicitor* OR statut* OR subpoena* OR supervis* OR surveil* OR suspect* OR testif* OR testimon* OR trial* OR tribunal* OR verdict* OR victim* OR witness*) ) OR KW ((accused OR acquit* OR adjourn* OR adjudicat* OR admiss* OR affida* OR appeal* OR appellate OR apprehend* OR arbitrat* OR arraign* OR arrest* OR attorney* OR authorit* OR bail* OR barrister* OR breach* OR "case manage*" OR "case-manage*" OR caution* OR charge* OR clerk* OR confinement* OR convict* OR coroner* OR correction* OR court* OR crime* OR criminal* OR "cross examin*" OR "cross-examin*" OR custod* OR defendant* OR defense OR defence OR detain* OR detention* OR deter* OR divert* OR diversion* OR enforc* OR execut* OR felon* OR forensic* OR gaol* OR guilt* OR "high security" OR "high-security" OR "halfway house" OR "halfway-house" OR imprison* OR incarcerat* OR indict* OR infract* OR infring* OR injunct* OR inquest* OR innocen* OR inmate* OR juris* OR jail* OR judge* OR judic* OR juror* OR juries OR jury OR justice OR law* OR legal* OR legislat* OR litigat* OR "low security" OR "low-security" OR magistrate* OR mandat* OR mitigat* OR marshal* OR misdem* OR "medium security*" OR "medium-security*" OR offend* OR offence* OR officer* OR official* OR ordinance OR parole* OR pardon* OR penal* OR plea* OR pre-admiss* OR pre-arrest* OR pre-imprison* OR pre-trial* OR precedent* OR prevent* OR prison* OR probat* OR prohibit* OR prosecut* OR punish* OR recividis* OR rehab* OR reintegrat* OR remand* OR reoffend* OR "re-offend*" OR ruling* OR sanction* OR sentenc* OR solicitor* OR statut* OR subpoena* OR supervis* OR surveil* OR suspect* OR testif* OR testimon* OR trial* OR tribunal* OR verdict* OR victim* OR witness*)) |
| 3 | TI ( ("comparison condition*" OR "comparison-condition*" OR "comparison group*" OR "comparison-group*" OR "control condition*" OR "control-condition*" OR "control group*" OR "control-group*" OR effective OR efficac* OR evaluat* OR experiment* OR intervent* OR "matched group*" OR "matched-group*" OR program* OR "quasi-experiment*" OR "quasiexperiment*" OR "quasi experiment*" OR random* OR RCT OR treatment* OR trial*) ) OR AB ( ("comparison condition*" OR "comparison-condition*" OR "comparison group*" OR "comparison-group*" OR "control condition*" OR "control-condition*" OR "control group*" OR "control-group*" OR effective OR efficac* OR evaluat* OR experiment* OR intervent* OR "matched group*" OR "matched-group*" OR program* OR "quasi-experiment*" OR "quasiexperiment*" OR "quasi experiment*" OR random* OR RCT OR treatment* OR trial*) ) OR KW ( ("comparison condition*" OR "comparison-condition*" OR "comparison group*" OR "comparison-group*" OR "control condition*" OR "control-condition*" OR "control group*" OR "control-group*" OR effective OR efficac* OR evaluat* OR experiment* OR intervent* OR "matched group*" OR "matched-group*" OR program* OR "quasi-experiment*" OR "quasiexperiment*" OR "quasi experiment*" OR random* OR RCT OR treatment* OR trial*) ) |
| 4 | 1 and 2 and 3 |
| 5 | 4 and Limiters - Publication Date: 20020101-20211231 |
| 6 | 5 and Limiters - Document Type: Abstract, Article, Book, Book Chapter, Case Study, Essay, Excerpt, Opinion, Other, Proceeding, Report |
| **PsycEXTRA (OVID)** | |
| **Search date** | 18/07/22 |
| **Search notes** | APA PsycExtra <1806 to May 2022> |
| 1 | (extremis* or "far left*" or "far-left*" or "far right*" or "far-right*" or "foreign fight*" or "foreign-fight*" or "freedom fight*" or "freedom-fight*" or guerrilla or "homeland security" or "ideological violence*" or "ideologically motivat*" or "ideologically-motivat*" or indoctrinat* or "left wing*" or "left-wing*" or "lone wol*" or "lone-wol*" or militant* or "national security" or "political violence*" or "politically motivat*" or "politically-motivat*" or radicali* or rebel* or "religious violence*" or "religiously motivat*" or "religiously-motivat*" or "right wing*" or "right-wing*" or "single issue*" or "single-issue*" or supremacis* or terror* or vigilante* or vigilantism or deradicali* or "de-radicali*" or "counter-terror*" or counterterror* or "counter-extremis*" or counterextremis* or separatis* or militia* or jihad*).ab,hw,id,ot,ti. |
| 2 | (accused or acquit* or adjourn* or adjudicat* or admiss* or affida* or appeal* or appellate or apprehend* or arbitrat* or arraign* or arrest* or attorney* or authorit* or bail* or barrister* or breach* or "case manage*" or "case-manage*" or caution* or charge* or clerk* or confinement* or convict* or coroner* or correction* or court* or crime* or criminal* or "cross examin*" or "cross-examin*" or custod* or defendant* or defense or defence or detain* or detention* or deter* or divert* or diversion* or enforc* or execut* or felon* or forensic* or gaol* or guilt* or "high security" or "high-security" or "halfway house" or "halfway-house" or imprison* or incarcerat* or indict* or infract* or infring* or injunct* or inquest* or innocen* or inmate* or juris* or jail* or judge* or judic* or juror* or juries or jury or justice or law* or legal* or legislat* or litigat* or "low security" or "low-security" or magistrate* or mandat* or mitigat* or marshal* or misdem* or "medium security*" or "medium-security*" or offend* or offence* or officer* or official* or ordinance or parole* or pardon* or penal* or plea* or pre-admiss* or pre-arrest* or pre-imprison* or pre-trial* or precedent* or prevent* or prison* or probat* or prohibit* or prosecut* or punish* or recividis* or rehab* or reintegrat* or remand* or reoffend* or "re-offend*" or ruling* or sanction* or sentenc* or solicitor* or statut* or subpoena* or supervis* or surveil* or suspect* or testif* or testimon* or trial* or tribunal* or verdict* or victim* or witness*).ab,hw,id,ot,ti. |
| 3 | ("comparison condition*" or "comparison-condition*" or "comparison group*" or "comparison-group*" or "control condition*" or "control-condition*" or "control group*" or "control-group*" or effective or efficac* or evaluat* or experiment* or intervent* or "matched group*" or "matched-group*" or program* or "quasi-experiment*" or "quasiexperiment*" or "quasi experiment*" or random* or RCT or treatment* or trial).ab,hw,id,ot,ti. |
| 4 | 1 and 2 and 3 |
| 5 | limit 4 to yr="2002 - 2021" |
| **JStor** | |
| **Search date** | 14/07/2022 |
| **Search notes** | Specific search in the following journals not indexed in other databases: Counter Terrorist Trends & Analysis (CTTA), International Journal of Peace Studies, Prism  Each search box has a maximum character limit of 200. Does not seem to support searching in multiple boxes if the total characters of the 2 boxes are over 200, and/or if one box is over 200 characters. Therefore, the search was modified to include only the research design terms (as these are terrorism-focused journals). Note that only 10% of records in Jstor have an abstract. Used the "all fields" search filter, because this seemed more comprehensive. The search did not allow for filtering by multiple journals at one time so the journal filter was applied separately for each of the three journals. |
| 1 | effective OR efficac* OR evaluat* OR experiment* OR intervent* OR program* OR random* OR RCT OR treatment* OR "matched group*" OR "control group" OR "comparison group" OR "control condition" OR "comparison condition" |
| 2 | Filtered by all content |
| 3 | Filtered by date 2002/01/01 to 2021/12/31 |
| 4 | Then 3 separate filters for each journal name (using the checkbox option on journal, NOT the free-text journal box) |
| **Law Journals Library (HeinOnline)** | |
| **Search date** | 15/07/2022 |
| **Search notes** | A search of all 3 sets of terms on the "text" field returned an unmanageable number of results. Therefore, we employed a similar approach to that used in the GPD, which uses the policing terms on the title field and the design terms on the text field. In this case, we searched the terrorism terms on the title field and the design and criminal justice system terms on the text field. |
| 1 | (((title:(extremis* OR "far left*" OR "far-left*" OR "far right*" OR "far-right*" OR "foreign fight*" OR "foreign-fight*" OR "freedom fight*" OR "freedom-fight*" OR guerrilla OR "homeland security" OR "ideological violence*" OR "ideologically motivat*" OR "ideologically-motivat*" OR indoctrinat* OR "left wing*" OR "left-wing*" OR "lone wol*" OR "lone-wol*" OR militant* OR "national security" OR "political violence*" OR "politically motivat*" OR "politically-motivat*" OR radicali* OR rebel* OR "religious violence*" OR "religiously motivat*" OR "religiously-motivat*" OR "right wing*" OR "right-wing*" OR "single issue*" OR "single-issue*" OR supremacis* OR terror* OR vigilante* OR vigilantism OR deradicali* OR "de-radicali*" OR "counter-terror*" OR counterterror* OR "counter-extremis*" OR counterextremis* OR separatis* OR militia* OR jihad*) AND ("comparison condition*" OR "comparison-condition*" OR "comparison group*" OR "comparison-group*" OR "control condition*" OR "control-condition*" OR "control group*" OR "control-group*" OR effective OR efficac* OR evaluat* OR experiment* OR intervent* OR "matched group*" OR "matched-group*" OR program* OR "quasi-experiment*" OR "quasiexperiment*" OR "quasi experiment*" OR random* OR RCT OR treatment* OR trial*)) AND (accused OR acquit* OR adjourn* OR adjudicat* OR admiss* OR affida* OR appeal* OR appellate OR apprehend* OR arbitrat* OR arraign* OR arrest* OR attorney* OR authorit* OR bail* OR barrister* OR breach* OR "case manage*" OR "case-manage*" OR caution* OR charge* OR clerk* OR confinement* OR convict* OR coroner* OR correction* OR court* OR crime* OR criminal* OR "cross examin*" OR "cross-examin*" OR custod* OR defendant* OR defense OR defence OR detain* OR detention* OR deter* OR divert* OR diversion* OR enforc* OR execut* OR felon* OR forensic* OR gaol* OR guilt* OR "high security" OR "high-security" OR "halfway house" OR "halfway-house" OR imprison* OR incarcerat* OR indict* OR infract* OR infring* OR injunct* OR inquest* OR innocen* OR inmate* OR juris* OR jail* OR judge* OR judic* OR juror* OR juries OR jury OR justice OR law* OR legal* OR legislat* OR litigat* OR "low security" OR "low-security" OR magistrate* OR mandat* OR mitigat* OR marshal* OR misdem* OR "medium security*" OR "medium-security*" OR offend* OR offence* OR officer* OR official* OR ordinance OR parole* OR pardon* OR penal* OR plea* OR pre-admiss* OR pre-arrest* OR pre-imprison* OR pre-trial* OR precedent* OR prevent* OR prison* OR probat* OR prohibit* OR prosecut* OR punish* OR recividis* OR rehab* OR reintegrat* OR remand* OR reoffend* OR "re-offend*" OR ruling* OR sanction* OR sentenc* OR solicitor* OR statut* OR subpoena* OR supervis* OR surveil* OR suspect* OR testif* OR testimon* OR trial* OR tribunal* OR verdict* OR victim* OR witness*))) |
| 2 | According to the document type definitions, filtered by only: Articles, notes, chapter, and Reports & Testimonies: Report. See https://help-heinonline-org.ezproxy.library.uq.edu.au/kb/what-are-the-definitions-of-the-content-section-types-in-heinonline-2/ |
| 3 | Filter results by: Law Journals Library |
| 4 | Limited date: 2002 - 2021 |
| **Criminal Justice & Criminology (HeinOnline)** | |
| **Search date** | 18/07/2022 |
| **Search notes** | A search of all 3 sets of terms on the "text" field returned an unmanageable number of results. Therefore, we employed a similar approach to that used in the GPD, which uses the policing terms on the title field and the design terms on the text field. In this case, we searched the terrorism terms on the title field and the design and criminal justice system terms on the text field. |
| 1 | (((title:(extremis* OR "far left*" OR "far-left*" OR "far right*" OR "far-right*" OR "foreign fight*" OR "foreign-fight*" OR "freedom fight*" OR "freedom-fight*" OR guerrilla OR "homeland security" OR "ideological violence*" OR "ideologically motivat*" OR "ideologically-motivat*" OR indoctrinat* OR "left wing*" "left-wing*" OR "lone wol*" OR "lone-wol*"OR militant* OR "national security" OR "political violence*" OR "politically motivat*" OR "politically-motivat*" OR radicali* OR rebel* OR "religious violence*" OR "religiously motivat*" OR "religiously-motivat*" OR "right wing*" OR "right-wing*" OR "single issue*" OR "single-issue*" OR supremacis* OR terror* OR vigilante* OR vigilantism OR deradicali* OR "de-radicali*" OR "counter-terror*" OR counterterror* OR "counter-extremis*" OR counterextremis* OR separatis* OR militia* OR jihad*) AND ("comparison condition*" OR "comparison-condition*" OR "comparison group*" OR "comparison-group*" OR "control condition*" OR "control-condition*" OR "control group*" OR "control-group*" OR effective OR efficac* OR evaluat* OR experiment* OR intervent* OR "matched group*" OR "matched-group*" OR program* OR "quasi-experiment*" OR "quasiexperiment*" OR "quasi experiment*" OR random* OR RCT OR treatment* OR trial*)) AND (accused OR acquit* OR adjourn* OR adjudicat* OR admiss* OR affida* OR appeal* OR appellate OR apprehend* OR arbitrat* OR arraign* OR arrest* OR attorney* OR authorit* OR bail* OR barrister* OR breach* OR "case manage*" OR "case-manage*" OR caution* OR charge* OR clerk* OR confinement* OR convict* OR coroner* OR correction* OR court* OR crime* OR criminal* OR "cross examin*" OR "cross-examin*" OR custod* OR defendant* OR defense OR defence OR detain* OR detention* OR deter* OR divert* OR diversion* OR enforc* OR execut* OR felon* OR forensic* OR gaol* OR guilt* OR "high security" OR "high-security" OR "halfway house" OR "halfway-house" OR imprison* OR incarcerat* OR indict* OR infract* OR infring* OR injunct* OR inquest* OR innocen* OR inmate* OR juris* OR jail* OR judge* OR judic* OR juror* OR juries OR jury OR justice OR law* OR legal* OR legislat* OR litigat* OR "low security" OR "low-security" OR magistrate* OR mandat* OR mitigat* OR marshal* OR misdem* OR "medium security*" OR "medium-security*" OR offend* OR offence* OR officer* OR official* OR ordinance OR parole* OR pardon* OR penal* OR plea* OR pre-admiss* OR pre-arrest* OR pre-imprison* OR pre-trial* OR precedent* OR prevent* OR prison* OR probat* OR prohibit* OR prosecut* OR punish* OR recividis* OR rehab* OR reintegrat* OR remand* OR reoffend* OR "re-offend*" OR ruling* OR sanction* OR sentenc* OR solicitor* OR statut* OR subpoena* OR supervis* OR surveil* OR suspect* OR testif* OR testimon* OR trial* OR tribunal* OR verdict* OR victim* OR witness*))) |
| 2 | According to the document type definitions, filtered by only: Articles, notes, chapter, and Reports & Testimonies: Report. See https://help-heinonline-org.ezproxy.library.uq.edu.au/kb/what-are-the-definitions-of-the-content-section-types-in-heinonline-2/ |
| 3 | Limited date 2002 - 2021 |
| **Foreign & International Law Database (HeinOnline)** | |
| **Search date** | 18/07/2022 |
| **Search notes** | A search of all 3 sets of terms on the "text" field returned an unmanageable number of results. Therefore, we employed a similar approach to that used in the GPD, which uses the policing terms on the title field and the design terms on the text field. In this case, we searched the terrorism terms on the title field and the design and criminal justice system terms on the text field. |
| 1 | (((title:(extremis* OR "far left*" OR "far-left*" OR "far right*" OR "far-right*" OR "foreign fight*" OR "foreign-fight*" OR "freedom fight*" OR "freedom-fight*" OR guerrilla OR "homeland security" OR "ideological violence*" OR "ideologically motivat*" OR "ideologically-motivat*" OR indoctrinat* OR "left wing*" "left-wing*" OR "lone wol*" OR "lone-wol*"OR militant* OR "national security" OR "political violence*" OR "politically motivat*" OR "politically-motivat*" OR radicali* OR rebel* OR "religious violence*" OR "religiously motivat*" OR "religiously-motivat*" OR "right wing*" OR "right-wing*" OR "single issue*" OR "single-issue*" OR supremacis* OR terror* OR vigilante* OR vigilantism OR deradicali* OR "de-radicali*" OR "counter-terror*" OR counterterror* OR "counter-extremis*" OR counterextremis* OR separatis* OR militia* OR jihad*) AND ("comparison condition*" OR "comparison-condition*" OR "comparison group*" OR "comparison-group*" OR "control condition*" OR "control-condition*" OR "control group*" OR "control-group*" OR effective OR efficac* OR evaluat* OR experiment* OR intervent* OR "matched group*" OR "matched-group*" OR program* OR "quasi-experiment*" OR "quasiexperiment*" OR "quasi experiment*" OR random* OR RCT OR treatment* OR trial*)) AND (accused OR acquit* OR adjourn* OR adjudicat* OR admiss* OR affida* OR appeal* OR appellate OR apprehend* OR arbitrat* OR arraign* OR arrest* OR attorney* OR authorit* OR bail* OR barrister* OR breach* OR "case manage*" OR "case-manage*" OR caution* OR charge* OR clerk* OR confinement* OR convict* OR coroner* OR correction* OR court* OR crime* OR criminal* OR "cross examin*" OR "cross-examin*" OR custod* OR defendant* OR defense OR defence OR detain* OR detention* OR deter* OR divert* OR diversion* OR enforc* OR execut* OR felon* OR forensic* OR gaol* OR guilt* OR "high security" OR "high-security" OR "halfway house" OR "halfway-house" OR imprison* OR incarcerat* OR indict* OR infract* OR infring* OR injunct* OR inquest* OR innocen* OR inmate* OR juris* OR jail* OR judge* OR judic* OR juror* OR juries OR jury OR justice OR law* OR legal* OR legislat* OR litigat* OR "low security" OR "low-security" OR magistrate* OR mandat* OR mitigat* OR marshal* OR misdem* OR "medium security*" OR "medium-security*" OR offend* OR offence* OR officer* OR official* OR ordinance OR parole* OR pardon* OR penal* OR plea* OR pre-admiss* OR pre-arrest* OR pre-imprison* OR pre-trial* OR precedent* OR prevent* OR prison* OR probat* OR prohibit* OR prosecut* OR punish* OR recividis* OR rehab* OR reintegrat* OR remand* OR reoffend* OR "re-offend*" OR ruling* OR sanction* OR sentenc* OR solicitor* OR statut* OR subpoena* OR supervis* OR surveil* OR suspect* OR testif* OR testimon* OR trial* OR tribunal* OR verdict* OR victim* OR witness*))) |
| 2 | According to the document type definitions, filtered by only: Articles, notes, chapters. See https://help-heinonline-org.ezproxy.library.uq.edu.au/kb/what-are-the-definitions-of-the-content-section-types-in-heinonline-2/ |
| 3 | Limited date 2002 - 2021 |
| **United Nations Law Collection (HeinOnline)** | |
| **Search date** | 18/07/2022 |
| **Search notes** | A search of all 3 sets of terms on the "text" field returned an unmanageable number of results. Therefore, we employed a similar approach to that used in the GPD, which uses the policing terms on the title field and the design terms on the text field. In this case, we searched the terrorism terms on the title field and the design and criminal justice system terms on the text field. |
| 1 | (((title:(extremis* OR "far left*" OR "far-left*" OR "far right*" OR "far-right*" OR "foreign fight*" OR "foreign-fight*" OR "freedom fight*" OR "freedom-fight*" OR guerrilla OR "homeland security" OR "ideological violence*" OR "ideologically motivat*" OR "ideologically-motivat*" OR indoctrinat* OR "left wing*" "left-wing*" OR "lone wol*" OR "lone-wol*"OR militant* OR "national security" OR "political violence*" OR "politically motivat*" OR "politically-motivat*" OR radicali* OR rebel* OR "religious violence*" OR "religiously motivat*" OR "religiously-motivat*" OR "right wing*" OR "right-wing*" OR "single issue*" OR "single-issue*" OR supremacis* OR terror* OR vigilante* OR vigilantism OR deradicali* OR "de-radicali*" OR "counter-terror*" OR counterterror* OR "counter-extremis*" OR counterextremis* OR separatis* OR militia* OR jihad*) AND ("comparison condition*" OR "comparison-condition*" OR "comparison group*" OR "comparison-group*" OR "control condition*" OR "control-condition*" OR "control group*" OR "control-group*" OR effective OR efficac* OR evaluat* OR experiment* OR intervent* OR "matched group*" OR "matched-group*" OR program* OR "quasi-experiment*" OR "quasiexperiment*" OR "quasi experiment*" OR random* OR RCT OR treatment* OR trial*)) AND (accused OR acquit* OR adjourn* OR adjudicat* OR admiss* OR affida* OR appeal* OR appellate OR apprehend* OR arbitrat* OR arraign* OR arrest* OR attorney* OR authorit* OR bail* OR barrister* OR breach* OR "case manage*" OR "case-manage*" OR caution* OR charge* OR clerk* OR confinement* OR convict* OR coroner* OR correction* OR court* OR crime* OR criminal* OR "cross examin*" OR "cross-examin*" OR custod* OR defendant* OR defense OR defence OR detain* OR detention* OR deter* OR divert* OR diversion* OR enforc* OR execut* OR felon* OR forensic* OR gaol* OR guilt* OR "high security" OR "high-security" OR "halfway house" OR "halfway-house" OR imprison* OR incarcerat* OR indict* OR infract* OR infring* OR injunct* OR inquest* OR innocen* OR inmate* OR juris* OR jail* OR judge* OR judic* OR juror* OR juries OR jury OR justice OR law* OR legal* OR legislat* OR litigat* OR "low security" OR "low-security" OR magistrate* OR mandat* OR mitigat* OR marshal* OR misdem* OR "medium security*" OR "medium-security*" OR offend* OR offence* OR officer* OR official* OR ordinance OR parole* OR pardon* OR penal* OR plea* OR pre-admiss* OR pre-arrest* OR pre-imprison* OR pre-trial* OR precedent* OR prevent* OR prison* OR probat* OR prohibit* OR prosecut* OR punish* OR recividis* OR rehab* OR reintegrat* OR remand* OR reoffend* OR "re-offend*" OR ruling* OR sanction* OR sentenc* OR solicitor* OR statut* OR subpoena* OR supervis* OR surveil* OR suspect* OR testif* OR testimon* OR trial* OR tribunal* OR verdict* OR victim* OR witness*))) |
| 2 | According to the document type definitions, filtered by only: Articles, notes. See https://help-heinonline-org.ezproxy.library.uq.edu.au/kb/what-are-the-definitions-of-the-content-section-types-in-heinonline-2/ |
| 3 | Limited date 2002 - 2021 |
| **Australian Criminology Database and AGIS Plus Text (Informit)** | |
| **Search date** | 14/07/2022 |
| **Search notes** | Searched both databases simultaneously |
| 1 | [All Fields: extremis* OR All Fields: 'far left*' OR All Fields: 'far-left*' OR All Fields: 'far right*' OR All Fields: 'far-right*' OR All Fields: 'foreign fight*' OR All Fields: 'foreign-fight*' OR All Fields: 'freedom fight*' OR All Fields: 'freedom-fight*' OR All Fields: guerrilla OR All Fields: 'homeland security' OR All Fields: 'ideological violence*' OR All Fields: 'ideologically motivat*' OR All Fields: 'ideologically-motivat*' OR All Fields: indoctrinat* OR All Fields: 'left wing*' OR All Fields: 'left-wing*' OR All Fields: 'lone wol*' OR All Fields: 'lone-wol*' OR All Fields: militant* OR All Fields: 'national security' OR All Fields: 'political violence*' OR All Fields: 'politically motivat*' OR All Fields: 'politically-motivat*' OR All Fields: radicali* OR All Fields: rebel* OR All Fields: 'religious violence*' OR All Fields: 'religiously motivat*' OR All Fields: 'religiously-motivat*' OR All Fields: 'right wing*' OR All Fields: 'right-wing*' OR All Fields: 'single issue*' OR All Fields: 'single-issue*' OR All Fields: supremacis* OR All Fields: terror* OR All Fields: vigilante* OR All Fields: vigilantism OR All Fields: deradicali* OR All Fields: 'de-radicali*' OR All Fields: 'counter-terror*' OR All Fields: counterterror* OR All Fields: 'counter-extremis*' OR All Fields: counterextremis* OR All Fields: separatis* OR All Fields: militia* OR All Fields: jihad*] AND [All Fields: 'comparison condition*' OR All Fields: 'comparison-condition*' OR All Fields: 'comparison group*' OR All Fields: 'comparison-group*' OR All Fields: 'control condition*' OR All Fields: 'control-condition*' OR All Fields: 'control group*' OR All Fields: 'control-group*' OR All Fields: effective OR All Fields: efficac* OR All Fields: evaluat* OR All Fields: experiment* OR All Fields: intervent* OR All Fields: 'matched group*' OR All Fields: 'matched-group*' OR All Fields: program* OR All Fields: 'quasi-experiment*' OR All Fields: 'quasiexperiment*' OR All Fields: 'quasi experiment*' OR All Fields: random* OR All Fields: rct OR All Fields: treatment* OR All Fields: trial*] AND [All Fields: accused OR All Fields: acquit* OR All Fields: adjourn* OR All Fields: adjudicat* OR All Fields: admiss* OR All Fields: affida* OR All Fields: appeal* OR All Fields: appellate OR All Fields: apprehend* OR All Fields: arbitrat* OR All Fields: arraign* OR All Fields: arrest* OR All Fields: attorney* OR All Fields: authorit* OR All Fields: bail* OR All Fields: barrister* OR All Fields: breach* OR All Fields: 'case manage*' OR All Fields: 'case-manage*' OR All Fields: caution* OR All Fields: charge* OR All Fields: clerk* OR All Fields: confinement* OR All Fields: convict* OR All Fields: coroner* OR All Fields: correction* OR All Fields: court* OR All Fields: crime* OR All Fields: criminal* OR All Fields: 'cross examin*' OR All Fields: 'cross-examin*' OR All Fields: custod* OR All Fields: defendant* OR All Fields: defense OR All Fields: defence OR All Fields: detain* OR All Fields: detention* OR All Fields: deter* OR All Fields: divert* OR All Fields: diversion* OR All Fields: enforc* OR All Fields: execut* OR All Fields: felon* OR All Fields: forensic* OR All Fields: gaol* OR All Fields: guilt* OR All Fields: 'high security' OR All Fields: 'high-security' OR All Fields: 'halfway house' OR All Fields: 'halfway-house' OR All Fields: imprison* OR All Fields: incarcerat* OR All Fields: indict* OR All Fields: infract* OR All Fields: infring* OR All Fields: injunct* OR All Fields: inquest* OR All Fields: innocen* OR All Fields: inmate* OR All Fields: juris* OR All Fields: jail* OR All Fields: judge* OR All Fields: judic* OR All Fields: juror* OR All Fields: juries OR All Fields: jury OR All Fields: justice OR All Fields: law* OR All Fields: legal* OR All Fields: legislat* OR All Fields: litigat* OR All Fields: 'low security' OR All Fields: 'low-security' OR All Fields: magistrate* OR All Fields: mandat* OR All Fields: mitigat* OR All Fields: marshal* OR All Fields: misdem* OR All Fields: 'medium security*' OR All Fields: 'medium-security*' OR All Fields: offend* OR All Fields: offence* OR All Fields: officer* OR All Fields: official* OR All Fields: ordinance OR All Fields: parole* OR All Fields: pardon* OR All Fields: penal* OR All Fields: plea* OR All Fields: pre-admiss* OR All Fields: pre-arrest* OR All Fields: pre-imprison* OR All Fields: pre-trial* OR All Fields: precedent* OR All Fields: prevent* OR All Fields: prison* OR All Fields: probat* OR All Fields: prohibit* OR All Fields: prosecut* OR All Fields: punish* OR All Fields: recividis* OR All Fields: rehab* OR All Fields: reintegrat* OR All Fields: remand* OR All Fields: reoffend* OR All Fields: 're-offend*' OR All Fields: ruling* OR All Fields: sanction* OR All Fields: sentenc* OR All Fields: solicitor* OR All Fields: statut* OR All Fields: subpoena* OR All Fields: supervis* OR All Fields: surveil* OR All Fields: suspect* OR All Fields: testif* OR All Fields: testimon* OR All Fields: trial* OR All Fields: tribunal* OR All Fields: verdict* OR All Fields: victim* OR All Fields: witness*] AND Publication Date: (01/01/2002 TO 31/12/2021) AND Resource Type: Conference OR Book OR Report OR Journal |
| **Criminal Justice, Dissertations & Theses Global, Digital National Security Archive, PTSDPubs, Social Science Database, &**  **Sociological Abstracts (incl. Social Services Abstracts) (ProQuest)** | |
| **Search date** | 14/07/2022 |
| **Search notes** | Searched all databases simultaneously. NOFT = anywhere except full-text. |
| 1 | (NOFT(extremis* OR "far left*" OR "far-left*" OR "far right*" OR "far-right*" OR "foreign fight*" OR "foreign-fight*" OR "freedom fight*" OR "freedom-fight*" OR guerrilla OR "homeland security" OR "ideological violence*" OR "ideologically motivat*" OR "ideologically-motivat*" OR indoctrinat* OR "left wing*" OR "left-wing*" OR "lone wol*" OR "lone-wol*" OR militant* OR "national security" OR "political violence*" OR "politically motivat*" OR "politically-motivat*" OR radicali* OR rebel* OR "religious violence*" OR "religiously motivat*" OR "religiously-motivat*" OR "right wing*" OR "right-wing*" OR "single issue*" OR "single-issue*" OR supremacis* OR terror* OR vigilante* OR vigilantism OR deradicali* OR "de-radicali*" OR "counter-terror*" OR counterterror* OR "counter-extremis*" OR counterextremis* OR separatis* OR militia* OR jihad*) AND NOFT(accused OR acquit* OR adjourn* OR adjudicat* OR admiss* OR affida* OR appeal* OR appellate OR apprehend* OR arbitrat* OR arraign* OR arrest* OR attorney* OR authorit* OR bail* OR barrister* OR breach* OR "case manage*" OR "case-manage*" OR caution* OR charge* OR clerk* OR confinement* OR convict* OR coroner* OR correction* OR court* OR crime* OR criminal* OR "cross examin*" OR "cross-examin*" OR custod* OR defendant* OR defense OR defence OR detain* OR detention* OR deter* OR divert* OR diversion* OR enforc* OR execut* OR felon* OR forensic* OR gaol* OR guilt* OR "high security" OR "high-security" OR "halfway house" OR "halfway-house" OR imprison* OR incarcerat* OR indict* OR infract* OR infring* OR injunct* OR inquest* OR innocen* OR inmate* OR juris* OR jail* OR judge* OR judic* OR juror* OR juries OR jury OR justice OR law* OR legal* OR legislat* OR litigat* OR "low security" OR "low-security" OR magistrate* OR mandat* OR mitigat* OR marshal* OR misdem* OR "medium security*" OR "medium-security*" OR offend* OR offence* OR officer* OR official* OR ordinance OR parole* OR pardon* OR penal* OR plea* OR pre-admiss* OR pre-arrest* OR pre-imprison* OR pre-trial* OR precedent* OR prevent* OR prison* OR probat* OR prohibit* OR prosecut* OR punish* OR recividis* OR rehab* OR reintegrat* OR remand* OR reoffend* OR "re-offend*" OR ruling* OR sanction* OR sentenc* OR solicitor* OR statut* OR subpoena* OR supervis* OR surveil* OR suspect* OR testif* OR testimon* OR trial* OR tribunal* OR verdict* OR victim* OR witness*) AND NOFT("comparison condition*" OR "comparison-condition*" OR "comparison group*" OR "comparison-group*" OR "control condition*" OR "control-condition*" OR "control group*" OR "control-group*" OR effective OR efficac* OR evaluat* OR experiment* OR intervent* OR "matched group*" OR "matched-group*" OR program* OR "quasi-experiment*" OR "quasiexperiment*" OR "quasi experiment*" OR random* OR RCT OR treatment* OR trial*)) NOT (rtype.exact("News" OR "General Information" OR "Book Review" OR "Transcript" OR "Book Review-Favorable" OR "Editorial" OR "NEWSPAPER" OR "Blogs" OR "Interview" OR "Book Review-Comparative" OR "Book Review-Mixed" OR "Letter" OR "Book review" OR "Memorandum" OR "Press Briefing" OR "Speech/Lecture" OR "Department of Defense Directive" OR "Department of Defense Instruction" OR "Film Review-Favorable" OR "Letter To The Editor" OR "Opinions" OR "Product Review-Favorable" OR "Statement" OR "Book Review-Unfavorable" OR "General_Information" OR "Intelligence Community Directive" OR "Speech" OR "Speech Lecture" OR "Television Review-Mixed" OR "Book review-No Opinion" OR "Book_Review-Favorable" OR "Instruction" OR "Intelligence Community Policy Guidance" OR "Television Review-Favorable" OR "Book Review-No Opinion" OR "General information" OR "Introductory Journal Article" OR "Obituary" OR "Testimony" OR "Air Force Instruction" OR "Arts Exhibits Review-Favorable" OR "Arts/Exhibits Review-Comparative" OR "Biographic Sketch" OR "Biography" OR "Budget Document" OR "Checklist" OR "Corrections/Retraction" OR "Correspondence" OR "Credit/Acknowledgement" OR "Credits" OR "Executive Order" OR "Fact Sheet" OR "Film Review-Mixed" OR "Film Review-Unfavorable" OR "Front Matter" OR "Fund/Grant/Fellowship/Award" OR "Hearing" OR "Letter to the Editor") AND pd(20020101-20211231)) |
| **Cochrane Library & Trials (**<https://www.cochranelibrary.com/search>) | |
| **Search date** | 18/08/2022 |
| **Search notes** | Did not search the research design terms given that Cochrane only includes systematic reviews and trials, and the search result is small. |
| 1 | extremis* or "far left*" or "far-left*" or "far right*" or "far-right*" or "foreign fight*" or "foreign-fight*" or "freedom fight*" or "freedom-fight*" or guerrilla or "homeland security" or "ideological violence*" or "ideologically motivat*" or "ideologically-motivat*" or indoctrinat* or "left wing*" or "left-wing*" or "lone wol*" or "lone-wol*" or militant* or "national security" or "political violence*" or "politically motivat*" or "politically-motivat*" or radicali* or rebel* or "religious violence*" or "religiously motivat*" or "religiously-motivat*" or "right wing*" or "right-wing*" or "single issue*" or "single-issue*" or supremacis* or terror* or vigilante* or vigilantism or deradicali* or "de-radicali*" or "counter-terror*" or counterterror* or "counter-extremis*" or counterextremis* or separatis* or militia* or jihad* in Title Abstract Keyword AND accused or acquit* or adjourn* or adjudicat* or admiss* or affida* or appeal* or appellate or apprehend* or arbitrat* or arraign* or arrest* or attorney* or authorit* or bail* or barrister* or breach* or "case manage*" or "case-manage*" or caution* or charge* or clerk* or confinement* or convict* or coroner* or correction* or court* or crime* or criminal* or "cross examin*" or "cross-examin*" or custod* or defendant* or defense or defence or detain* or detention* or deter* or divert* or diversion* or enforc* or execut* or felon* or forensic* or gaol* or guilt* or "high security" or "high-security" or "halfway house" or "halfway-house" or imprison* or incarcerat* or indict* or infract* or infring* or injunct* or inquest* or innocen* or inmate* or juris* or jail* or judge* or judic* or juror* or juries or jury or justice or law* or legal* or legislat* or litigat* or "low security" or "low-security" or magistrate* or mandat* or mitigat* or marshal* or misdem* or "medium security*" or "medium-security*" or offend* or offence* or officer* or official* or ordinance or parole* or pardon* or penal* or plea* or pre-admiss* or pre-arrest* or pre-imprison* or pre-trial* or precedent* or prevent* or prison* or probat* or prohibit* or prosecut* or punish* or recividis* or rehab* or reintegrat* or remand* or reoffend* or "re-offend*" or ruling* or sanction* or sentenc* or solicitor* or statut* or subpoena* or supervis* or surveil* or suspect* or testif* or testimon* or trial* or tribunal* or verdict* or victim* or witness* in Title Abstract Keyword - in Cochrane Reviews, Cochrane Protocols, Trials |
| 2 | Limited by date 01/01/2002 - 31/12/2021 |
| **Campbell Systematic Reviews (**<https://onlinelibrary.wiley.com/journal/18911803>) | |
| **Search date** | 18/08/2022 |
| **Search notes** | Hand searched Campbell reviews Wiley website for intervention reviews relating to terrorism, extremism and/or radicalisation. |
| **Naval Postgraduate School (**<https://calhoun.nps.edu/handle/10945/30382/discover?order=desc&rpp=10&sort_by=dc.date.issued_dt&page=1&group_by=none&etal=0>) | |
| **Search date** | 20/07/2022 |
| **Search notes** | Separate searches conduced in the technical reports collection and the theses and dissertations collection. |
| Technical reports | (extremis* OR "far left*" OR "far-left*" OR "far right*" OR "far-right*" OR "foreign fight*" OR "foreign-fight*" OR "freedom fight*" OR "freedom-fight*" OR guerrilla OR "homeland security" OR "ideological violence*" OR "ideologically motivat*" OR "ideologically-motivat*" OR indoctrinat* OR "left wing*" "left-wing*" OR "lone wol*" OR "lone-wol*"OR militant* OR "national security" OR "political violence*" OR "politically motivat*" OR "politically-motivat*" OR radicali* OR rebel* OR "religious violence*" OR "religiously motivat*" OR "religiously-motivat*" OR "right wing*" OR "right-wing*" OR "single issue*" OR "single-issue*" OR supremacis* OR terror* OR vigilante* OR vigilantism OR deradicali* OR "de-radicali*" OR "counter-terror*" OR counterterror* OR "counter-extremis*" OR counterextremis* OR separatis* OR militia* OR jihad*) AND ("comparison condition*" OR "comparison-condition*" OR "comparison group*" OR "comparison-group*" OR "control condition*" OR "control-condition*" OR "control group*" OR "control-group*" OR effective OR efficac* OR evaluat* OR experiment* OR intervent* OR "matched group*" OR "matched-group*" OR program* OR "quasi-experiment*" OR "quasiexperiment*" OR "quasi experiment*" OR random* OR RCT OR treatment* OR trial*) AND (accused OR acquit* OR adjourn* OR adjudicat* OR admiss* OR affida* OR appeal* OR appellate OR apprehend* OR arbitrat* OR arraign* OR arrest* OR attorney* OR authorit* OR bail* OR barrister* OR breach* OR "case manage*" OR "case-manage*" OR caution* OR charge* OR clerk* OR confinement* OR convict* OR coroner* OR correction* OR court* OR crime* OR criminal* OR "cross examin*" OR "cross-examin*" OR custod* OR defendant* OR defense OR defence OR detain* OR detention* OR deter* OR divert* OR diversion* OR enforc* OR execut* OR felon* OR forensic* OR gaol* OR guilt* OR "high security" OR "high-security" OR "halfway house" OR "halfway-house" OR imprison* OR incarcerat* OR indict* OR infract* OR infring* OR injunct* OR inquest* OR innocen* OR inmate* OR juris* OR jail* OR judge* OR judic* OR juror* OR juries OR jury OR justice OR law* OR legal* OR legislat* OR litigat* OR "low security" OR "low-security" OR magistrate* OR mandat* OR mitigat* OR marshal* OR misdem* OR "medium security*" OR "medium-security*" OR offend* OR offence* OR officer* OR official* OR ordinance OR parole* OR pardon* OR penal* OR plea* OR pre-admiss* OR pre-arrest* OR pre-imprison* OR pre-trial* OR precedent* OR prevent* OR prison* OR probat* OR prohibit* OR prosecut* OR punish* OR recividis* OR rehab* OR reintegrat* OR remand* OR reoffend* OR "re-offend*" OR ruling* OR sanction* OR sentenc* OR solicitor* OR statut* OR subpoena* OR supervis* OR surveil* OR suspect* OR testif* OR testimon* OR trial* OR tribunal* OR verdict* OR victim* OR witness*) |
| Theses | The search returned an unmanageable number of results with difficulties in exporting. Instead, we downloaded metadata for quarterly theses published as far back as they go (2021-2012). This included all theses regardless of topic, but did include complete data for screening (e.g., all titles and abstracts). We converted this metadata from CSV to an RIS file for upload to DistillerSR and screening. |
| **NCJRS (**<https://www.ojp.gov/ncjrs/virtual-library/search>) | |
| **Search date** | 20/07/2022 |
| **Search notes** | We only had access to the free version, and the functionality was quite poor. We conducted individual searches with the following keywords (searched separately) on the title field, with date limiters After:12/2001 and Before:01/2022. We scanned through the lists manually and exported anything to our EndNote library which looked - on the title/abstract - to be a CJS response to terrorism. Note, the website does not like asterisks, but will search word variants if you just enter the start of the word (e.g., entering extremis brings up results for extremism and extremist). |
| 1 | Terms searched individually on title:  Terror  homeland security  Extremis  Radicali  counter-  counterterror  jihad  national security  supremac  ideological  far-right  political violence  lone wol  Vigilant  politically  rebel  militant  deradicali  foreign fight  right-wing  militia  far right  left-wing  guerrilla  indoctrinat  separatis  single issue  single-issue  far left  far-left  freedom fight  freedom-fight  foreign-fight  lone-wol  left wing  right wing  religious violence  religiously  counterextrem  de-radicali |
| **AEA RCT Registry (**<https://www.socialscienceregistry.org/>) | |
| **Search date** | 12/07/2022 |
| **Search notes** | This website does not support Boolean operators in the advanced search. Additionally, searching one of the phrases (e.g., "far right") in quotation marks and/or with a hyphen was returning results where just one of the individual words was present. In other words, it seemed to be ignoring the quotations and hyphens. We tested all terrorism terms, but many either did not produce the correct results around phrasing, and many did not return results related to terrorism. Decided approach would be to conduct searches on the terrorism words individually, download separate RIS files with the results, and then combine results and remove duplicates in EndNote. |
| 1 | *terror* : Title field |
| 2 | *extremis* : Title field |
| 3 | *radicali* : Title field |
| 4 | *guerrilla* : Title field |
| 5 | *militant* : Title field |
| 6 | *vigilant* : Title field |
| 7 | *militia* : Title field |
| 8 | *separatis* : Title field |
| 9 | *jihad* : Title field |
| 10 | *terror* : Abstract field |
| 11 | *extremis* : Abstract field |
| 12 | *radicali* : Abstract field |
| 13 | *guerrilla* : Abstract field |
| 14 | *militant* : Abstract field |
| 15 | *vigilant* : Abstract field |
| 16 | *militia* : Abstract field |
| 17 | *separatis* : Abstract field |
| 18 | *jihad* : Abstract field |
| **WHO Trial Registry (**[https://trialsearch.who.int](https://trialsearch.who.int/)) | |
| **Search date** | 14/07/2022 |
| **Search notes** | The standard search bar searches: Title, Primary sponsor, Health Condition(s), Intervention(s), Countries of recruitment, Main ID, Secondary ID(s). These options are more comprehensive than the advanced search function. |
| 1 | Simple search field: (extremis* OR "far left*" OR "far-left*" OR "far right*" OR "far-right*" OR "foreign fight*" OR "foreign-fight*" OR "freedom fight*" OR "freedom-fight*" OR guerrilla OR "homeland security" OR "ideological violence*" OR "ideologically motivat*" OR "ideologically-motivat*" OR indoctrinat* OR "left wing*" OR "left-wing*" OR "lone wol*" OR "lone-wol*" OR militant* OR "national security" OR "political violence*" OR "politically motivat*" OR "politically-motivat*" OR radicali* OR rebel* OR "religious violence*" OR "religiously motivat*" OR "religiously-motivat*" OR "right wing*" OR "right-wing*" OR "single issue*" OR "single-issue*" OR supremacis* OR terror* OR vigilante* OR vigilantism OR deradicali* OR "de-radicali*" OR "counter-terror*" OR counterterror* OR "counter-extremis*" OR counterextremis* OR separatis* OR militia* OR jihad*) |
| **TRoPHI (**https://eppi.ioe.ac.uk/webdatabases4/Intro.aspx?ID=12) | |
| **Search date** | 14/07/2022 |
| **Search notes** | Used the "free text" field which searches all search fields except for the authors (i.e., it searches titles, abstracts). Searched all terrorism terms individually, then combined the searches for those terms which returned results (which was only terror*, rebel*, "single issue" and "single-issue"). |
| 1 | Freetext (All but Authors): terror* |
| 2 | Freetext (All but Authors): extremis* |
| 3 | Freetext (All but Authors): radicali* |
| 4 | Freetext (All but Authors): "national security" |
| 5 | Freetext (All but Authors): "homeland security" |
| 6 | Freetext (All but Authors): "far left" |
| 7 | Freetext (All but Authors): "far-left" |
| 8 | Freetext (All but Authors): "far right" |
| 9 | Freetext (All but Authors): "far-right" |
| 10 | Freetext (All but Authors): "foreign fight*" |
| 11 | Freetext (All but Authors): "foreign-fight*" |
| 12 | Freetext (All but Authors): guerrilla* |
| 14 | Freetext (All but Authors): indoctrinat* |
| 15 | Freetext (All but Authors): ideological* |
| 16 | Freetext (All but Authors): "left wing" |
| 17 | Freetext (All but Authors): "left-wing" |
| 18 | Freetext (All but Authors): "right wing" |
| 19 | Freetext (All but Authors): "right-wing" |
| 20 | Freetext (All but Authors): "lone wol*" |
| 21 | Freetext (All but Authors): "lone-wol*" |
| 22 | Freetext (All but Authors): militant* |
| 23 | Freetext (All but Authors): "politically motivat*" |
| 24 | Freetext (All but Authors): "politically-motivat*" |
| 25 | Freetext (All but Authors): "political violence" |
| 26 | Freetext (All but Authors): rebel* |
| 27 | Freetext (All but Authors): "religious violence" |
| 28 | Freetext (All but Authors): "religiously motivate*" |
| 29 | Freetext (All but Authors): "religiously-motivate*" |
| 30 | Freetext (All but Authors): "single issue" |
| 31 | Freetext (All but Authors): "single-issue" |
| 32 | Freetext (All but Authors): supremacis* |
| 33 | Freetext (All but Authors): vigilante* |
| 34 | Freetext (All but Authors): vigilantism |
| 35 | Freetext (All but Authors): deradicali* |
| 36 | Freetext (All but Authors): "de-radicali*" |
| 37 | Freetext (All but Authors): "de-radicali*" |
| 38 | Freetext (All but Authors): "counterterror*" |
| 39 | Freetext (All but Authors): "counter-terror*" |
| 40 | Freetext (All but Authors): "counterextremis*" |
| 41 | Freetext (All but Authors): "counter-extremis*" |
| 42 | Freetext (All but Authors): separatis* |
| 43 | Freetext (All but Authors): militia* |
| 44 | Freetext (All but Authors): jihad* |
| 45 | 1 OR 26 OR 30 OR 31 |
| **NIH RePORTER (**<https://reporter.nih.gov/>) | |
| **Search date** | 14/07/2022 |
| **Search notes** | The search filter is limited to 2,500 characters, meaning the full search terms could not be used on this site. Downloaded results across the clinical trials, projects, and publications sections. |
| 1 | Fiscal Year: Active Projects, 2021, 2020, 2019, 2018, 2017, 2016, 2015, 2014, 2013, 2012, 2011, 2010, 2009, 2008, 2007, 2006, 2005, 2004, 2003, 2002 |
| 2 | Text Search: (extremis* OR "far left*" OR "far-left*" OR "far right*" OR "far-right*" OR "foreign fight*" OR "foreign-fight*" OR "freedom fight*" OR "freedom-fight*" OR guerrilla OR "homeland security" OR "ideological violence*" OR "ideologically motivat*" OR "ideologically-motivat*" OR indoctrinat* OR "left wing*" OR "left-wing*" OR "lone wol*" OR "lone-wol*" OR militant* OR "national security" OR "political violence*" OR "politically motivat*" OR "politically-motivat*" OR radicali* OR rebel* OR "religious violence*" OR "religiously motivat*" OR "religiously-motivat*" OR "right wing*" OR "right-wing*" OR "single issue*" OR "single-issue*" OR supremacis* OR terror* OR vigilante* OR vigilantism OR deradicali* OR "de-radicali*" OR "counter-terror*" OR counterterror* OR "counter-extremis*" OR counterextremis* OR separatis* OR militia* OR jihad*) AND (accused OR acquit* OR adjourn* OR adjudicat* OR admiss* OR affida* OR appeal* OR appellate OR apprehend* OR arbitrat* OR arraign* OR arrest* OR attorney* OR authorit* OR bail* OR barrister* OR breach* OR "case manage*" OR "case-manage*" OR caution* OR charge* OR clerk* OR confinement* OR convict* OR coroner* OR correction* OR court* OR crime* OR criminal* OR "cross examin*" OR "cross-examin*" OR custod* OR defendant* OR defense OR defence OR detain* OR detention* OR deter* OR divert* OR diversion* OR enforc* OR execut* OR felon* OR forensic* OR gaol* OR guilt* OR "high security" OR "high-security" OR "halfway house" OR "halfway-house" OR imprison* OR incarcerat* OR indict* OR infract* OR infring* OR injunct* OR inquest* OR innocen* OR inmate* OR juris* OR jail* OR judge* OR judic* OR juror* OR juries OR jury OR justice OR law* OR legal* OR legislat* OR litigat* OR "low security" OR "low-security" OR magistrate* OR mandat* OR mitigat* OR marshal* OR misdem* OR "medium security*" OR "medium-security*" OR offend* OR offence* OR officer* OR official* OR ordinance OR parole* OR pardon* OR penal* OR plea* OR pre-admiss* OR pre-arrest* OR pre-imprison* OR pre-trial* OR precedent* OR prevent* OR prison* OR probat* OR prohibit* OR prosecut* OR punish* OR recividis* OR rehab* OR reintegrat* OR remand* OR reoffend* OR "re-offend*" OR ruling* OR sanction* OR sentenc* OR solicitor* OR statut* OR subpoena* OR supervis* OR surveil* OR suspect* OR testif* OR testimon* OR trial* OR tribunal* OR verdict* OR victim* OR witness*) |
| 3 | Limit to: Project Title, Project Terms, Project Abstracts |
| 4 | 1 AND 2 AND 3 |

## 4 Grey literature search dates and approaches

| Organisation | Website | Search date | Search approach |
| --- | --- | --- | --- |
| Centre of Excellence Defence Against Terrorism | https://www.tmmm.tsk.tr/research.html | 6/06/2022 | Downloaded all citations. |
| Combating Terrorism Center at West Point | https://ctc.usma.edu/reports/?type=major-reports | 6/06/2022 | Downloaded any citations that looked to report on criminal justice or government responses to terrorism/radicalisation/extremism. |
| Impact Europe | <http://impacteurope.eu/publications/?set=1> | 6/06/2022 | Downloaded citations for all external publications & project deliverables. |
| CrimeSolutions | https://crimesolutions.ojp.gov/topics/crime-crime-prevention/subtopic/terrorism | 6/06/2022 | Clicked on the title of each practice and went to "Evidence-base (meta-analyses reviewed)" section. Harvested the citations (there was 1 citation for each of the 2 practices). |
| NSW Corrective Services (Collaborative Reports) | https://correctiveservices.dcj.nsw.gov.au/csnsw-home/resources/research-and-reports/corrections-research-evaluation-and-statistics/collaborative-reports.html | 6/06/2022 | Manually examined the abstracts and downloaded any that looked to be on terrorism/radicalisation/extremism. None were found. |
| Prisons Research Centre | <https://www.prc.crim.cam.ac.uk/publications/research-findings> | 6/06/2022 | Manually examined all publications subsections and downloaded any that looked to be on terrorism/radicalisation/extremism. None were found. |
| Queensland Corrective Services | <https://corrections.qld.gov.au/documents/reviews-and-reports/> | 6/06/2022 | Manually examined all publications and downloaded any that looked to be on terrorism/radicalisation/extremism. None were found. |
| RAND Corporation - Priority Criminal Justice Needs | https://www.rand.org/well-being/justice-policy/projects/priority-criminal-justice-needs/publications.html | 6/06/2022 | Manually examined all publications and downloaded any that looked to be on terrorism/radicalisation/extremism. None were found. |
| Tasmania - Sentencing Advisory Council | https://www.sentencingcouncil.tas.gov.au/reports; https://www.sentencingcouncil.tas.gov.au/projects | 6/06/2022 | Manually examined all publications and downloaded any that looked to be on terrorism/radicalisation/extremism. None were found. |
| Terrorism Research Centre | <https://www.terrorism.org/resources/> | 6/06/2022 | Manually examined the executive summaries for each report, downloaded if they mentioned the criminal justice system. |
| Triangle Center on Terrorism and Homeland Security | https://tcths.sanford.duke.edu/category/reports/ | 6/06/2022 | Manually examined all publications and downloaded any that looked to be on criminal justice system responses to terrorism/radicalisation/extremism. |
| Australian Capital Territory Corrective Services | https://correctiveservices.act.gov.au/about-us/policies-and-publications | 8/06/2022 | Manually examined all publications and downloaded any that looked to be on terrorism/radicalisation/extremism. |
| Correctional Service Canada | https://www.csc-scc.gc.ca/publications/index-eng.shtml | 8/06/2022 | Manually searched through all research topics and special reports. Examined the titles of all publications and download any that looked to be on criminal justice system responses to terrorism/radicalisation/extremism. |
| Global Terrorism Research Centre (Monash University) | <https://www.monash.edu/arts/social-sciences/gtrec/publications> | 8/06/2022 | Manually searched through all sub-sections for publications (i.e., articles, books, etc.), and added anything that looked to be a criminal justice system response to terrorism/extremism/radicalisation. |
| New South Wales Corrective Services (Research Publications) | https://correctiveservices.dcj.nsw.gov.au/csnsw-home/resources/research-and-reports/corrections-research-evaluation-and-statistics.html | 8/06/2022 | Manually examined subsections (on sidebar, the subheaders under "corrections research evaluations and statistics") and downloaded any that looked to be on terrorism/radicalisation/extremism. None were found. |
| Northern Territory Corrective Services | <https://justice.nt.gov.au/attorney-general-and-justice/justice-publications> | 8/06/2022 | Manually examined all publications and downloaded any that looked to be on terrorism/radicalisation/extremism. None were found. |
| NZ Corrective Services | <http://www.corrections.govt.nz/resources/research_and_statistics.html> | 8/06/2022 | Manually examined all publications and downloaded any that looked to be on terrorism/radicalisation/extremism. None were found. |
| South Australia Corrective Services | <https://www.corrections.sa.gov.au/about/our-research> | 8/06/2022 | Manually examined subsections (on sidebar, the subheaders under "our research and initiatives") and downloaded any that looked to be on terrorism/radicalisation/extremism. |
| Tasmanian Corrective Services | https://www.justice.tas.gov.au/publications | 8/06/2022 | Manually examined all publications and downloaded any that looked to be on terrorism/radicalisation/extremism. None were found. |
| Victorian Corrections, Prisons and Parole | <https://www.corrections.vic.gov.au/publications-manuals-and-statistics> | 8/06/2022 | Manually examined all publications and downloaded any that looked to be on terrorism/radicalisation/extremism. None were found. |
| Western Australia Corrective Services | <https://www.correctiveservices.wa.gov.au/about-us/statistics-publications/default.aspx> | 8/06/2022 | Manually examined all publications and downloaded any that looked to be on terrorism/radicalisation/extremism. |
| International Centre for Counter-Terrorism | <https://icct.nl/topic/criminal-justice-response/> | 9/06/2022 | Manually examined all publications and downloaded any that looked to be a criminal justice system response to terrorism/radicalisation/extremism. |
| Radicalisation Awareness Network (RAN) | https://ec.europa.eu/home-affairs/networks/radicalisation-awareness-network-ran/publications_en | 9/06/2022 | Manually examined all publications and downloaded any that looked to be a criminal justice system response to terrorism/radicalisation/extremism. |
| RAND - Correctional education | https://www.rand.org/well-being/justice-policy/portfolios/correctional-education/publications.html | 9/06/2022 | Manually examined all publications and downloaded any that looked to be on terrorism/radicalisation/extremism. None were found. |
| RAND - Courts | <https://www.rand.org/jie/justice-policy/pubs/courts.html> | 9/06/2022 | Manually examined all publications and downloaded any that looked to be on terrorism/radicalisation/extremism. |
| RAND - Better Policing Toolkit | <https://www.rand.org/pubs/tools/TL261/better-policing-toolkit.html> | 10/06/2022 | Manually searched through all practices listed in the toolkit and downloaded any that looked to be on terrorism/radicalisation/extremism. None were found. |
| The Journal of International Security Affairs | <https://security-affairs.com/about/> | 10/06/2022 | Manually examined the publications and downloaded any that looked to be on the criminal justice system. None were found. |
| Department of Homeland Security | <https://www.dhs.gov/topics> | 13/06/2022 | Please have a poke around on the website and see if you can find where they publish their research. I couldn’t find any on the website. If you find any, please include the URL but don't download anything until I have a look |
| International Centre for the Study of Radicalisation | https://icsr.info/publications/reports/ | 13/06/2022 | Manually examined all publications and downloaded any that looked to be a criminal justice system response to terrorism/radicalisation/extremism. |
| What Works Toolkit | <http://whatworks.college.police.uk/toolkit/Pages/Toolkit.aspx> | 14/07/2022 | Manually examined all publications and downloaded any that looked to be on terrorism/radicalisation/extremism. None were found. |
| Global Centre on Cooperative Security | <https://www.globalcenter.org/publications/> | 18/07/2022 | Filtered by each topic area (countering violent extremism; prevention; improving global counterterrorism cooperation; criminal justice and rule of law; radicalisation; rule of law; terrorism financing; women and counter-terrorism; prisons; foreign fighters) and manually searched for anything reporting on the criminal justice system. |
| Hedayah | <http://www.hedayahcenter.org/publications> | 15/07/2022 | Filtered by type: reports and publications. Manually examined the publications and downloaded any that looked to be on the criminal justice system. |
| International Association of Law Enforcement Intelligence Analysts | <https://www.ialeia.org/resources_publications.php> | 15/07/2022 | Manually examined this bibliography and downloaded any that looked to be on terrorism/radicalisation/extremism. |
| Journal of 9/11 Studies | <http://www.journalof911studies.com/J911S/articles/> | 18/07/2022 | Manually examined all publications and downloaded any that looked to be a criminal justice system response to terrorism/radicalisation/extremism. |
| Journal of Security, Intelligence and Resilience Education | <https://jsire.org/about/> | 18/07/2022 | Manually examined the publications and downloaded any that looked to be on the criminal justice system. |
| Royal United Services Institute (RUSI) | <https://rusi.org/> | 19/07/2022 | Went to ‘Explore our Research’, then ‘Publications’, then ‘Reports, Papers & Briefings’. Filtered by topic ‘Countering Terrorism and Violent Extremism' AND ‘Terrorist Financing’. Also filtered by year January 2002 – December 2021. There were 82 articles. Manually examined these and downloaded any that looked to be on the criminal justice system. |
| Centre for Advancing Correctional Excellence | <https://www.gmuace.org/> | 9/06/2022 | Manually examined the summaries of completed projects. This page goes not list publications (just projects), so any summaries that looked to be on criminal justice responses to terrorism/radicalisation/extremism were recorded to be sourced later. The same steps were repeated for the current projects. None were found. |
| National Institute of Corrections | https://nicic.gov/all-library-items | 9/06/2022 | In the 'All library items' search window, entered "terrorism" into the 'tags' box. Filtered 'media type' by document and downloaded all documents in the result. This was tested for all other terrorism terms in our search, with results only for "terrorism" |
| Urban Institute | <https://www.urban.org/policy-centers/justice-policy-center/publications> | 9/06/2022 | Searched within the crime, justice and safety Research Area (https://www.urban.org/research-area/crime-justice-and-safety). Entered the term "terrorism" and filtered by publications. Downloaded all (n=2). This was tested for all other terrorism terms in our search, with results only for "terrorism". |
| Public Safety Canada | <https://www.publicsafety.gc.ca/index-en.aspx> | 13/06/2022 | Four steps were taken. First, in the 'publications and reports' section of the website, filtered by the following topics (one at a time) and downloaded all results: "counter-terrorism", "departmental reports - anti-terrorism". Second, filtered by topic "crime prevention - evidence-based" and downloaded any results that looked to be on terrorism/radicalisation/extremism. Third, filtered by topic "departmental reports - evaluations" AND entered 'terror' in the keyword box (all other terrorism terms were piloted). Fourth, changed the topic field to 'all' and searched "extremi" in the keyword field (all other terrorism terms were piloted; there was total overlap with the terms extremi and terror, so this step was only conduced for extremi) |
| UK Government research | <https://www.gov.uk/search/research-and-statistics> | 13/06/2022 | Five steps were taken based on a thorough test of the search fields and subtopic contents. First, filtered by Research + topic area "crime, justice & law" + sub-topic "counter-extremism" and downloaded all results. Second, filtered by Research + topic area "crime, justice & law" + sub-topic "counter-terrorism" and downloaded all results. Third, filtered by Research + topic area "crime, justice & law" + sub-topic "crime prevention" and downloaded all that looked to be on terrorism/radicalisation/extremism. Fourth, filtered by Research + topic area "crime, justice & law" + sub-topic "policing" and downloaded all that looked to be on terrorism/radicalisation/extremism. Fifth, filtered by Research + topic area "crime, justice & law" + sub-topic "prisons and probation" and downloaded all that looked to be on terrorism/radicalisation/extremism. |
| Journal of Terrorism Research | <https://cvir.st-andrews.ac.uk/articles/search/> | 14/07/2022 | Manually examined all articles in the archive and downloaded any that looked to be on criminal justice system responses to terrorism/radicalisation/extremism. |
| RadicalisationResearch | <https://www.radicalisationresearch.org/> | 15/07/2022 | Manually examined all categories and tags. Determined that potentially relevant studies existed under the tag "intervention" and the categories "prevention", "countering violent extremism", "de-radicalisation", and "uncategoized". Filtered results five times for each of the tags/categories separately, and downloaded all results on the first 3. For "de-radicalisation", and "uncategoized" (which had larger numbers of results), manually examined each citation and downloaded any that looked to be a criminal justice system response to terrorism/radicalisaiton/extremism. |
| National Consortium for the Study of Terrorism and Responses to Terrorism (START) | <https://www.start.umd.edu/> | 18/07/2022 | There is a publications search that allows you to search: Keywords in title or abstract, Publication type, Research Area, Topics, Regions, Authors, Date. The Keywords field does not allow many characters at a time, so is not really conducive to a complex/long search. The research area and topic fields were not particularly useful for our purposes - it is hard to tell where/under what headings any CJS-related research might be categorised. Also, the topic filter consists largely of different types of terrorism, so is also not helpful for that reason. We first filtered the search by publication type to remove ineligible publications (retaining START reports, START white papers, white papers, research reviews, research briefs, reports, presentations, conference papers, book chapters, books, journal articles, and background reports). Then we searched each of the following terms one by one and downloaded all search results: Effective; Evaluat; Intervention; Program; Experiment; Treatment; Efficac; Random; Trial; Comparison group; RCT; Control group; Comparison condition; Comparison-group; Comparison-condition; Control-group; Control condition; Control-condition; Matched group; Matched-group; Quasi-experiment; Quasiexperiment. |
| Note: all searches were restricted by year, and only 2002-2021 data were downloaded from each website. | | | |

## 5 Coding Form

| Concept | Category |
| --- | --- |
| Ref ID |  |
| Study type | - Research study - Research synthesis |
| Research design | - Systematic review (with or without meta-analysis) - RCT - Strong quasi-experimental study |
| Publication status | - Published - Unpublished |
| Study location (country) | *please specify* |
| Study location (region) | - Oceania and antarctica - North West Europe - Southern and Eastern Europe - North Africa and the Middle East - South East Asia - North East Asia - Southern and Central Asia - North America - South America - Central Ameria - Sub-Saharan Africa - Multi-region |
| Country income group | - Low income - Middle income - High income - N/A |
| Implementation setting | - Police operations/facilities - Prison/correctional facilities - Probation/parole - Courts - Community - School - Workplace - Places of worship - Home - Internet - Other |
| Focus of intervention | - Preventing radicalisation - Preventing terrorism - Preventing both |
| Prevention category | - Primary - Secondary - Tertiary - Primary, secondary and tertiary |
| Age of targets | - Youth - Adults - Both |
| Target population | - Criminal justice practitioners - Victims - Communities - Individuals or groups who have been identified as at risk of becoming radicalised or engaging in violent extremism and/or terrorist activity group - Radicalised individuals or groups (including pre-criminal justice involved radicalised individuals) - Individuals or groups who have engaged in violent extremism and/or terrorist activity - Family members of radicalised individuals or individuals who have engaged in violent extremism and/or terrorist activity - Micro places (e.g., street corners, buildings, police beats, street segments) - Macro places (neighbourhoods or larger geographies) |
| Ideology targeted | - Not specified - Right wing - Lone wolf - Single issue - Single issue: eco-terrorism - Far left |
| Intervention categorisation (broad) | - Police - Courts - Prison/correctional facilities - Probation/parole - Multi-agency - Other (e.g. intelligence agencies) |
| Intervention categorisation (specific) | *please specify* |
| Outcome(s) | *please specify* |
